# HELZ-BRCA2 complex resolves R-loops to drive transcription-coupled homologous recombination

**DOI:** 10.1038/s41467-026-75088-4

**Published:** 2026-07-23

**Authors:** Wenjing Li, Bo Wu, Boya Gao, Elizabeth M. Irvin, Arijit Ghosh, Lillian Eliaz, Yuxin Huang, Youngho Kwon, Clara M. Stiefel, Tram Thi Ngoc Nguyen, David Zhao, Humberto Javier Suarez, Tengyang Ni, Salvador Alejo, O’Taveon Fitzgerald, Xuemei Song, Sandip Kumar Rath, Elizabeth V. Wasmuth, David S. Yu, Siyuan Zheng, Justin Leung, Xiaoyu Xue, Hong Wang, Jae-Hoon Ji, Li Lan, Weixing Zhao

**Affiliations:** 1 Department of Biochemistry and Structural Biology, Greehey Children’s Cancer Research Institute, University of Texas Health Science Center at San Antonio, San Antonio, TX USA; 2 Department of Molecular Genetics and Microbiology, School of Medicine, Duke University, Durham, NC USA; 3 Department of Physics, Center for Human Health and the Environment, Toxicology Program, North Carolina State University, Raleigh, NC USA; 4 Department of Chemistry & Biochemistry Materials Science, Engineering, and Commercialization Program, Integrated Molecular and Biophysical Chemistry Program, Texas State University, San Marcos, TX USA; 5 Department of Radiation Oncology, University of Texas Health Science Center at San Antonio, San Antonio, TX USA; 6 Department of Obstetrics & Gynecology, University of Texas Health Science Center at San Antonio, San Antonio, TX USA; 7 Department of Population Health Sciences, University of Texas Health Science Center at San Antonio, San Antonio, TX USA; 8 Department of Radiation Oncology and Winship Cancer Institute, Emory University, Atlanta, GA USA

**Keywords:** Homologous recombination, DNA, RNA, Double-strand DNA breaks

## Abstract

R-loops are transcription-induced, three-stranded nucleic acid structures that, if not properly resolved, can disrupt DNA repair and compromise genome stability. BRCA2, a tumor suppressor vital for homologous recombination (HR), also contributes to R-loop regulation, though the underlying mechanisms remain poorly understood. Here, we identify HELZ as a direct BRCA2 interactor and characterize it as an ssRNA-specific R-loop resolvase. BRCA2 enhances HELZ helicase activity and promotes its recruitment to R-loops. Importantly, HELZ resolves R-loops at DNA double-strand breaks, enabling efficient DNA end resection and HR, particularly within transcriptionally active genomic regions. We further demonstrate that HELZ is critical for R-loop clearance in cancers with elevated transcriptional activity and R-loop accumulation, such as estrogen receptor-positive breast cancer, where it becomes essential for cell survival under estrogen-induced transcriptional stress. These findings establish HELZ as a BRCA2-dependent regulator of R-loop homeostasis and identify it as a potential biomarker and therapeutic target in R-loop-driven malignancies.

## Abstract (web summary)

R-loops can block DNA repair and destabilize genomes. Here, the authors identify HELZ as a BRCA2 partner that removes R-loops at DNA breaks to enable transcription-coupled homologous recombination and support survival of R-loop–rich cancers such as ER+ breast cancer and Ewing sarcoma.

## Introduction

R-loops are three-stranded structures composed of an RNA-DNA hybrid and displaced single-stranded DNA (ssDNA), formed co-transcriptionally and dynamically regulated in cells^1–3^. Although physiological R-loops contribute to transcription, replication, and DNA repair, their accumulation can trigger transcription-replication conflicts, DNA damage, and genome instability^1–8^. Emerging work from the last decade has shown that R-loops also form at DNA double-strand breaks (DSBs), which arise either from hybridization of pre-existing transcripts or from damage-induced de novo transcription pairing with the resected ssDNA, and they modulate DNA damage response and its repair by homologous recombination (HR)^9–14^. HR begins with DNA-end resection at DSBs to generate 3′ ssDNA for RAD51 loading and strand invasion of the sister chromatid, enabling error-free repair^15^. Despite their emerging importance in HR regulation, the mechanisms governing R-loop formation, resolution, and maintenance at DSBs are still not fully understood.

Mutations in *BRCA2* (BReast CAncer gene 2) primarily drive breast cancer, particularly estrogen receptor-positive (ER⁺) subtypes, as well as ovarian cancers^16,17^. BRCA2 is essential for maintaining genomic integrity through its pivotal roles in loading RAD51 onto ssDNA during HR repair of DSBs, as well as in preventing the accumulation of ssDNA gaps and protecting nascent DNA from degradation (replication-fork protection; RFP) at stalled replication forks^15,18–20^. As BRCA2’s obligatory partner, DSS1 stabilizes BRCA2, retains it in the nucleus, and enhances RAD51 loading of RPA-coated ssDNA through its DNA-mimicking interaction with RPA^15,18–25^. Beyond these canonical roles, our recent work indicates that DSS1 also modulates BRCA2’s binding preference for ssDNA over dsDNA, contributing to HR, RFP, and R-loop regulation^26^. Despite growing evidence implicating the BRCA2-DSS1 complex in R-loop homeostasis^22,26–29^, how BRCA2-DSS1 promotes R-loop resolution, particularly at DSBs, and how this activity intersects with HR still remain largely undefined.

To help address these knowledge gaps, we have employed proximity ligation coupled with mass spectrometry to identify BRCA2-DSS1 interactors that function in R-loop processing^30–32^. This effort has led to the identification of HELZ, an uncharacterized putative RNA helicase, as a BRCA2-DSS1 interactor. While HELZ has been previously linked to translational regulation and mRNA decay^33,34^, a possible role in R-loop resolution and genome maintenance has not been explored. We showed that HELZ is an ssRNA-specific R-loop resolvase essential for unwinding R-loops during transcription-coupled HR at DSBs. Furthermore, we established a critical interplay between BRCA2-DSS1 and HELZ in R-loop regulation and HR promotion, providing insights into the BRCA2 mechanisms linking R-loop homeostasis, DNA damage repair, and tumorigenesis.

## Results

### Identification of HELZ as an interactor of BRCA2-DSS1

**Fig. 1:**
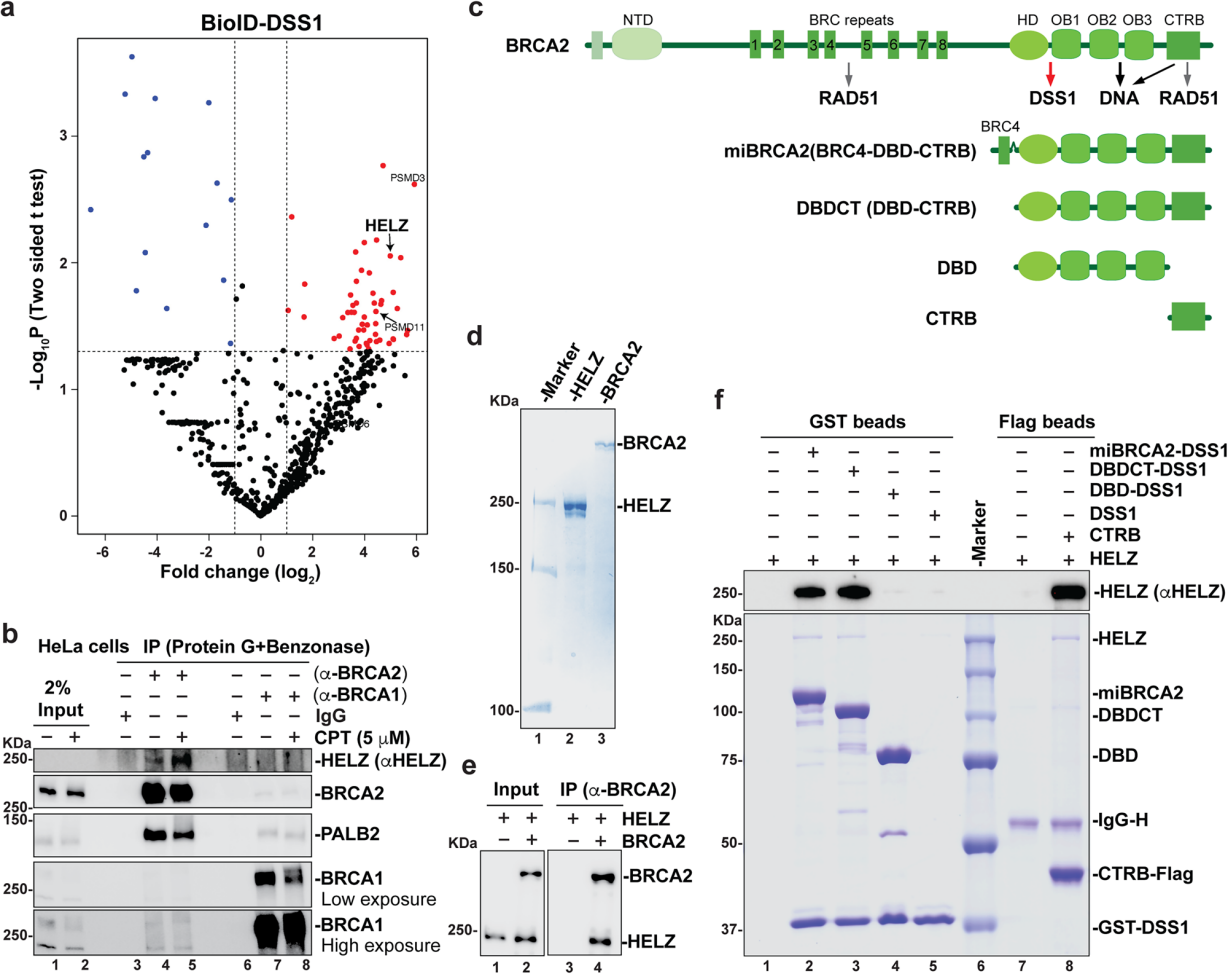
HELZ is a BRCA2–DSS1 interactor that directly binds the BRCA2 CTRB domain. **a** Volcano plots indicate HELZ as a high-ranking partner of DSS1 by BioID-LC-MS/MS. Red dots represent significantly upregulated proteins (*p* < 0.05 and log_2_FC > 1, two-sided Student’s *t*-test). Blue dots represent significantly downregulated proteins (*p* < 0.05 and log_2_FC < −1, two-sided Student’s *t*-test) Black dots represent non-significant proteins. Dashed lines illustrate the cutoffs. *x* axis, fold change at log_2_ scale; *y* axis, minus *p* value at log_10_ scale. Source data are provided as a Source Data file. **b** Immunoprecipitation (IP) assay of HELZ from HeLa cells showing that HELZ interacts with BRCA2, but not BRCA1 in the presence of Benzonase. Anti-BRCA2, HELZ, PALB2 and BRCA1 antibodies were used for probing. Source data are provided as a Source Data file. **c** Illustration of BRCA2 and various truncations such as miBRCA2 and CTRB. NTD N-terminal DNA binding domain, BRC repeat breast cancer repeat, DBD DNA binding domain, HD helical domain, OB oligonucleotide/oligosaccharide binding, CTRB C-terminal RAD51-binding. **d** SDS-PAGE analysis of purified HELZ and BRCA2 with Coomassie blue staining. Source data are provided as a Source Data file. **e** Co-immunoprecipitation assay showing direct interaction of recombinant HELZ and full-length BRCA2. Anti-BRCA2 and HELZ antibodies are used for probing. Source data are provided as a Source Data file. **f** GST pull down and flag pull down assays showing that BRCA2-CTRB provides the major interface for HELZ binding. Samples from elution fractions were analyzed by SDS-PAGE for Coomassie blue staining and western blot with anti-HELZ antibody. Source data are provided as a Source Data file.

We utilized proximity ligation coupled with mass spectrometry^30–32^ in an effort to capture proteins that stably or transiently interact with BRCA2-DSS1 (Supplementary Fig. 1a). First, we created various engineered biotin ligase (e.g., TurboID, Split-TurboID, BioID) tagged DSS1 constructs and found that only the BioID tag does not affect the homologous recombination function of DSS1 (Supplementary Fig. 1b). Then, we generated a stable HeLa-shDSS1 cell line expressing BioID-DSS1, where the endogenous DSS1 is depleted by doxycycline-induced shDSS1 expression^26^. We treated these cells with olaparib and added biotin to trigger biotinylation of potential DSS1 interaction partners, such as BRCA2 (Supplementary Fig. 1c), during the DNA damage response and repair. Finally, the biotinylated proteins were enriched using streptavidin resin and analyzed by mass spectrometry. As shown in Fig. 1a, among the top hits we identified known DSS1 interactors, such as PSMD3 and PSMD11^35^, confirming the specificity and sensitivity of our proximity proteomics approach. Notably, HELZ, a putative RNA helicase, also emerged as a highly ranked hit (Fig. 1a). To date, only two studies have characterized HELZ, implicating it in translational initiation and mRNA decay^33,34^.

### HELZ physically interacts with the C-terminal region of BRCA2

To determine if HELZ directly interacts with DSS1 or BRCA2-DSS1 in cells, we performed co-immunoprecipitation (Co-IP) experiments with benzonase treatment of cell lysate to eliminate RNA and DNA. As shown in Supplementary Fig. 1d, Flag-GFP-DSS1 ectopically expressed in 293T cells co-precipitated HELZ along with components of the BRCA2-PALB2-RAD51 complex. Notably, overexpression of MBP-BRCA2 markedly enhanced the amount of co-precipitating HELZ, suggesting a potential physical association between HELZ and BRCA2. Consistently, we found that ectopically expressed HELZ can interact with either exogenous or endogenous BRCA2 (Supplementary Fig. 1e, f), and the interaction between endogenous BRCA2 and HELZ can be enhanced by treatment of cells with the DNA topoisomerase I inhibitor camptothecin (CPT) treatment (Fig. 1b). Of note, little or no interaction of HELZ with BRCA1 was seen in co-IP analysis, which highlights the specificity of HELZ for BRCA2 (Fig. 1b, and Supplementary Fig. 1f).

To ask whether HELZ and BRCA2-DSS1 physically interact, we expressed HELZ, BRCA2 and DSS1 in insect cells, purified them to near homogeneity, and performed co-IP and pull-down assays in vitro (Fig. 1c, d, and Supplementary Fig. 1g). As shown in Fig. 1e, f, we found that HELZ interacts with BRCA2 and has little or no affinity for DSS1. To identify the portion of BRCA2 involved in complex formation with HELZ, we used affinity pulldown to test different segments of the BRCA2 protein for the ability to associate with HELZ (Fig. 1c, and Supplementary Fig. 1g). The results, as shown in Fig. 1f, revealed that HELZ engages BRCA2 through the C-terminal Recombinase Binding (CTRB) region of BRCA2 that is encoded by gene exon 27 and critical for tumor suppression function of BRCA2^15^.

### HELZ binds ssRNA and R-loops in vitro

**Fig. 2:**
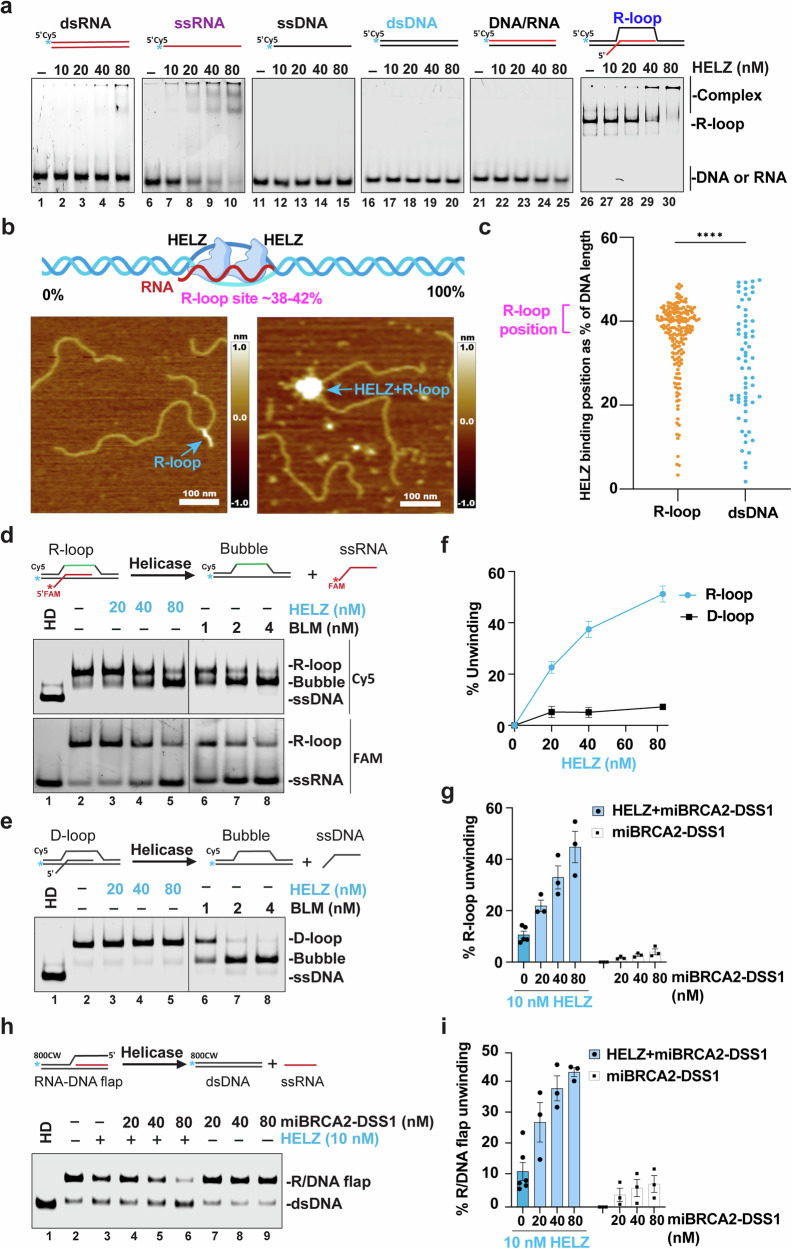
RNA-specific helicase activity of HELZ and its stimulation by BRCA2. **a** Binding of dsRNA, ssRNA, ssDNA, dsDNA, DNA/RNA hybrid, and R-loop by HELZ as examined by EMSA. The representative image of three independent experiments was provided. Source data are provided as a Source Data file. **b** Schematic of R-loop bound by HELZ (Created in BioRender. Zhao, W. (2026) https://BioRender.com/zobez1x) (top). Representative AFM topography images (bottom) of linear, transcribed 2795 bp pFC-*Arin* fragment containing an R-loop, incubated with purified HELZ (Cyan arrows depict R-loops or HELZ bound R-loops). scale bar=100 nm. When incubated with R-loop DNA, HELZ binding events, which displayed AFM heights (4.34 ± 1.82 nm) significantly greater (*p* = 1.49357E-43) than R-loop alone (1.15 ± 0.16 nm), were enriched at 34–42% from the closest DNA end, corresponding to the R-loop region. **c** Position distribution of HELZ on either a linear, transcribed 2795 bp pFC-*Arin* fragment containing an R-loop or control dsDNA was measured as its distance to the nearest DNA end along the linear DNA. The distributions of HELZ on R-loop DNA and control dsDNA were compared using a two-sample two-sided Student’s *t*-test (****p = 1.00829E-05). *n* = 100 and 186 events for the control and R-loop DNA conditions, respectively. Source data are provided as a Source Data file. **d** Schematic of the R-loop unwinding assay with ssRNA labeled with FAM and bubble DNA labeled with Cy5 (top). Native PAGE gel showing representative R-loop unwinding by HELZ (20, 40, and 80 nM; lanes 3–5) and BLM (1, 2, and 4 nM; lanes 6–8) (bottom) at 15 min. HD: Heat-Denatured. Source data are provided as a Source Data file. **e** Schematic of the D-loop unwinding assay with bubble DNA labeled with Cy5 (top). Native PAGE gel showing representative D-loop unwinding by HELZ (20, 40, and 80 nM; lanes 3–5) and BLM (1, 2, and 4 nM; lanes 6–8) (bottom). Source data are provided as a Source Data file. **f** Quantification of the R-loop and D-loop unwinding experiments by HELZ in **d** and **e**. The data represent the mean ± SEM of *n* = 5 (HELZ with R-loop), *n* = 4 (BLM with R-loop) or *n* = 2 (HELZ or BLM with D-loop) independent experiments. Source data are provided as a Source Data file. **g** Quantification of the unwinding assay in Supplementary Fig. 4c to indicate the stimulation of miBRCA2-DSS1 on R-loop unwinding by HELZ. The data represent the mean ± SEM of three independent experiments. Source data are provided as a Source Data file. **h** Schematic of the RNA-DNA flap unwinding assay with one strand of DNA labeled with 800CW (top). Native PAGE gel showing representative RNA-DNA flap unwinding by HELZ (10 nM; lane 3), its stimulation by miBRCA2-DSS1 (20, 40, and 80 nM; lanes 4–6), and by miBRCA2-DSS1 alone (20, 40, and 80 nM; lanes 7–9) at 15 min (bottom). Source data are provided as a Source Data file. **i** Quantification of the unwinding assay in **g**. The data represent the mean ± SEM of three independent experiments. Source data are provided as a Source Data file.

Since HELZ is predicted as a putative RNA helicase and has been reported to play a role in RNA metabolism^33,34^, we first used the DNA electrophoretic mobility shift assay (EMSA) to test the ability of purified full-length HELZ (1942 amino acid residues, 219 kDa, Fig. 1d) to bind various RNA substrates, including single-stranded RNA (ssRNA), double-stranded RNA (dsRNA), RNA-DNA hybrids (with no overhang, 3′-RNA overhang or 5′-RNA overhang), R-loop, and also various DNA species, namely, single-stranded DNA (ssDNA), double-stranded DNA (dsDNA), the D-loop and DNA bubble. The results revealed that HELZ has the highest binding affinity for ssRNA and R-loop, followed by RNA-DNA hybrid with ssRNA overhang (either 5′ or 3′), DNA bubble, dsRNA, D-loop, RNA-DNA hybrid with no overhang, dsDNA and ssDNA (Fig. 2a, and Supplementary Fig. 2a, b). Also, we noticed that the minimal ssRNA length required for HELZ binding is 15 nt (Supplementary Fig. 2c, d). Then, we performed atomic force microscopy (AFM) imaging to directly visualize HELZ and its binding to R-loops. R-loop structures were generated at a defined region approximately 38–42% from the nearest DNA end in the linearized pFC53 plasmid, as previously described^36,37^. Upon incubation with R-loop DNA, HELZ binding was enriched at the R-loop region (Fig. 2b, c). In contrast, HELZ binding to control dsDNA was randomly distributed along the molecule (Fig. 2b, c, and Supplementary Fig. 3a). Thus, results from our AFM imaging and EMSA analyzes (Fig. 2b, and Supplementary Fig. 2) provide strong evidence that HELZ specifically recognizes R-loops. Furthermore, by applying an established method for calculating the molecular size of proteins in AFM analyzes^38^, we determined that HELZ primarily exists as a monomer in the absence of DNA (Supplementary Fig. 3b), which is further confirmed by mass photometry analysis (Supplementary Fig. 3c). Interestingly, HELZ appeared as larger oligomers when bound to R-loops (Fig. 2b, c, and Supplementary Fig. 3d), suggesting stable R-loop engagement is mediated by multiple HELZ protomers.

### HELZ is an ssRNA-specific helicase to unwind R-loops in a BRCA2-stimulated manner

To interrogate the RNA helicase activity of HELZ in vitro, we first used RNA-DNA hybrid substrates with 3′-, 5′- or no RNA overhang. HELZ preferentially unwound the substrate with a 5′-RNA overhang (Supplementary Fig. 4a), indicating that the RNA helicase activity of HELZ has a 5′−3′ polarity with regards to the bound ssRNA strand. Next, we tested R-loop substrates, comprising an RNA-DNA hybrid with 5′-RNA overhang and the associated non-template ssDNA. HELZ unwound these R-loops in a protein concentration- and time-dependent manner, though less efficiently than the control BLM helicase (Fig. 2d, and Supplementary Fig. 4b). In contrast, HELZ, unlike BLM^39^, could not unwind D-loop substrates (Fig. 2e, f). Importantly, we observed that HELZ-mediated R-loop unwinding is enhanced more than threefold by miBRCA2 (BRC4-DBDCT)-DSS1 and DBDCT-DSS1, but not by BRCA1-BARD1, DBD-DSS1, or CTRB alone (Fig. 2g, and Supplementary Figs. 4c, d and 5). These results suggest that both formation of the BRCA2-HELZ complex via CTRB and the presence of the BRCA2 DNA-binding domain (DBD) and its partner DSS1, likely due to the robust DNA-binding activity of DBD and its ssDNA binding modulation by DSS1, are required for stimulation of HELZ activity. Furthermore, we tested HELZ on a 5′ RNA-DNA flap structure designed to mimic a physiological branch-migratable R-loop^40^. As shown in Fig. 2h, i and Supplementary Fig. 4e, f, HELZ could dissociate the flap structure to yield a dsDNA product, and this HELZ activity was also stimulated by miBRCA2-DSS1 but not by BRCA1-BARD1. Collectively, these findings indicate that HELZ is an ssRNA-specific helicase and the HELZ-BRCA2 complex can effectively resolve R-loops in vitro.

### HELZ is recruited to R-loops, and this recruitment is facilitated by BRCA2 in cells

**Fig. 3:**
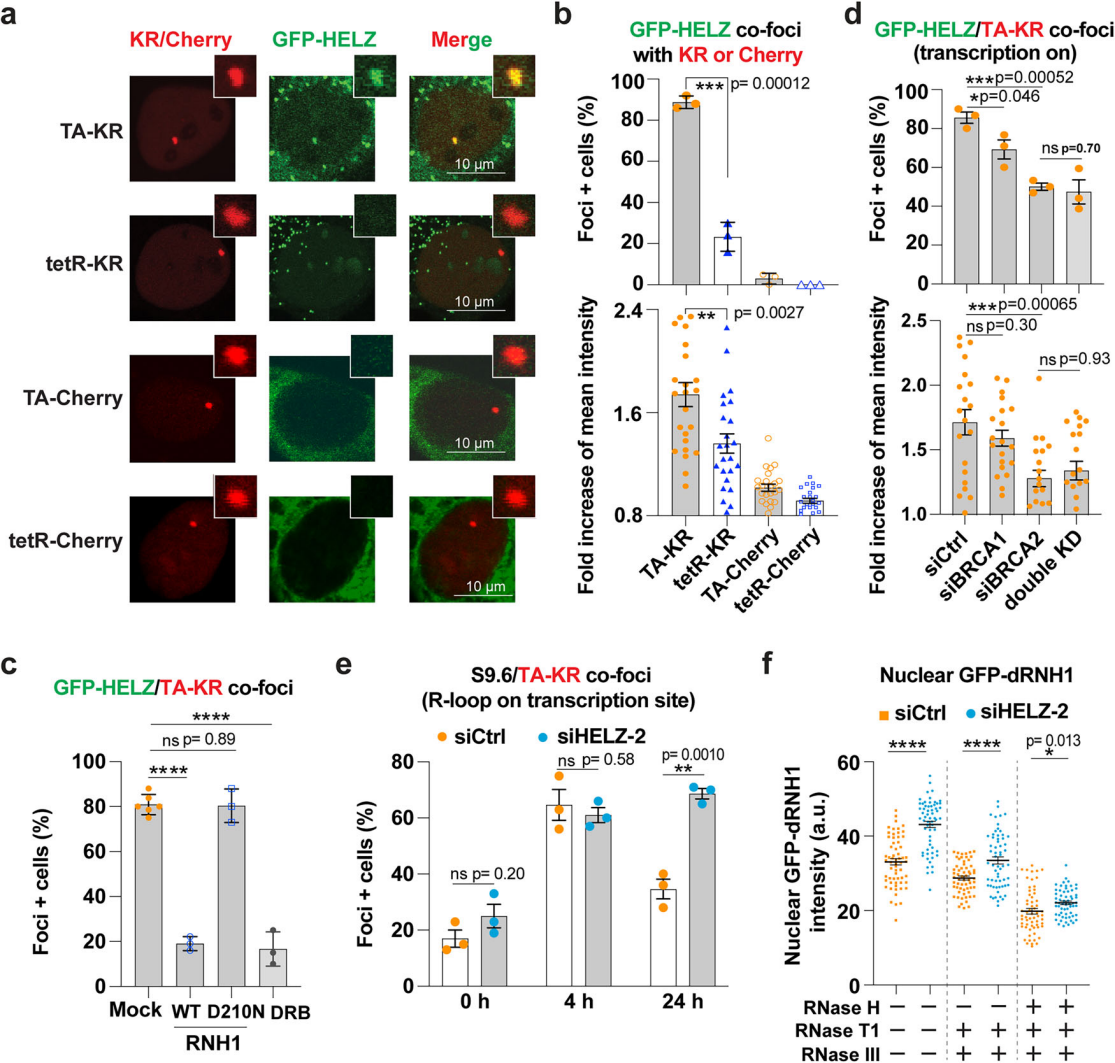
HELZ is recruited to and dissociates R-loops in cells. **a** U2OS-TRE cells transfected with pBROAD3 TetR-KR/Cherry or pBROAD3 TA-KR/Cherry and the expression vector of GFP-HELZ were light-activated and recovered 20 min before fixation. Representative images of GFP foci recruitment at sites of TA-KR, TetR-KR, TA-Cherry or TetR-Cherry were shown. Scale bar: 10 μm. **b** Foci-positive cells in each indicated group were quantified. The mean values ± SD of three independent experiments are shown (top). Fold increase of HELZ foci intensity of TA-KR, TetR-KR, TA-Cherry and TetR-Cherry compared to background was quantified in 24 cells (mean ± SEM; bottom). ns, not significant; ^**^P ≤ 0.01; ^****^P ≤ 0.0001 (two-sided Student’s *t*-test, 1.36178E-07 for Mock vs RNH1^WT^, 8.00554E-07 for Mock vs DRB). Source data are provided as a Source Data file. **c** Foci-positive cells with HELZ colocalization with TA-KR upon treatment with DRB and overexpression of RNase H1^WT^ or RNaseH1^D210N^ were quantified. The mean values ± SEM of three independent experiments are shown. ns, not significant; ^****^P ≤ 0.0001 (two-sided Student’s *t*-test). Source data are provided as a Source Data file. **d** Foci-positive cells with HELZ colocalization with TA-KR (top). The mean values ± SEM of three independent experiments are shown. Fold increase of HELZ foci intensity upon treatment with siBRCA1 or siBRCA2 or the combination were quantified in 20 cells (mean ± SEM) (bottom). ns, not significant (two-sided Student’s *t*-test); ^*^P ≤ 0.05; ^***^P ≤ 0.001. Source data are provided as a Source Data file. **e** Foci-positive cells with S9.6 colocalization with TA-KR upon treatment with siHELZ-2 were quantified. The mean values ± SEM of three independent experiments is shown. ns, not significant (two-sided Student *t* test); ^**^P ≤ 0.01. Source data are provided as a Source Data file. **f** Quantification of mean nuclear GFP-dRNH1 intensity from three independent experiments. Scatter plots show 61 nuclei per condition. Error bars represent the standard error of the mean (SEM). ^*^P < 0.05 and ^****^P < 0.0001 (two-sided Mann–Whitney U test, 2.28925E-13 and 3.65785E-05 for siCtrl vs siHELZ-2 upon no or RNase (T1 + III) treatment). Source data are provided as a Source Data file.

We employed the DNA damage at RNA-transcribed sites (DART) system to examine the recruitment of GFP-HELZ to R-loops in real-time within live cells^41,42^. In this system, a tandem tetracycline-responsive element (TRE) array cassette with an adjacent reporter gene was stably integrated into the U2OS cell genome. KillerRed (KR), a light-activatable chromophore, is fused to either TetR alone (TetR-KR) or TetR together with the transcriptional activator VP16 (TA-KR), enabling localized oxidative DNA damage and strand breaks at the TRE locus through light-induced production of reactive oxygen species. Both fusion proteins can bind to the TRE and target KR to the locus, but only TA-KR is able to locally activate reporter gene transcription. Together, TA-KR can robustly induce R-loop formation at the TRE locus, while TA-Cherry, TetR-KR, or TetR-Cherry are unable to do so^41,42^. By confocal microscopy, we observed that GFP-HELZ preferentially colocalizes with TA-KR (Fig. 3a, b, and Supplementary Fig. 6a). In addition, HELZ recruitment to TA-KR was significantly diminished by the expression of wild type but not the D210N catalytic dead mutant of RNase H1, or by the treatment cells with the transcription inhibitor DRB (Fig. 3c). These results provide direct evidence for the recruitment of HELZ to R-loops enriched at DNA damage sites in cells.

Importantly, we found that HELZ and BRCA2 both colocalize with TA-KR in the nucleus, and that HELZ recruitment to TA-KR is significantly reduced after BRCA2 depletion, whereas BRCA1 knockdown has only a minor effect (Fig. 3d, and Supplementary Fig. 6a–c). Notably, HELZ recruitment is decreased but not abolished after BRCA2 loss, indicating that BRCA2 facilitates, but is not strictly required for, HELZ localization to R-loop-enriched regions. Consistent with this, in situ proximity ligation assays (PLA) revealed BRCA2-HELZ colocalization, with PLA signals significantly increasing following CPT treatment (Supplementary Fig. 6d). Furthermore, the co-foci of TA-KR with the R-loop specific monoclonal antibody and the signal of S9.6 slot blot were significantly higher upon siHELZ treatment in cells, indicating that R-loop levels at the TA-KR site are elevated upon HELZ depletion (Fig. 3e, and Supplementary Fig. 6e–j). We also assessed nuclear R-loop accumulation using a catalytically inactive GFP-dRNase H1 (dRNH1) probe^43^. As shown in Fig. 3f, and Supplementary Fig. 6k, HELZ knockdown resulted in a significant increase in whole nuclear GFP-dRNH1 signal following treatment with either mock or RNase T1 + RNase III, which selectively degrade ss- and ds-RNA while preserving RNA-DNA hybrids. Pretreatment with a combination of RNase H, RNase T1, and RNase III significantly suppressed GFP-dRNH1 intensity in siCtrl as well as siHELZ-treated samples to background levels, further confirming nuclear R-loop accumulation upon HELZ depletion. Importantly, ectopic expression of wild-type HELZ (HELZ^WT^) but not the HELZ catalytically inactive mutant (HELZ^K674N^) could prevent R-loop accumulation in cells depleted of endogenous HELZ (Supplementary Fig. 6h–j; see below). Together, the results demonstrate that HELZ is recruited to R-loops and required for global R-loop resolution in cells.

### HELZ drives transcription-coupled HR during DNA damage response

**Fig. 4:**
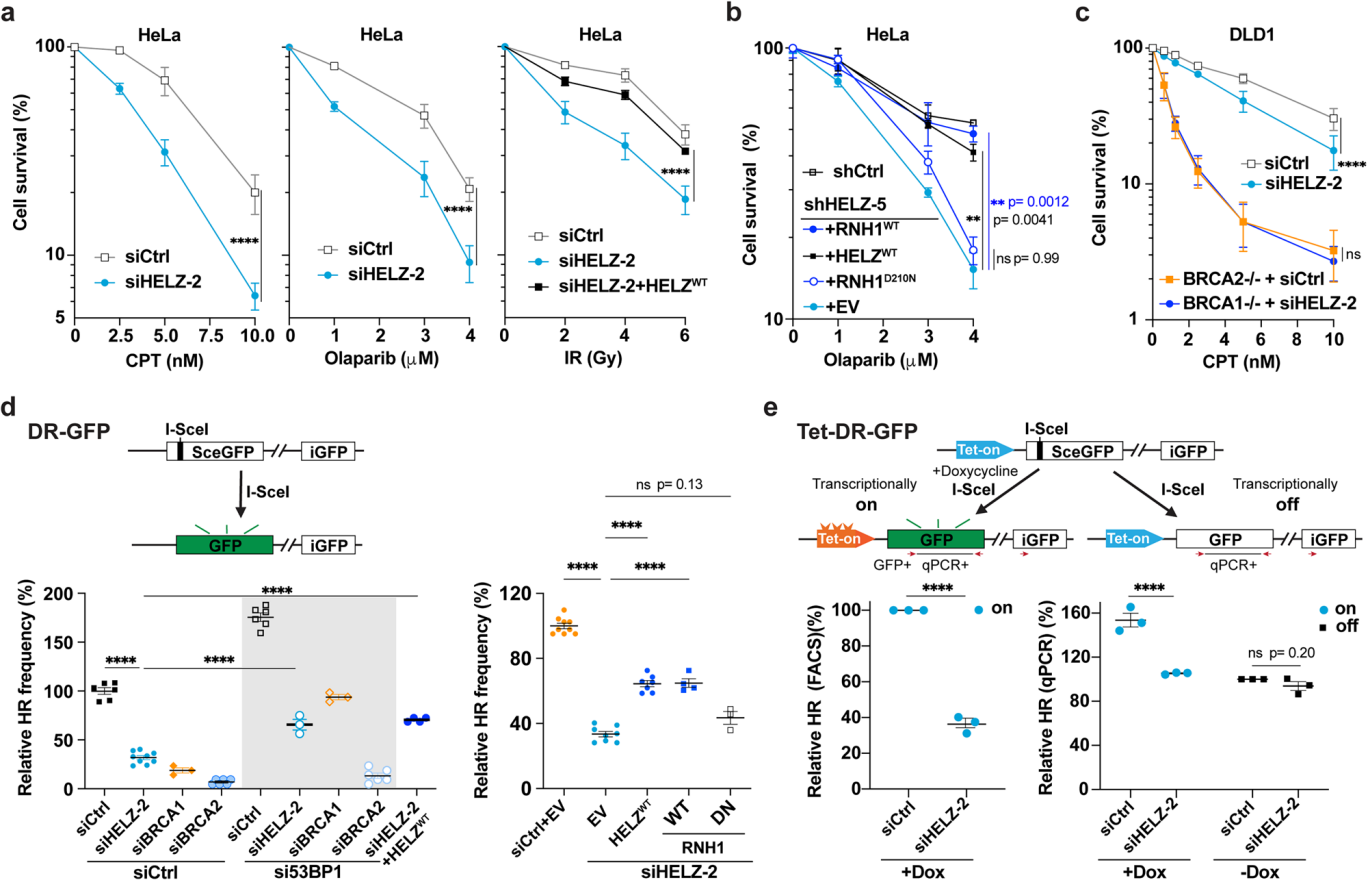
HELZ is required for cell survival and HR, particularly in transcriptionally active genomic regions. **a** Clonogenic survival of HeLa cells depleted with HELZ by siHELZ-2 upon treatment with CPT, olaparib, or IR. Error bars, SEM (*n* = 3 independent experiments). Symbol: EV, empty vector; HELZ^WT^, with ectopic expression of wild type HA-HELZ. ^****^P ≤ 0.0001 (two-way ANOVA). Source data are provided as a Source Data file. **b** Clonogenic survival of HeLa cells depleted with shHELZ-5 and ectopic expression of HELZ or RNase H1 WT (RNH1^WT^) or RNase H1 D210N (RNH1^D210N^) upon treatment with olaparib, Error bars, SEM (*n* = 3 independent experiments). Symbol: EV, empty vector. ns, not significant; ^**^P ≤ 0.01 (two-way ANOVA). Source data are provided as a Source Data file. **c** Clonogenic survival of DLD1 or DLD1 (BRCA2^−/−^) cells depleted with HELZ by siHELZ-2 upon treatment with CPT. Error bars, SEM (*n* = 4 for DLD1 and *n* = 2 for DLD1 (BRCA2^−/−^) independent experiments). ns, not significant, ^****^P ≤ 0.0001 (two-way ANOVA). Source data are provided as a Source Data file. **d** Schematic of HR assay using the DR-GFP reporter (top). Quantification of HR assay results from DR-U2OS cells upon transfection with siRNA against HELZ, BRCA1, BRCA2, and control (siCtrl) without or with si53BP1 (bottom left). Quantification of HR assay results from DR-U2OS cells upon transfection with siHELZ-2 along with transient expressing HELZ or RNase H1 (wild type or D210N mutant) (right). Error bars, SEM (*n* = 3, 4, 6, 7, 8, or 9 repeats of three independent experiments). Symbol: EV, empty vector. ns, not significant, ^****^P ≤ 0.0001(one-way ANOVA). Source data are provided as a Source Data file. **e** Schematic of HR assay using the Tet-DR-GFP reporter (top). Quantification of HR assay results from Tet-DR-GFP U2OS cells upon transfection of siRNA against HELZ (siHELZ-2). Error bars, SEM (*n* = 3 independent experiments). ns, not significant, ^****^P ≤ 0.0001 (one-way ANOVA). Source data are provided as a Source Data file.

To explore the biological consequences of HELZ dysfunction, we first assessed cell survival upon treatment of siHELZ in HeLa cells with CPT, olaparib, and ionizing radiation (IR) (Fig. 4a). The results showed that HELZ is needed for cell survival upon the occurrence of DNA damage. Importantly, the DNA damage hypersensitivity of HELZ-depleted cells could be corrected by the expression of HELZ or RNase H1 but not by the nuclease-dead mutant RNase H1^D210N^ (Fig. 4a, b, and Supplementary Fig. 7a, b). We also used the BRCA2-null DLD1 cancer cell line to investigate the epistatic relationship between BRCA2 and HELZ. Notably, HELZ depletion sensitized BRCA2-proficient DLD1 parental cells to CPT, but did not further enhance the sensitivity of BRCA2-deficient DLD1 cells (Fig. 4c, and Supplementary Fig. 7c), supporting an epistatic interaction between HELZ and BRCA2. Then, we employed the DR-GFP reporter assay to evaluate how HELZ might impact HR repair^44,45^. As shown in Fig. 4d, HELZ knockdown significantly reduced HR repair efficiency. Interestingly, knockdown of 53BP1, a negative regulator of DNA end resection^46^, restored HR proficiency in HELZ depleted cells (Fig. 4d, and Supplementary Fig. 7d). Consistent with the cell survival data (Fig. 4a, b, and Supplementary Fig. 7a, b), the HR defect in HELZ depleted cells could be overcome by HELZ or RNase H1 expression, but RNase H1^D210N^ was ineffective in this regard (Fig. 4d). These results highlight the role of HELZ in resolving R-loops at DSBs to help promote DNA end resection of HR, and also suggest that HELZ is required for resolving R-loops during transcription-coupled HR (TC-HR). So, we utilized the Tet-DR-GFP reporter assay^47^ designed to assess HR repair efficiency in the presence (On) or absence (Off) of transcription. GFP expression, controlled by a tetracycline-inducible promoter, was measured as TC-HR by fluorescence-activated cell sorting (FACS) following induction of an I-*Sce*I-mediated DSB within the GFP gene, with transcription initiated by DOX treatment. We found that TC-HR activity is attenuated by HELZ depletion when transcription was on (Fig. 4e). To evaluate HR independently of GFP transcription, we further quantified repair products using specific primers and quantitative PCR (qPCR) (Fig. 4e), and found that HR repair efficiency is increased by 1.5-fold upon DOX treatment, consistent with previous observations^47,48^. Importantly, HELZ knockdown reduced TC-HR when co-transcription was active, while having little or no effect when transcription was off (Fig. 4e, and Supplementary Fig. 7e). These results highlight the essential role of HELZ in the DNA damage response and efficient DSB repair by HR, particularly in transcription-coupled HR.

### Role of HELZ helicase activity in R-loop resolution and HR repair

**Fig. 5:**
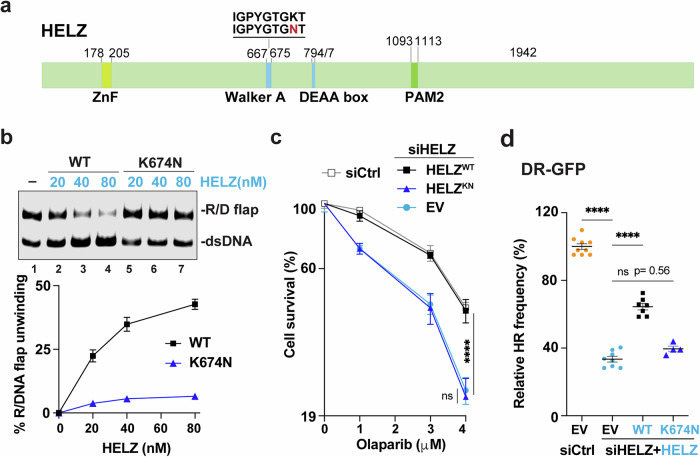
HELZ helicase activity is required for R-loop resolution and HR. **a** Schematic of HELZ with various functional domains, highlighting the K674 residue. **b** Native PAGE gel shows representative RNA-DNA flap unwinding by HELZ^WT^ or its mutant form HELZ^K674N^ (top). Quantification is shown below; the data represent the mean ± SEM of three independent experiments. Source data are provided as a Source Data File. **c** Clonogenic survival of HeLa cells depleted with HELZ and ectopic expression of empty vector (EV), HELZ^WT^ or HELZ^K674N^ upon treatment with Olaparib. ^****^P ≤ 0.0001 (*n* = 3 independent experiments; mean ± SEM; two-way ANOVA). Source data are provided as a Source Data File. **d** Quantification of HR assay results from DR-U2OS cells upon treatment of siHELZ-2 and ectopic expression of empty vector (EV; *n* = 8 repeats of at least three independent experiments), HELZ^WT^ (*n* = 7 repeats of at least three independent experiments), or HELZ^K674N^ (*n* = 4 repeats of at least three independent experiments), in comparison with siCtrl + EV (*n* = 9  repeats of at least three independent experiments). ns, not significant; ^****^P ≤ 0.0001 (mean ± SEM; one-way ANOVA). Source data are provided as a Source Data File.

Having demonstrated that HELZ is capable of unwinding R-loops in vitro and reducing R-loop levels to promote HR in cells, we investigated whether its helicase activity is essential for R-loop resolution and suppression of R-loop accumulation in HELZ-depleted cells. To this end, we mutated the conserved lysine residue (Lys674) in the Walker A motif of HELZ to asparagine, purified the HELZ^K674N^ mutant protein, and examined its helicase activity (Fig. 5a). As shown in Fig. 5b and Supplementary Fig. 8a–c, HELZ^K674N^ failed to unwind R-loops, despite exhibiting comparable R-loop binding affinity to wild-type HELZ. Notably, cells expressing HELZ^K674N^ displayed a higher level of R-loops compared to HELZ^WT^ cells (Supplementary Fig. 6h–j), and HELZ^K674N^ was unable to restore HR activity or rescue cell survival following DNA damage in HELZ-depleted cells (Fig. 5c, d, and Supplementary Fig. 8d, e). These findings demonstrate that the helicase activity of HELZ is essential for resolving R-loops, preventing their accumulation even without exogenous DNA damage, facilitating HR repair, and supporting cell viability in response to DNA damage.

### R-loop resolution of HELZ promotes DNA end resection in HR

**Fig. 6:**
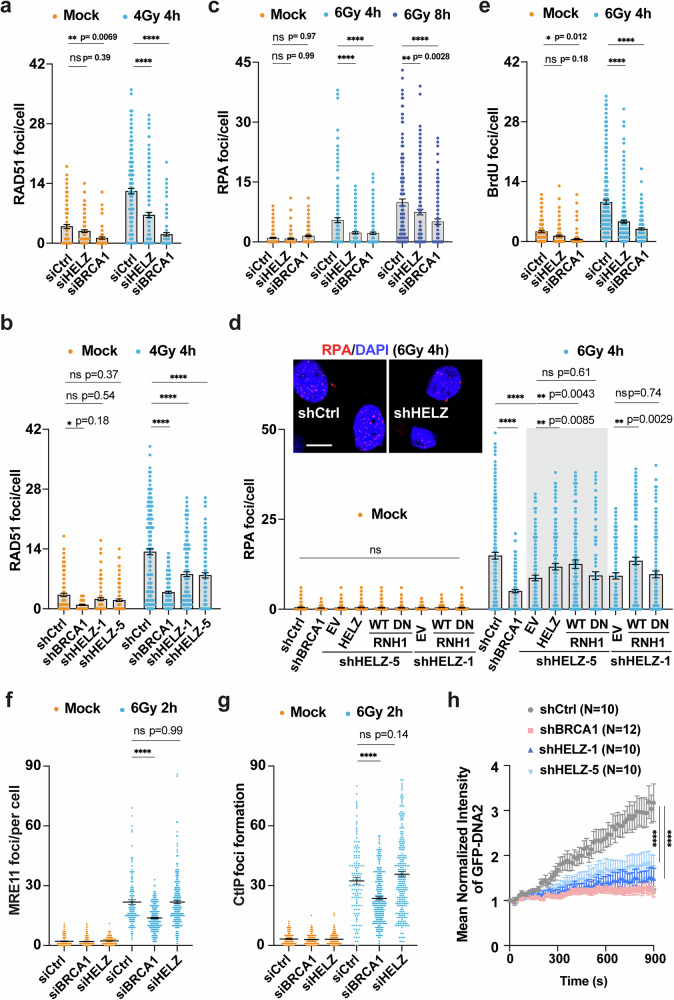
HELZ promotes DNA end resection in a manner dependent on R-loop resolution. **a** Quantification of RAD51 foci per cell 4 h after exposure to 4 Gy irradiation in HeLa cells upon siBRCA1 or siHELZ-2. Source data are provided as a Source Data File. **b** Quantification of RAD51 foci per cell 4 h after exposure to 4 Gy irradiation in HeLa cells upon shBRCA1 or shHELZ-2. Source data are provided as a Source Data File. **c** Quantification of RPA foci per cell 4 or 8 h after exposure to 6 Gy irradiation in HeLa cells upon siBRCA1 or siHELZ-2. Source data are provided as a Source Data File. **d** Quantification of RPA foci per cell 4 h after exposure to 6 Gy irradiation in HeLa cells upon shBRCA1 or shHELZ together with the transient ectopic expression of HELZ (WT or K674N) or RNase H1 (WT or D210N). Source data are provided as a Source Data File. **e** Quantification of BrdU foci per cell 4 h after exposure to 6 Gy irradiation in HeLa cells upon siBRCA1 or siHELZ-2. Source data are provided as a Source Data File. **f** Quantification of MRE11 foci per cell 2 h after exposure to 6 Gy irradiation in HeLa cells upon siBRCA1 or siHELZ. Source data are provided as a Source Data File. **g** Quantification of CtIP foci per cell 2 h after exposure to 6 Gy irradiation in HeLa cells upon siBRCA1 or siHELZ. Source data are provided as a Source Data File. **a**–**g** Mean values ± SEM of 100 cells from one representative experiment were counted. Data are representative of *n* = 3 independent experiments with similar results. ns, not significant; ^*^P ≤ 0.05; ^**^P ≤ 0.01; ^****^P ≤ 0.0001 (two-way ANOVA). **h** Quantification of GFP-DNA2 recruitment over time after laser micro irradiation in HeLa cells upon shCtrl, shBRCA1 or shHELZ from three independent experiments. Data show the mean values ± SEM of *n* cells analyzed, where *n* = 10 (for shCtrl and shHELZ) or 12 (for shBRCA1). ^****^P ≤ 0.0001 (two-way ANOVA). Source data are provided as a Source Data File.

We next interrogated a possible role of HELZ in DNA end resection during DSB repair by HR. First, we found that knockdown of HELZ using siRNA or shRNA significantly reduces IR-induced RAD51 foci formation (Fig. 6a, b, and Supplementary Figs. 9a, b and 10a–c). Then, we examined IR-induced formation of Replication Protein A (RPA) foci, a marker of resected DNA ends. HELZ depletion led to a marked reduction in RPA foci at 4 h post-IR, which became less pronounced by 8 h (Fig. 6c, d, and Supplementary Fig. 9c). Importantly, the defect in assembling RPA foci upon IR caused by HELZ depletion was largely complemented by the ectopic expression of wild-type HELZ or RNase H1 but not by RNase H1^D210N^ (Fig. 6d, and Supplementary Fig. 9d). These findings highlight that HELZ facilitates the timely resolution of R-loops for successful DNA resection. To directly assess DNA end resection, we labeled resection tracks in genomic DNA using 5-bromo-2ʹ-deoxyuridine (BrdU) and detected ssDNA/BrdU foci under non-denaturing conditions. We also performed a qPCR-based resection assay at a site-specific DSB in DIvA (AsiSI-ER-U2OS) cells^49,50^. Compared with control cells, HELZ-depleted cells exhibited significantly fewer ssDNA/BrdU foci following IR and reduced qPCR signal following 4-OHT treatment (Fig. 6e, and Supplementary Figs. 9e and 10d–g), thus confirming a critical role of HELZ in promoting DNA end resection. Consistent with this, HELZ-depleted cells showed reduced pATR and pChk1 signals, accompanied by increased pATM and γH2AX signals (Supplementary Fig. 10h). Furthermore, we found that HELZ knockdown has no impact on foci of MRE11 and CtIP that function in short range end resection (Fig. 6f, g, and Supplementary Fig. 11a–c), but significantly impaired the recruitment of DNA2 needed for long-range resection downstream of MRE11/CtIP in the laser microirradiation experiments (Fig. 6h, and Supplementary Fig. 11d). These findings suggest that unresolved RNA-DNA hybrids at DSBs may directly or indirectly obstruct enzymes involved in long-range resection^2,51^, consistent with a previous report showing that such hybrids inhibit EXO1- and BLM-DNA2-mediated resection in vitro^52^. Together, these results identify a role for HELZ as a critical factor in the successful execution of DNA end resection, likely through its function in resolving R-loops.

### HELZ is essential for the survival of R-loop-enriched cancer cells

**Fig. 7:**
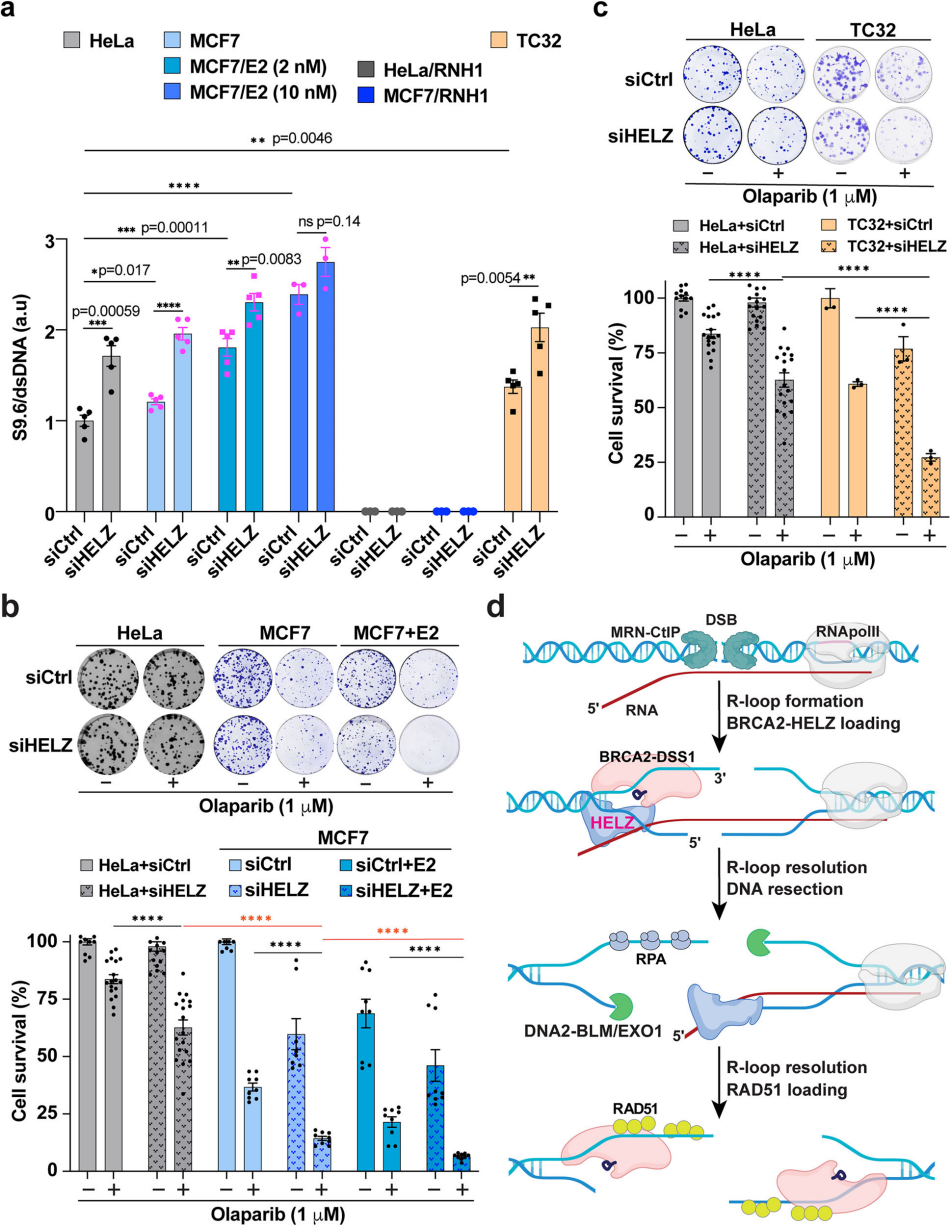
HELZ becomes essential for the survival of R-loop-enriched cancer cells. **a** Quantification (mean ± SEM) of enrichment of R-loop (detected by S9.6 antibody) normalized by dsDNA into genomic DNA of d. au: arbitrary unit. Statistical analysis was done from three independent experiments with the two-sided Student’s *t*-test, ^*^p ≤ 0.05, ^**^p ≤ 0.01, ^***^p ≤ 0.001, ^****^p = 1.83873E-05 ≤ 0.0001. Source data are provided as a Source Data File. **b** Representative images (top) and quantification (bottom) of clonogenic survival in HeLa and MCF7 cells, with and without E2 exposure, following siHELZ-2 and olaparib treatment. The data represent the mean ± SEM of six (HeLa) and three (MCF7) independent experiments. ^****^P ≤ 0.0001 (*n* = 3 technical replicates per experiment; two-way ANOVA). Source data are provided as a Source Data File. **c** Representative images (top) and quantification (bottom) of clonogenic survival in HeLa and TC32 cells upon siHELZ and olaparib treatment. For HeLa cells, the data represent the mean ± SEM of six (*n* = 3 technical replicates per experiment) independent experiments. For TC32 cells, the data represent the mean ± SEM of one representative experiment with three technical replicates from *n* = 3 independent experiments with similar results. ^**^p ≤ 0.01, ^****^p ≤ 0.0001 (*n* = 3; mean ± SEM; two-way ANOVA). Source data are provided as a Source Data File. **d** Model for HELZ functions in R-loop resolution and DNA end resection during HR at transcription active sites (Created in BioRender. Zhao, W. (2026) https://BioRender.com/owadb60). HELZ-BRCA2 is recruited to R-loops formed at transcription active sites near DSBs, possibly after the recruitment of MRN and CtIP, and resolves R-loops to promote DNA end resection, partially through the recruitment of DNA2 or other factors.

Given the critical role of HELZ in resolving R-loops for efficient HR repair, we investigated whether HELZ is upregulated in cancers to manage elevated R-loop stress and support cell survival. First, we queried the cBioPortal cancer genomic database based on TCGA PanCancer Atlas and found that *HELZ* is often amplified in certain cancers, such as breast invasive carcinoma, sarcoma and mesothelioma (Supplementary Fig. 12a). Consistently, Western blot analysis showed that the HELZ protein levels are markedly elevated in multiple breast cancer cells (especially MCF7) and Ewing sarcoma cells (e.g., TC32), compared with normal breast epithelial cells (MCF10A), fibroblasts (BJ and HSF), RPE (retinal pigment epithelium) cells, and even HELZ-non-amplified cancer cells (e.g., HeLa) (Supplementary Fig. 12a, b). Notably, the activated E2-ER (estrogen-estrogen receptor) signaling pathway in ER⁺ breast cancer^53^, as well as oncogenic EWS-FLI1 expression in TC32 cells^54^, have been reported to enhance gene transcription, thereby promoting elevated levels of co-transcriptional R-loop formation and DSBs^53^. Indeed, by slot blot assays, we confirmed that R-loop levels are elevated in MCF7 and TC32 cells, and that E2 treatment further increased R-loop accumulation and also caused cell death in MCF7 cells (Fig. 7a, and Supplementary Fig. 12c-e). Importantly, HELZ knockdown rendered MCF7 and TC32 cells much more sensitive to olaparib compared to HeLa cells (Fig. 7b, c). Additionally, E2 treatment further hypersensitized HELZ-depleted MCF7 cells to olaparib (Fig. 7b). These findings underscore the essential role of HELZ in mitigating R-loop stress, maintaining genome stability, and supporting the survival of cancer cells with elevated R-loop levels.

## Discussion

Our study identifies HELZ as an ssRNA-specific helicase and interaction partner of BRCA2 that fulfills critical roles in resolving R-loops and promoting transcription-coupled HR repair. Specifically, BRCA2-DSS1 facilitates HELZ recruitment to R-loops and promotes the resolution of these nucleic acid intermediates. By resolving R-loops and RNA-DNA hybrids, HELZ helps ensure efficient DNA end resection to maintain genome stability (Fig. 7d). Importantly, our findings also uncover a previously unrecognized role of BRCA2-DSS1 in R-loop regulation and position HELZ as a promising therapeutic target in cancers that are characterized by high levels of R-loops.

### HELZ helicase activity and its role in R-loop resolution

HELZ has remained largely uncharacterized in nuclear pathways, and our work defines its biochemical and functional properties in this context. By purifying full-length HELZ, we show that it binds ssRNA and R-loops with high affinity and unwinds these substrates in a 5′−3′ direction. Unlike helicases such as BLM, SETX, DHX9, and DDX5, which remodel a broad spectrum of nucleic-acid structures including D-loops and G-quadruplexes^28,55–61^, HELZ displays marked selectivity for ssRNA-containing hybrids and R-loops, establishing it as an ssRNA-directed R-loop helicase rather than a general nucleic-acid remodeler. Consistent with this specificity, HELZ depletion causes global and DSB-associated R-loop accumulation, in agreement with DRIP-seq data from HELZ-depleted cells reported by the Yu group^62^. Similar accumulation upon SETX or DDX5 depletion underscores that these helicases, including HELZ, are required for R-loop control even in the presence of RNase H1/H2^2,6,28,63^. Presumably, RNase H enzymes can efficiently cleave RNA within relatively regular, accessible RNA-DNA duplexes but cannot effectively act on chromatin-embedded, protein-stabilized, or structurally complex R-loops before they are remodeled by helicases and other factors. Furthermore, our data indicate that persistent R-loops at DSBs in HELZ-deficient cells hinder DNA2 recruitment and DNA end resection (Fig. 6), thereby compromising HR, particularly at actively transcribed genomic regions (Fig. 4). Consequently, HELZ depletion or helicase inactivation leads to increased R-loop burden, impaired DNA repair responses, and reduced cell survival following DNA damage, highlighting HELZ as a critical R-loop resolvase that licenses efficient transcription-coupled HR and safeguards genome stability.

Interestingly, several helicases have been implicated in R-loop regulation at DSBs, but available data suggest that their functions are not simply redundant and operate at distinct steps of HR. Senataxin (SETX) promotes RAD51 loading without accelerating repair kinetics, instead preventing inappropriate rejoining and suppressing chromosomal translocations^63^, indicating a primary role in repair fidelity rather than resection efficiency. DDX5, recruited by BRCA2, has been reported to unwind RNA-DNA hybrids predominantly after resection to facilitate RPA/RAD51 loading; we note, however, that BRCA2-DDX5 may also contribute to earlier steps, as reduced RPA foci are observed in cells expressing BRCA2 mutants defective in DDX5 binding^28^. DHX9 functions even upstream by recruiting BRCA1 and CtIP for initiation of resection^64^, thereby licensing HR entry. In contrast, our data position HELZ at a stage where transcription-coupled R-loops physically block access of long-range resection machinery: HELZ loss leaves MRN and CtIP recruitment intact but markedly reduces DNA2 loading and DNA end resection, demonstrating that HELZ is required not for short-range resection factor recruitment, but for clearing RNA-DNA hybrids that impede resection progression. Together, these distinctions support a multi-layered model in which SETX preserves fidelity, DHX9 enables BRCA1-CtIP initiation, DDX5 acts after resection, and HELZ uniquely removes transcription-associated R-loops, permitting the initial incision reaction, DNA2-dependent resection extension, or both, while RNase H1/H2 provides essential but context-limited degradation of accessible hybrids.

### Role for BRCA2-DSS1 in RNA-DNA hybrid resolution and its link to cancers in estrogen-rich tissues

Our findings reveal a previously unrecognized function for the BRCA2-DSS1 complex in promoting R-loop resolution at DSBs through its interaction with HELZ to facilitate DNA end resection, extending its well-established roles in RAD51 loading onto ssDNA during HR, ssDNA-gap suppression, and RFP^15,18–22,26^. BRCA2-DSS1 directly engages HELZ, stimulates its RNA helicase activity, and promotes its recruitment to R-loops, likely through BRCA2^CTRB^-HELZ complex formation together with DSS1-mediated stabilization, nuclear localization, and ssDNA-binding modulation of BRCA2^15,26^. Previous studies reported that BRCA2 also interacts with DDX5 and RNase H2 through its N-terminus and BRC repeats, respectively, and cooperates with these factors to resolve RNA-DNA hybrids on resected ssDNA, thereby facilitating RPA or RAD51 loading without affecting the extent of DNA end resection^28,29^. In contrast, our data support a model in which the BRCA2-DSS1-HELZ axis promotes efficient DNA end resection and thereby supports downstream HR. Together, these findings provide mechanistic insights into how BRCA2-DSS1 maintains R-loop homeostasis at mechanistically and temporally distinct steps^22,26–29^ and broaden the functional scope of BRCA2-DSS1 beyond its canonical role in RAD51 loading, although further work will be required to fully substantiate this model.

Of note, the mechanism by which BRCA2 mutations promote cancer in estrogen receptor-positive (ER⁺) tissues^65–67^, such as breast and ovary, has remained a long-standing question in the BRCA research field. Our study proposes that BRCA2-HELZ-mediated R-loop resolution can play a key role in suppressing breast and ovarian cancer. Estrogen signaling drives elevated transcription in hormone-sensitive tissues, increasing the propensity for R-loop formation^53^. In this setting, the BRCA2-HELZ complex likely performs an essential function in resolving transcription-associated R-loops, thereby preserving genome stability and preventing tumorigenesis. Notably, HELZ knockdown in ER⁺ cells significantly reduces cell survival following treatment with the PARP inhibitor olaparib, highlighting its critical role in HR repair under genotoxic stress. This specific reliance on HELZ for genome maintenance in estrogen-rich tissues may explain the tissue specificity of BRCA2-associated cancers and underscores the importance of R-loop regulation in this oncogenic context.

### HELZ as a biomarker and therapeutic target in R-loop-enriched cancers

Considering that HELZ knockout mice are viable, fertile, and phenotypically normal, with no apparent skeletal defects (MGI:1925705), our findings suggest that HELZ is specifically required for the survival of certain cancer cells by resolving R-loops. This context-specific essentiality positions HELZ as a promising therapeutic target in cancers characterized by elevated R-loop levels, such as ER⁺ breast cancers and Ewing sarcoma. Moreover, the functional interaction between HELZ and BRCA2 defines an R-loop resolution pathway that may be leveraged for precision therapies targeting R-loop-associated vulnerabilities, especially in BRCA2-mutant cancers. Future studies should systematically evaluate HELZ expression and R-loop levels across cancer types and investigate the utility of HELZ as a therapeutic target, particularly in combination with DNA-damaging agents.

## Methods

### Plasmid construction

Human *HELZ* was amplified via PCR using pT7-EGFP-C1-HsHelz_AG (gift from Elisa Izaurralde, Addgene plasmid#148799) as the template and cloned into pFastbac vector (i.e., pFastbac-Twin-StrepTagII-HELZ) to express the full-length HELZ with Twin-StrepTagII in insect cells. QuikChange site-directed mutagenesis was used to create pFastbac-Twin-Strep-Tag-HELZ^K674N^ and pT7-EGFP-C1-HELZ^K674N^. pDest8-MBP-His-BRCA2-Flag, pDest8-His-miBRCA2 (containing BRC repeat 4 (BRC4; residues 1496-1596), the DNA-binding domain (DBD; residues 2477-3194), and the C-terminal RAD51-binding domain (CTRB; residues 3195-3418)-Flag, pDest8-His-DBDCT-Flag, pDest8-His-DBD-Flag and pDest20-DSS1 were used for the expression of BRCA2-DSS1, miBRCA2-DSS1, DBDCT-DSS1, DBD-DSS1 in insect cells as described^23^. pFastbac-Flag-BRCA1 and pFastBac-Twin-StrepTagII-BARD1(gift from Andrew Deans, Addgene plasmid #137166)^68^ were used for BRCA1 and BARD1 expression in insect cells as described^69,70^. pET32a-Flag-CTRB was used for expressing the CTRB domain of BRCA2 expression in bacterial cells as described^71^. pcDNA5/FRT-HA-Twin-Strep-Tag-GFP-HELZ and pcDNA5/FRT-HA-Twin-Strep-Tag-HELZ were used for HELZ expression in mammalian cells. QuikChange site-directed mutagenesis was used to create siRNA/shRNA-resistant wild-type or the mutant K674N form of HELZ in the above expression vectors. The plasmids phCMV1-2xMBP-BRCA2, pDest 3xFlag-pcDNA5-FRT/T0-GFP-DSS1^26^, pDest 3xFlag-pcDNA5-FRT/T0-BioID-DSS1, and pFRT-DestFlag-HA-RNase H1 (WT or D210N)-V5 were constructed and used for BRCA2, DSS1 and RNase H1(WT or D210N) expression in mammalian cells.

### Protein purification

#### Purification of HELZ from insect cells

The bacmid production in *E. coli* strain DH10Bac, baculovirus generation and amplification in SF9 cells, and protein expression in Hi5 cells were performed as described^72^. All purification steps were carried out at 0 °C to 4 °C. To prepare the extract, the frozen cell pellet (5 g, from a 300 ml culture) was thawed and suspended in 50 ml of cell breakage buffer A (50 mM Tris-HCl, pH 8.0, 500 mM KCl, 1 mM EDTA, 0.01% Igepal-CA630, 1 mM 2-mercaptoethanol (β-ME), 10% Glycerol, 5 mM MgCl_2_, 2 mM ATP, and the following protease inhibitors: aprotinin, chymostatin, leupeptin, and pepstatin A at 3 mg/ml each, 1 mM PMSF, 0.9 mg/ml benzamidine hydrochloride). Cell lysis was performed using 10% strength sonication (Microtip, Branson Digital Sonifier) for 3 min (2 s on, 5 s off). The lysate was cleared by centrifugation at 40,000 × *g* for 30 min, filtered through a 0.45 μm PES filter (Thermofisher, FB12566509), and loaded onto a 1 ml StrepTrap XT Chromatography Column (Cytiva, 09920071) at 0.5 ml/min. After washing the column with 50 ml of buffer B (50 mM Tris-HCl, pH 8.0, 300 mM KCl, 0.5 mM EDTA, 0.01% Igepal-CA630, 1 mM β-ME, 10% Glycerol, 5 mM MgCl_2_, 2 mM ATP, and the following protease inhibitors: aprotinin, chymostatin, leupeptin, and pepstatin A at 0.03 mg/ml each, 1 mM PMSF, 0.9 mg/ml benzamidine hydrochloride), the bound proteins were eluted with 10 ml of buffer B containing 50 mM biotin at 0.5 ml/min. The eluates were mixed with 50 ml of buffer C (25 mM Tris-HCl, pH 8.0, 10% Glycerol, 0.5 mM EDTA, 0.01% Igepal-CA630, 1 mM DTT) before being further fractionated on a 1 ml HiTrap Heparin HP column (GE Healthcare) using a 10 ml gradient of 50-500 mM KCl in buffer D (20 mM Tris-HCl, pH 8.0, 10% Glycerol, and 1 mM DTT). The peak fractions were pooled and further fractionated on a 1 ml HiTrap Q HP column (GE Healthcare) using a 10 ml gradient of 50–500 mM KCl in buffer D. The peak fractions were concentrated to under 1 ml in buffer E (20 mM Tris-HCl, pH 7.5, 150 mM KCl, 10% Glycerol, and 1 mM DTT), divided into 10 μl portions, frozen in liquid nitrogen, and stored at −80 °C. The mutant forms of HELZ were expressed and purified using the same procedures.

#### Purification of BRCA2-DSS1 and its variants

Full-length BRCA2-DSS1, miBRCA2-DSS1, DBDCT-DSS1, DBD-DSS1 and GST-DSS1 were expressed in insect cells and purified using our previously described procedures^23,26,71^. Briefly, recombinant bacmids encoding full-length BRCA2, miBRCA2, DBDCT, DBD, and DSS1 were generated in DH10Bac cells, amplified in Sf9 cells, and used to infect Hi5 cells. Infected cells were harvested, flash-frozen, and stored at −80 °C. BRCA2, BRCA2-DSS1, miBRCA2-DSS1, DBDCT-DSS1, and DBD-DSS1 were purified by anti-Flag affinity chromatography, followed by glutathione affinity chromatography and Q Sepharose ion-exchange chromatography. For miBRCA2-DSS1, DBDCT-DSS1, and DBD-DSS1, an additional size-exclusion chromatography step was performed using a Superdex 200 Increase 10/300 GL column. Peak fractions were pooled, aliquoted into 10 μl portions, snap-frozen in liquid nitrogen, and stored at −80 °C.

GST-DSS1 and CTRB-Flag were expressed in bacterial cells and purified using our previously described procedures^23,71^. Briefly, GST-DSS1 was purified by glutathione affinity and Q Sepharose chromatography. CTRB-Flag was purified using Ni-NTA affinity chromatography, anti-Flag resin, and Mono Q ion-exchange chromatography. Peak fractions were pooled, concentrated, flash-frozen, and stored at −80 °C.

### Other recombinant proteins

BRCA1-BARD1 was expressed in insect cells and purified using our previously described procedures^69,70,72^. Briefly, BRCA1-BARD1 was purified by StrepTrap XT affinity chromatography followed by HiTrap SP cation-exchange chromatography. Peak fractions were pooled, aliquoted, snap-frozen in liquid nitrogen, and stored at −80 °C. BLM was expressed in protease-deficient Saccharomyces cerevisiae JEL-1 and purified as described previously^73^ using Ni-NTA affinity, Source Q, hydroxyapatite, and Mono S chromatography. Peak fractions were pooled, concentrated, flash-frozen, and stored at −80 °C.

### Mass photometry

All mass photometry experiments were performed with a Refeyn TwoMP instrument in 1× Buffer F (25 mM Na HEPES pH 7.5, 1 mM DTT, and 100 mM KCl). Samples were flash diluted to 20 nM protein in 1× phosphate-buffered saline (PBS). Data were acquired for 1 min, and figures are representative images of at least 3 independent measurements. Ratiometric counts were converted to molecular weight in kDa using a standard curve generated with ovalbumin (43 kDa), conalbumin (75 kDa), aldolase (158 kDa), and thyroglobulin (669 kDa). The data were analyzed with Refeyn’s DiscoverMP software.

### RNA and DNA substrates, EMSA binding assay and HELZ unwinding assay

#### RNA and DNA substrates

All DNA and RNA oligonucleotides used in the in vitro analysis were commercially synthesized and purchased from IDT and Sigma. R-loops, D-loops, DNA bubbles, RNA-DNA flaps, RNA/DNA hybrids with 5′ RNA overhang, RNA/DNA hybrids with 3′ RNA overhang, RNA/DNA hybrids with no RNA overhang, pure RNA/DNA hybrids, dsRNA and dsDNA were assembled from oligonucleotides 1/2/3, 1/2/4, 1/2, 5/6/7, 8/9, 8/10, 8/12, 3/11, 3/13 and 4/11, respectively, as described previously^72,74^. The single-stranded RNA substrate was a 5′ FAM-labeled oligonucleotide 3, 14, 15 and 16. All oligo sequences used for substrates are listed in Supplementary Table 1.

#### EMSA binding assay

5 nM of R-loop, D-loop, DNA bubble, RNA/DNA hybrid (5′ RNA overhang, 3′ RNA overhang or no RNA overhang), dsDNA, ssDNA, ssRNA or dsRNA substrate was mixed with HELZ protein at the concentrations indicated, in a buffer composed of 10 mM Tris-HCl (pH 7.5), 60 mM KCl, 100 ng/μl BSA, 2 U/μl RNaseIN (Promega), and 1 mM DTT, with a final volume of 12.5 μl. This mixture was incubated for 30 min on ice, followed by the addition of 2.5 μl of loading buffer composed of 50% Glycerol, 120 mM Tris-HCl (pH 7.4), 0.5 mM EDTA, and 0.05% Orange G. Electrophoretic separation of the protein-bound substrates was carried out by running the mix on 5% polyacrylamide gels with TAE (40 mM Tris, 20 mM acetic acid, 1 mM EDTA) buffer at 70 V for 60 min at 4 °C. The gels were directly imaged using the Cy5, DL800, or Alexa488 channel of the ChemiDoc MP Imaging System. The data was analyzed with Image Lab software.

#### RNA-DNA flap/hybrid/R-loop unwinding assay

5 nM of RNA-DNA flap/hybrid/R-loop was mixed with the HELZ protein at the concentrations indicated, in a buffer composed of 10 mM Tris-HCl (pH 7.5), 60 mM KCl, 100 ng/μl BSA, 1 mM MgCl_2_, 2 mM ATP, and 1 mM DTT, with a final volume of 12.5 μl. The mixture was incubated at 37 °C for 15 min. The reaction was terminated by adding 2.5 μl of loading buffer composed of 50% Glycerol, 20 mM Tris-HCl (pH 7.4), 0.5 mM EDTA, 0.05% Orange G, 0.5 μl 10% SDS, 1 μl 1 mg/ml proteinase K, followed by a 15-min incubation at 37 °C.

For unwinding assays with R-loops, D-loops, or RNA/DNA hybrids (5′ RNA overhang, 3′ RNA overhang or no RNA overhang) substrates with 5′ or 3′ overhangs, a 100-molar excess of competitor oligonucleotide (Oligo 17 for the reactions of R-loop and D-loop, Oligo 18 for the reactions of RNA/DNA hybrids with 5′ or 3′ overhangs) was added after the HELZ mediated unwinding reactions to trap the unwound oligonucleotides and prevent reannealing. Electrophoretic separation of the unwound substrates was carried out by running the mix on 5% polyacrylamide gels with TAE buffer at 70 V for 60 min at 4 °C. The gels were directly imaged using the Cy5, FAM, or DL800 channel of the ChemiDoc MP Imaging System. The data were analyzed with Image Lab software.

### R-loop and control dsDNA substrates, AFM sample preparation and image analysis

#### R-loop and control dsDNA substrates

Linear DNA containing position-specific R-loops (R-loop DNA) was generated through in vitro transcription of pFC53-*Airn* plasmid (3991 bp) as previously described^36,37^. pFC53-*Airn* contains the mouse *Airn* sequences downstream of a T3 promoter, which promotes R-loop formation when transcribed. Transcription reactions (50 μl total, 3 μg pFC53 DNA and 4.5 μl T3 RNA polymerase at 18.4 U/μl) were carried out on circular pFC53-*Airn* at 37 °C for 30 min in 1× Transcription Optimized Buffer (40 mM Tris-HCl, pH 7.9, 6 mM MgCl_2_, 2 mM spermidine and 10 mM NaCl) supplemented with DTT (20 mM), Tween-20 (0.05%), and rNTPs (50 μM). The reactions were heated at 65 °C for 10 min to terminate transcription. Unpaired RNA was degraded by incubating with RNase A (5 μl of 0.1 mg/mL, Thermofisher) at 37 °C for 30 min. Samples were incubated with Proteinase K (2 μl of 18.5 mg/mL, Roche) for 30 min at 37 °C to remove T3 RNA polymerase and RNase A. Gel electrophoresis was used to confirm R-loop formation. The circular R-loop DNA was then linearized with ApaLI. To generate a negative control DNA without R-loops (control dsDNA), the pFC53-*Airn* plasmid was linearized with ApaLI directly. Linearization generated a short and long linear DNA fragment of distinct lengths (1246 bp and 2745 bp, respectively) that can be differentiated in AFM images^36^. The DNA substrates were further purified using the Zymo DNA Clean and Concentrator kit before AFM imaging. In the R-loop DNA sample, the longer DNA fragment contains the R-loop located at 38-42% of the total DNA length from the nearest DNA end. Thus, all R-loop and protein-binding positions were measured on the longer DNA fragments. The presence of R-loop was validated by the AFM imaging results showing that S9.6 antibody specifically localized at R-loops^36^ and by measuring its height (1.15 ± 0.16 nm), which is greater than dsDNA (0.42 ± 0.05 nm).

#### AFM sample preparation and image analysis

Purified linear R-loop DNA or control dsDNA (5 nM) was incubated in the presence of HELZ (20 nM) in HELZ Reaction Buffer (40 mM Tris-HCl, pH 7.6, 50 mM NaCl, and 2 mM MgCl_2_) at room temperature for 20 min. The reactions were diluted eightfold in the AFM imaging buffer (20 mM HEPES, pH 7.0, 100 mM NaCl, and 10 mM Mg(C_2_H_3_O_2_)_2_) and immediately deposited onto a freshly cleaved mica surface (SPI Supply). The samples were rinsed with deionized water and dried under a stream of nitrogen gas. The samples were then imaged using AFM imaging in air with the AC mode on an MFP-3D-Bio AFM (Asylum Research, Oxford Instruments) using Pointprobe PPP-FMR probes (Nanosensors, spring constants at ~2.8 N/m). All images were collected at scan sizes of 3 × 3 μm^2^, a scan rate of 1 Hz, and a resolution of 512 × 512 pixels. The Asylum Research AFM software was used to measure protein binding position and height with the “Section” function of the “Analyze Panel” and protein volume with the “Particle Analysis” module. A minimum of 2–3 independent repeats were collected for each sample condition.

#### Affinity pull-down

0.5 μM Flag-miBRCA2/GST-DSS1, Flag-DBDCT/GST-DSS1, Flag-DBD/GST-DSS1, GST-DSS1, CTRB-Flag, and Flag-BRCA1-BARD1 was incubated with 0.2 μM HELZ at 4 °C for 30 min in 30 μl buffer G (25 mM Tris-HCl, pH 7.5, 10% Glycerol, 0.5 mM EDTA, 0.05% Igepal CA630, 1 mM 2-mercaptoethanol, and 100 mM KCl). The reaction mixture was then mixed with 10 μl Glutathione Sepharose 4B (Cytiva) or anti-Flag M2 affinity resin (Sigma) at 4 °C for 30 min to capture protein complexes via the GST-tag on DSS1 or Flag-tag on BRCA2. After washing the resin three times with 200 μl buffer G, bound proteins were eluted with 20 μl 2% SDS at 37 °C for 5 min. The supernatant (S), last wash (W), and SDS eluate (E), 8 μl each, were analyzed by SDS-PAGE and Coomassie blue staining. For the full-length BRCA2 and HELZ affinity assay, an anti-BRCA2 antibody and protein G beads were used for capturing the protein complex.

### Mammalian cell culture and transfection

HEK293T (ATCC), HeLa (ATCC), U2OS (DR-GFP, gift from Dr. Jeremy Stark) were grown in Dulbecco’s modified Eagle's medium (DMEM) supplemented with 10% fetal bovine serum (Sigma), 100 μg/ml streptomycin, and 100 μ/ml penicillin (Sigma). DLD1 and DLD1-BRCA2 knockout cells were gifts from Dr. Ryan Jensen. The cells were tested for mycoplasma contamination by Bionique Testing Labs (http://www.bionique.com/). Control siRNA (CUUACGCUGAGUACUUCGAdTdT), HELZ siRNA (#1: CAGCACACCUUGUUAAAUC dTdT; #2: GAUAUCACGUGGAAGACUUdTdT), BRCA1 siRNA (AAGCUCCUCUCACUCUUCAGU), BRCA2 siRNA (UUGGAGGAAUAUCGUAGGUAA) oligonucleotides were purchased from Sigma. Dharmacon™ TRIPZ™ lentiviral shRNAs against BRCA1 (V2THS) and HELZ (V2THS_95825 and V3THS_362670) were purchased from Horizon-PerkinElmer. Transfection of siRNA, mammalian expression vectors, shRNA and pCMV-I-SceI-3×NLS was carried out using Lipofectamine 2000 (Invitrogen) according to the manufacturer’s instructions. To create a FLP-in version of HeLa, we stably integrated a flippase recognition target (FRT) sequence into the cells by using the pFRT/lacZeo plasmid (Thermo Fisher Scientific). We tested Zeocin-resistant clones that had a single integration site detected by Southern blot for high-activity integration sites by using the mammalian b-galactosidase activity assay (Gal-Screen, Thermo Fisher Scientific). Clonal expansion of the selected colony established the HeLa-FRT cell line. To generate stable HeLa-FRT shHELZ cells, two TRIPZ™ lentiviral shRNAs against HELZ were transfected with their respective plasmids, and individual clones were selected with 2 μg/ml puromycin.

### Proximity-dependent biotinylation (PDB) assays and mass spectrum analysis

Overview of project: Immunoprecipitation (IP) experiments were performed across three independent projects, yielding a total of seven IP samples for this analysis. In the first project, four IP samples were generated, consisting of two experimental groups: a negative control (*n* = 1) and a positive experimental group (*n* = 3). The remaining three IP samples were collected from two additional IP projects, each contributing negative-control samples (*n* = 1 and *n* = 2, respectively). For the final integrated analysis, all seven IP samples were evaluated together, representing two combined experimental groups: negative controls (*n* = 4 biological replicates) and positive samples (*n* = 3 biological replicates).

Plasmids pDest 3xFlag-pcDNA5-FRT/TO-BioID-DSS1 or pDest 3xFlag-pcDNA5-FRT/TO-BioID were transfected into HeLa-FRT-shDSS1 cells. Stable cell lines (HeLa-shDSS1-BioID-DSS1 and HeLa-shDSS1-BioID) were then generated using the Flp-In method as previously described^26^. HeLa-shDSS1-BioID-DSS1 cells were pretreated with doxycycline (2 μg/ml) for 48 h to induce depletion of endogenous DSS1 (positive experimental group), whereas HeLa-shDSS1-BioID cells were mock-treated with DMSO (negative control). Cells were subsequently incubated with olaparib (10 μM) and biotin (100 μM) for 24 h prior to harvesting.

#### Cell lysis and protein extraction

Pelleted nuclei were isolated and lysed in 800 μl NETN420-SDS lysis buffer (20 mM Tris-HCl, pH 7.5, 420 mM NaCl, 1 mM EDTA, 1% NP-40, 0.1% SDS, 0.5% sodium deoxycholate, 6 mM MgCl₂, and 1 mM DTT) supplemented with protease inhibitors. The lysate was incubated on ice for 10–15 min, followed by two rounds of sonication (5 s each, 10% amplitude). To further digest chromatin, 25-50 U of NucA (in-house benzonase, a non-specific nuclease that degrades both DNA and RNA) was added, and the mixture was rotated at 4 °C for 30 min. The lysate was centrifuged at maximum speed for 20 min, and the supernatant was collected, reserving 50 μl for Western blot analysis.

#### Affinity purification

Dynabeads MyOne Streptavidin T1 beads (Invitrogen) were washed with NETN420-SDS lysis buffer before being evenly aliquoted (30–50 μl per sample). The beads were incubated with 750 μl of the cleared supernatant on a rotator at 4 °C overnight. The next day, the beads were spun down, and the supernatant was removed.

#### Bead washing steps

1. Two washes with NETN300-SDS lysis buffer (20 mM Tris, pH 7.4, 0.2% SDS, 0.1% Triton X-100, 1% NP-40, 300 mM NaCl, 1 mM EDTA, 0.5% sodium deoxycholate, 1 mM DTT) with 10-min rotations.
2. One wash with 1 M KCl for 10 min on a rotator.
3. One wash with urea wash buffer (2 M urea, 10 mM Tris-HCl, pH 7.5) (brief mixing, no rotation).
4. Two to three washes with NETN420-SDS lysis buffer.
5. Three washes with PBS to remove residual SDS that may interfere with trypsin digestion.
6. Two washes with cold 100 mM ammonium bicarbonate (AMBIC) (freshly prepared and stored at 4 °C). The second AMBIC wash was performed in new tubes.

Beads were either processed immediately or snap-frozen and stored at −20 °C or −80 °C for up to 2 months before mass spectrometry analysis.

#### Mass spectrometry and data analysis

1. Sample preparation (digestion from the beads): FASP (filter-aided sample preparation) method was used to wash, reduce, alkylate and digest proteins still attached to the immunoprecipitation beads as described^75^. Briefly, 30 μl beads were reduced with 10 mM TCEP (Tris (2-carboxyethyl) phosphine hydrochloride) at a pH of 8.5 by incubating for 30 min at 37 °C. FASP filter (30 kDa MW cutoff, spin filter) was washed with 400 ul of HPLC-grade water by centrifuging at 14,000 × *g* for 10 min. Urea (8 M) was added to each sample at 11 times the sample volume and then passed through the washed FASP filters with centrifugation at 14,000 × *g* for 15 min until completed. Sample on filter was then alkylated with 100 μl of 30 mM IAA (Iodoacetamide) in the dark without mixing for 20 min. Filters with sample were washed with 100 μl of 8 M Urea (3 times with centrifugation at 14,000 × *g* for 15 min) and then 300 μl of 100 mM TEAB (Triethylammonium bicarbonate) 3 times with similar centrifugation cycles. Filters were transferred to new collection tubes. Proteins were digested on a filter by adding 200 μl 100 mM TEAB to 1 μg of sequencing grade modified porcine trypsin (Promega), vortexed briefly at low speed and incubated in a wet chamber at 37 °C for 18 h. On the second day the filters were centrifuged at 14,000 × *g* for 10 min. To ensure peptide removal from the filter 80 μl of 100 mM TEAB was added and centrifuged in a similar manner. The pH was lowered to stop any additional enzymatic activity by adding 20 μl of 5% Formic Acid/1% Trifluoroacetic Acid solution. Samples were desalted using SepPak plates from Waters using the recommended instructions from the manufacturer.
2. Separation and data collection: Tryptic peptides were then separated by reverse phase XSelect CSH C18 2.5 μm resin (Waters) on an in-line 150 ×0.075 mm column using an UltiMate 3000 RSLCnano system (Thermo). Peptides were eluted using a 60 min gradient from 98:2 to 65:35 buffer A:B ratio with a constant flow rate of 300 nl/min (Buffer A = 0.1% formic acid, 0.5% acetonitrile; Buffer B = 0.1% formic acid, 99.9% acetonitrile). Eluted peptides were ionized by electrospray (2.4 kV) followed by mass spectrometric analysis on an Orbitrap Eclipse Tribrid mass spectrometer (Thermo). Data-dependent acquisition was used to acquire data. MS data were acquired using the FTMS analyzer in profile mode at a resolution of 120,000 over a range of 375 to 1200 *m*/*z*. Following HCD activation, MS/MS data were acquired using the ion trap analyzer in centroid mode and normal mass range with a normalized collision energy of 30%.
3. Database search: Proteins were identified by database search using MaxQuant (Max Planck Institute) with a parent ion tolerance of 3 ppm and a fragment ion tolerance of 0.5 Da against a UniprotKB *Homo sapiens* proteome (Proteome ID: UP000005640, Taxon ID: 9606 accessed on 01/2022). Fixed modification was set to Carbamidomethyl (C), variable modifications set to Oxidation (M), Acetyl (Protein N-terminal). The number of missed cleavages was set to a maximum of 2 and only peptides with a minimum length of 8 aa and a maximum length of 25 aa were considered. Scaffold Q+S (Proteome Software) was used to verify MS/MS-based peptide and protein identifications. Protein identifications were accepted if they could be established with less than 1.0% false discovery and contained at least 2 identified peptides. Protein probabilities were assigned by the Protein Prophet algorithm^76^.
4. Data analysis: Raw data is available via ProteomeXchange with identifier PXD066766. Data processing and statistical analysis were performed using R (version 4.4.0). Significant proteins were identified using a cutoff of ^*^p < 0.05 (two-sided Student’s *t*-test) and fold-change >2. Results were visualized using volcano plots to highlight differentially enriched proteins.

### Immunoprecipitation analysis

Twin-Strep-Tag-GFP-HELZ, MBP-BRCA2, Flag-GFP-DSS1 plasmids were transfected into HEK293T cell for 48 h in the 10 cm dishes, following two washes with PBS (phosphate-buffered saline), cells were scraped off and transferred to Eppendorf tubes. Whole cell lysate was prepared by adding 1 ml of lysis buffer T300 (300 mM NaCl, 50 mM Tris-HCl, pH 7.4, 1.0% Triton X-100, 5 mM EDTA) with protease inhibitors (Roche Complete Protease Inhibitor Tablet) and Simple Stop™ 2 Phosphatase Inhibitor Cocktail GB-451-1 (100X) to cell pellets.

Following 2X 5 s sonication, the cell extract was cleared by centrifugation at 13,000 × *g* for 20 min at 4 °C. The supernatant fraction was 1:1 diluted with T0 buffer (50 mM Tris-HCl, pH 7.4, 1.0% Triton X-100, 5 mM EDTA) with protease inhibitors and Simple Stop™ 2 Phosphatase Inhibitor Cocktail to the final T150. The T150 supernatant fraction (2 mg protein in total) was incubated with DNase I (20U) for 15 min at 4 °C. Then, 30 µl of Strep-Tactin XT Sepharose (Cytiva) or anti-Flag M2 affinity resin (Sigma) was added, followed by incubation at 4 °C overnight. After washing the resin 4 times with T150 buffer (150 mM NaCl, 50 mM Tris-HCl, pH 7.4, 1.0% Triton X-100, 5 mM EDTA), bound proteins were eluted with 30 µl SDS gel loading buffer (50 mM Tris-HCl pH 6.8, 2% SDS, 0.1% Bromophenol blue, 10% Glycerol, 10% 2-Mercaptoethanol) and the eluates were subject to Western blot analysis with anti-HA, anti-BRCA2 and anti-PALB2 antibodies. In experiments using stable HeLa-Twin-Strep-Tag-GFP-HELZ cells, cells were pretreated with CPT (5 µM) for 4 h prior to further processing. For immunoprecipitation with BRCA1 or BRCA2 antibodies, 2 µg of antibody was added to the supernatant and incubated overnight at 4 °C with rotation. Protein G beads were then added and incubated for an additional 2–4 h at 4 °C with rotation to capture the complexes.

### Immunoblot analysis

Protein was extracted from cells harvested 2 days after transfection with the indicated siRNAs or 3 days after 1 mg/ml doxycycline treatment using NETN buffer (20 mM Tris-HCl, pH 8, 420 mM NaCl, 1 mM EDTA, 0.5% Igepal CA630, 1 mM DTT, and Roche Protease Inhibitor Cocktail). Blots (20-50 mg of total protein) were probed with the following antibodies: HELZ (home-made, a gift from David Yu’s lab), HA (3724S, Cell Signaling; 1:1000), BRCA1 (SC6954, Santa Cruz; 1:500), BRCA2 (EMD Millipore, OP95-100UG; 1:1000), PALB2(home-made, a gift from Bing Xia’s lab), DSS1 (SC28848, Santa Cruz; 1:500), Tubulin (2128S, Cell Signaling; 1:2000), GAPDH (2118S, Cell Signaling; 1:15,000), Estrogen Receptor α (SC8002, Santa Cruz: 1:500), anti-Streptavidin-HRP (NC9705430, Jackson Immuno Research Labs; 1:3000), Flag M2-HRP (Sigma, A8592; 1:3000), p-ATM (S1981) (13050, Cell Signaling; 1:1000), p-Chk2 (T68) (2197, Cell Signaling; 1:1000), p-ATR (T1989) (30632, Cell Signaling; 1:1000), p-Chk1 (S345) (2348, Cell Signaling; 1:1000), and γH2AX (S139) (9718, Cell Signaling; 1:1000), according to the instructions provided by the manufacturers. If needed, the blots were incubated with HRP-conjugated secondary antibodies (Pierce 31450 for rabbit anti-mouse IgG-HRP; Sigma A6154 for goat anti-rabbit IgG-HRP) before visualization of protein signals using the ECL max kit (Biorad).

### DNA repair HR reporter assays

The DR-U2OS cell line containing a single integrated copy of the DR-GFP reporter was used^44^^,^^45^. Exponentially growing cells were seeded in 6-well plates at 2 × 10^5^ cells per well and subjected to reverse transfection with 2 μl siRNA (20 μM) and 3 μl Lipofectamine™ RNAiMAX. 24 h post-siRNA transfection, cells were transfected with 2 μg of Twin-Strep-Tag-HELZ or mutant constructs. 48 h after HELZ knockdown, cells were further transfected with 1.5 μg of the I-*Sce*I expression vector (pCBASce) and 3 μl of Lipofectamine™ 2000. HR proficiency was determined by counting the fraction of GFP-positive cells using a BD FACS Calibur S at 48 h or 72 h following I-*Sce*I transfection. Data represent results from 3 to 5 transfections across at least 3 independent experiments.

### Tet-DR-GFP reporter assay

The tetR-DR-GFP assay was conducted as described^47^. In brief, U2OS-Tet-DR-GFP reporter cells were seeded in 6-cm dish 24 h prior to siRNA transfection. Cells were pre-treated with either siHELZ or siControl (siCtrl) for about 16 h before plasmid transfection. Subsequently, 5 µg of the I-*Sce*I-T2A-mCherry plasmid was transfected using Lipofectamine 2000, following the manufacturer’s guidelines, for 8 h of incubation. Then, cells were trypsinized and split into two 6-cm dishes: one was induced with 1 µg/ml of doxycycline, while the other received a vehicle control. After 60–72 h of incubation, cells were harvested. Half of the sample was fixed in 1% PFA used for flow cytometry analysis, while the remaining portion was processed for gDNA extraction using the Wizard® Genomic DNA Purification Kit (Promega). The genomic DNA concentration was measured using NanoDrop, then diluted for qPCR using PowerUp SYBR Green Master Mix (Invitrogen) and analyzed on the StepOnePlus™ Real-Time PCR System (Applied Biosystems). Primers (5′-GGGCGATGCCACCTACG-3′) and (5′-GGTGTTCTGCTGGTAGTGGTCG-3′) were used to amplify repaired sceGFP, while primers (5′-CAGCAAGTGGGAAGGTGTAATCC-3′) and (5′-CCCATTCTATCATCAACGGGTACAA-3′) targeted a reference genomic locus. Each reaction was run in triplicate, and the experiment was performed three times with independent biological replicates.

### Immunofluorescence microscopy and image analysis

Cells were subjected to the treatment of 4 or 6 Gy gamma-irradiation (IR) followed by 3 washes with PBS, before being pre-extracted and fixed at different time points post-treatment. Pre-extraction was performed on ice for 10 min with cold cytoskeleton buffer (10 mM PIPES pH 6.8, 100 mM NaCl, 300 mM sucrose, 3 mM MgCl_2_, 1 mM EGTA, 0.5% Triton X-100) followed by 10 min with cytoskeleton stripping buffer (10 mM Tris-HCl pH 7.4, 10 mM NaCl, 3 mm MgCl_2_, 1% Tween 40 (v/v), 0.5% sodium deoxycholate). Next, cells were fixed with 4% paraformaldehyde on ice for 20 min, washed with PBS, and permeabilized for 10 min with 0.5% Triton X-100 in PBS. After being blocked in blocking buffer (0.2% Triton-X, 5% Goat serum, 2% BSA in PBS) on ice for 20 min, cells were incubated with primary antibodies in blocking buffer overnight at 4 °C in a humid chamber. The following primary antibodies were used RAD51(8875S, Cell Signaling; 1:500), RPA (MABE285, Millipore, 1:500), BrdU (347580, BD Biosciences; 1:20), BRCA1 (SC6954, Santa Cruz; 1:500), MRE11(NB100-473, Novus; 1:500) and CtIP (PA5-84133, ThermoScientific; 61141, Active Motif; 1:500). Then cells were washed in PBS 3 times, incubated with Alexa Fluor or FITC conjugated secondary antibodies (Invitrogen) for 1 h at room temperature, and stained with DAPI for another 15 min, before slides were mounted using antifade mounting media (9071, Cell signaling). Images were captured using a Nikon Swept Field fluorescence microscope with the 40x oil lens. The average number of RAD51 and RPA foci per nucleus and the percentage of cells that were positive for these foci positive were determined after scoring at least 100 nuclei. Images were generated by the confocal microscope (FV3000; Olympus) and analyzed foci formation using ImageJ (1.53a version; NIH) software.

### Clonogenic survival assay

HeLa cells (parental or stably expressing Twin-Strep-Tag-GFP-HELZ), along with DLD1, MCF7, or TC32 cell lines, were transfected with control or HELZ siRNA for 48 h. HeLa-shHELZ cells were treated with doxycycline for over 66 h to induce HELZ knockdown. Then, 200–800 cells/well were seeded into 12-well plates, treated with indicated amount of olaparib (Selleckchem), camptothecin (Sigma) or indicated IR doses in regular growth medium for 11–12 days. Cells were fixed with methanol and stained with 0.5% crystal violet in methanol before colonies were counted. Clonogenic survival was determined for a given concentration of cells that were plated by dividing the number of colonies on each treated plate by the number of colonies on the untreated plate, taking the plating efficiency of untreated cells into account.

### DART assay with immunofluorescence staining

U2OS-TRE cells were seeded in a 35 mm glass-bottom dish (MatTek, P35GC-1.5-14-C). For the DART assay, cells were exposed to a 15W Sylvania cool white fluorescent bulb for 20 min in a stage UVP (Upland, CA) for Killer-red (KR) activation. Cells were transfected with plasmids and siRNA 24 to 36 h before KR activation. Plasmids used in this study are GFP-empty vector and GFP-tagged HELZ. siRNAs used in this study are siControl (siCtrl) and siBRCA2, siBRCA1 and double knockdown. Transfection was done using Lipofectamine 2000 (Invitrogen) with a standard protocol. After KR activation, cells were recovered for 20 min at 37 °C before fixation. For fixation, cells were first rinsed with phosphate-buffered saline three times (PBS, BE17-516F) and fixed in 4% paraformaldehyde (PFA; Affymetrix, 19943 1 LT) for 15 min at room temperature. They were then washed with PBS on the rotating bed for 5 min and permeabilized using 0.1% Triton X-100 in PBS for 10 min followed by another 5 min PBS wash on the rotating bed. Image acquisition was performed using an Olympus FV1000 confocal microscopy system (Cat. F10PRDMYR-1, Olympus). The frequency of the foci-positive cells was counted in thirty cells. Three independent experiments were done. The mean intensity was calculated by dividing the measured intensity of the selected area that colocalized with KR foci by ImageJ 1.52i software over the same size arbitrarily selected three areas in the nucleus (*n* = 10).

### PLA (proximity ligation assay)

To analyze colocalization of GFP-BRCA2 and HA-HELZ in HeLa cells upon camptothecin (CPT) treatment, cells were transfected with GFP-BRCA1 and HA-HELZ expressing plasmids for 48 h and then proximity ligase assays (PLAs) were carried out using Duolink PLA kit (Sigma-Aldrich; DUO92101). To induce R-loop accumulation by inhibiting of topoisomerase 1 activity^77^, cells were treated with CPT (1 μM, 1 h). The cells were washed with PBS and fixed with 2% of formaldehyde/PBS for 15 min. The fixed cells were washed twice with PBS and pre-permeabilized with 0.5% Triton X-100 in PBS for 10 min at room temperature. The cells were blocked with Duolink Blocking buffer for 1 h at 37 °C with humidity. Next, the cells were incubated with anti-GFP (Santa Cruz; sc-9996) and anti-HA (Cell Signaling Tech.; 3724S) antibodies in Duolink antibody diluent at 4 °C overnight. In Situ PLA probes (anti-mouse plus and anti-rabbit minus) were diluted 1:5 in Duolink antibody diluent and incubated to detect anti-GFP (mouse) and anti-HA (rabbit) antibodies for 1 h at 37 °C. After being washed with 1× Wash buffer A solution three times for 5 min each, dishes were incubated at 37 °C for 30 min with 1× Duolink ligation buffer solution and then washed twice with 1× Wash buffer A solution for 5 min each. For amplification signals, 1× Amplification mix solution was prepared as per manufacturer’s instructions and added into dishes for incubation at 37 °C for 100 min in the dark. To detect GFP-BRCA2 and HA-HELZ signals, cells were incubated with anti-GFP (Santa Cruz; sc9996) and anti-HA (Cell Signaling Technology; 3724S) primary antibodies, followed by goat anti-mouse IgG Alexa Fluor 488 (Abcam; ab150077) and goat anti-rabbit IgG Alexa Fluor 647 (Abcam; ab150079) secondary antibodies. Lastly, dishes were washed with 1× Wash buffer B solution three times for 5 min each and 0.01× diluted Wash buffer B solution once for 5 min before being mounted with emulsion oil, including DAPI and applied to the confocal microscope (FV3000; Olympus). Foci formation was analyzed using ImageJ (1.53a version; NIH) software.

### Preparation of cytoplasmic and nuclear extracts

The REAP method for the preparation of cytoplasmic and nuclear extracts was followed^26,78^. Briefly, HeLa cells from 6-cm dishes were washed with ice-cold phosphate buffer saline (PBS) pH 7.4, collected by centrifugation, resuspended in 200 μl of ice-cold PBS with 0.1% NP40 and protease inhibitors, and triturated five times using a p200 micropipette. The lysed cell suspension was centrifuged for supernatant (this is the cytoplasmic fraction), and the pelleted nuclei were washed once with PBS and lysed by 200 μl NETN600 buffer with protease inhibitors to yield the nuclear extract fraction. The cytoplasmic and nuclear fractions (20 μg/each sample) were analyzed by immunoblotting for their content of HELZ, BARD1, GAPDH, Tubulin and Lamin B1.

### qPCR-based DNA end resection assay in DIvA cells

The DNA end resection was tested in DIvA cells^79^. Briefly, 5 × 10^5^ DIvA (AsiSI-ER-U2OS) cells (a kind gift from Dr. Gaelle Legube’s lab) were seeded in 10-cm dishes and transfected with siCtrl, siCtIP or siHELZ using RNAiMax. 48 hr post transfection, one set of transfected siRNAs was left untreated and other set site-specific DSBs were induced by treatment with 300 nM of 4- Hydroxytamoxifen (4OHT; Sigma) for 4 h. Cells are then washed with 1 ml of ice-cold PBS twice and then scrapped and pelleted by centrifugation (5 min, 700 × *g*). Genomic DNA (gDNA) was isolated using the DNeasy kit (Qiagen) with RNase A included during lysis. To remove RNA:DNA hybrids, 500 ng gDNA was treated with 5 U RNase H (New England BioLabs) for 15 min at 37 °C and heat-inactivated for 20 min at 65 °C. RNase H-treated gDNA (140–150 ng) was then digested overnight at 37 °C with a restriction enzyme cutting at a defined distance from the AsiSI break site (BanI, 16 U; or BsrG1, 20 U; New England BioLabs) in 1× NEB restriction enzyme buffer 4, or mock digested, followed by heat inactivation (20 min, 65 °C). Digested or mock-digested DNA was quantified by PowerTrack SYBR Green reagent and qPCR using primers flanking the restriction site located ~200 bp or 350 bp from the AsiSI site. For BanI, the primer pair was forward: ACCATGAACGTGTTCCGAAT and reverse: GAGCTCCGCAAAGTTTCAAG and for BsrG1 the primer pair was forward: GAATCGGATGTATGCGACTGATC and reverse: TTCCAAAGTTATTCCAACCCGAT qPCR was performed using PowerTrack™ SYBR Green Master Mix (20 μl reactions with 0.5 μM primers; 7500 Fast Real-Time PCR System, ThermoFisher), using standard SYBR cycling conditions according to the manufacturer’s instructions. The percentage of single-stranded DNA (ssDNA%) generated by resection at each site was calculated as: ssDNA% = 1 / (2^ (ΔCt − 1) + 0.5) × 100, where ΔCt = Ct (digested) – Ct (mock/non-digested) for the corresponding sample.

### Laser-induced micro-irradiation

HeLa (shCtrl, shHELZ or shBRCA1) cells were treated with 2 µg/mL doxycycline for 48 h. Cells were seeded into glass-bottom dishes (Ibidi, Sigma) and maintained in doxycycline overnight. Then, cells were washed with 1× PBS and cultured in media without antibiotics for transfection with the GFP-nuclease construct as indicated, and 6 h post transfection, the media was changed to contain penicillin/streptomycin, doxycycline, and 10 µM BrdU for pre-sensitization. Laser micro-irradiation was performed the following day using a Zeiss LSM 900 confocal microscope with live cell incubation. Damage was achieved using a fixed-wavelength (405 nm) laser at 100% power. Images were taken every 15 s for 15 min post damage. Quantification analyzes were performed using the Zeiss Zen 3.9 software. To quantify, the mean GFP intensity at the damage site was normalized to the mean nuclear GFP intensity of the same cell at each timepoint.

### RNA/DNA hybrid slot blot

To examine the R-loops levels in HeLa cells expressing wild-type or mutants of Twin-Strep-Tag-GFP-HELZ, MCF7 cells, or TC32 cells, where endogenous HELZ was depleted by siHELZ (20 nM, 48 h), RNA/DNA hybrid slot blot assays were carried out as reported before^80^. Cells were lyzed with DNAzol (Invitrogen) and purified genomic DNA (gDNA) was sonicated (50% of power; 10/20 sec interval of ON/OFF x3). The concentration of gDNA sheared was measured by Nanodrop. After being treated with RNase III (100 U; Invitrogen, AM2290) and RNase T1 (100 U; ThermoFisher Sci., EN0541) at 37 °C for 15 min to cleave dsRNA and ssRNA, respectively, the indicated concentration of gDNA was applied to slot blot assay using Minifold I dot blot apparatus (Schleicher & Schuell). To remove RNA/DNA hybrid from gDNA, RNase H (10 U; NEB, M0297S) was incubated with gDNA at 37 °C for 1 h. The membrane was blocked for 1 h at room temperature in 5% milk and incubated overnight with the S9.6 antibody at a 1:1000 concentration. The membranes were developed using ECL detection reagents, and the signals were quantified using ImageJ software. Double-stranded DNA (dsDNA) antibody was used as a loading control.

### RNA/DNA hybrid staining assays with GFP-dRNH1

3 × 10^5^ cells on coverslips were rinsed twice with 1 ml of cold 1× PBS, fixed with 4% paraformaldehyde for 10 min on ice, rinsed twice again with cold 1× PBS, permeabilized with 0.25% Triton X-100 in 1 × PBS, and then rinsed twice more with 1 ml of cold 1× PBS. For RNase T1 and RNase III treatment, cells were rinsed twice with 200 μl low-salt buffer (50 mM Tris-HCl, pH 7.5, 75 mM KCl, 3 mM MgCl₂, 0.1% BSA), followed by enzymatic digestion with RNase T1 (1,000 U, Thermo Scientific, EN0541) and ShortCut RNase III (5 U, NEB, M0245S) in low-salt buffer for 3 h at 37 °C. For treatment with all three enzymes, cells were first incubated overnight at 37 °C with RNase H (20 U, NEB, M0297S) in 200 μl 1× RNase H buffer, washed with low-salt buffer, and then treated with RNases T1 and III in low-salt buffer as described above. Following enzymatic digestion, cells were washed briefly twice with 1× PBS and blocked with staining buffer (3% BSA in PBS) for 30 min. Samples were then incubated with purified GFP-dRNH1 (RNase H1^D210N^)^43^ at a 1:2000 dilution (0.188 mg/ml) in staining buffer for 2 h at 37 °C. Following three 5-min washes with PBST (0.05% Tween 20 in 1× PBS), coverslips were mounted with ProLong Glass Antifade Mountant with DAPI (Thermo Scientific, P36966), stored at 4 °C in the dark, and imaged within 48 h.

### Statistical analysis

The statistical analysis was performed using Prism 10 (GraphPad Software, Inc., La Jolla, CA; http://www.graphpad.com/quickcalcs/ttest1.cfm) on the data from at least three independent experiments, as specified. Statistical significance was assessed by two-tailed unpaired Student’s *t*-test and one/two-way ANOVA. ^*^P ≤ 0.05, ^**^P ≤ 0.01, ^***^P ≤ 0.001, and ^****^P ≤ 0.0001 were considered significant.

### Reporting summary

Further information on research design is available in the Nature Portfolio Reporting Summary linked to this article.

## Supplementary information

Supplementary Information

Reporting Summary

Transparent Peer Review file

## Data Availability

The Mass spectrometry data generated in this study have been deposited in the ProteomeXchange Consortium via the PRIDE partner repository with the identifier PXD066766. Source data is available in the Figshare Repository (10.6084/m9.figshare.30826055). Materials used in this research are available upon request from the correspondence authors.
