# Supplementary material for: HELZ-BRCA2 complex resolves R-loops to drive transcription-coupled homologous recombination
Source: Nat Commun. 2026 Jul 23;17:6969. doi: 10.1038/s41467-026-75088-4 (PMC13396364; doi:10.1038/s41467-026-75088-4)
Supplement: Supplementary file 1 — Supplementary Information [file 41467_2026_75088_MOESM1_ESM.pdf]

HELZ-BRCA2 Complex Resolves R-loops to Drive Transcription-coupled Homologous Recombination

Wenjing Li[1,9], Bo Wu[1,9], Boya Gao[2], Elizabeth M. Irvin[3], Arijit Ghosh[2], Lillian Eliaz[4], Yuxin Huang[1], Youngho Kwon[1], Clara M. Stiefel[5], Tram Thi Ngoc Nguyen[5], David Zhao[1], Humberto Javier Suarez[1], Tengyang Ni[1], Salvador Alejo[6], O'Taveon Fitzgerald[1], Xuemei Song[7], Sandip Kumar Rath[8], Elizabeth V. Wasmuth[1], David S. Yu[8], Siyuan Zheng[7], Justin Leung[5], Xiaoyu Xue[4], Hong Wang[3], Jae-Hoon Ji[1*], Li Lan[2*], Weixing Zhao[1*]

[1]Department of Biochemistry and Structural Biology, Greehey Children's Cancer Research Institute, University of Texas Health Science Center at San Antonio, TX 78229, USA
[2]Department of Molecular Genetics and Microbiology, School of Medicine, Duke University, Durham, NC 27710, USA
[3]Toxicology Program, Department of Physics, Center for Human Health and the Environment, North Carolina State University, Raleigh, NC 27695, USA
[4]Department of Chemistry & Biochemistry Materials Science, Engineering, and Commercialization Program, Integrated Molecular and Biophysical Chemistry Program, Texas State University, San Marcos, TX 78666, USA
[5]Department of Radiation Oncology, University of Texas Health Science Center at San Antonio, San Antonio, TX 78229, USA
[6]Department of Obstetrics & Gynecology, University of Texas Health Science Center at San Antonio, San Antonio, TX 78229, USA
[7]Department of Population Health Sciences, University of Texas Health Science Center at San Antonio, San Antonio, TX 78229, USA.
[8]Department of Radiation Oncology and Winship Cancer Institute, Emory University, Atlanta, GA 30322, USA
[9]These authors contributed equally to this work
*Corresponding authors. Email: jij@uthscsa.edu; li.lan@duke.edu; zhaow2@uthscsa.edu

----------------------- Includes:
Supplementary Figures 1 through 12
Supplementary Table 1

Supplementary figures and tables

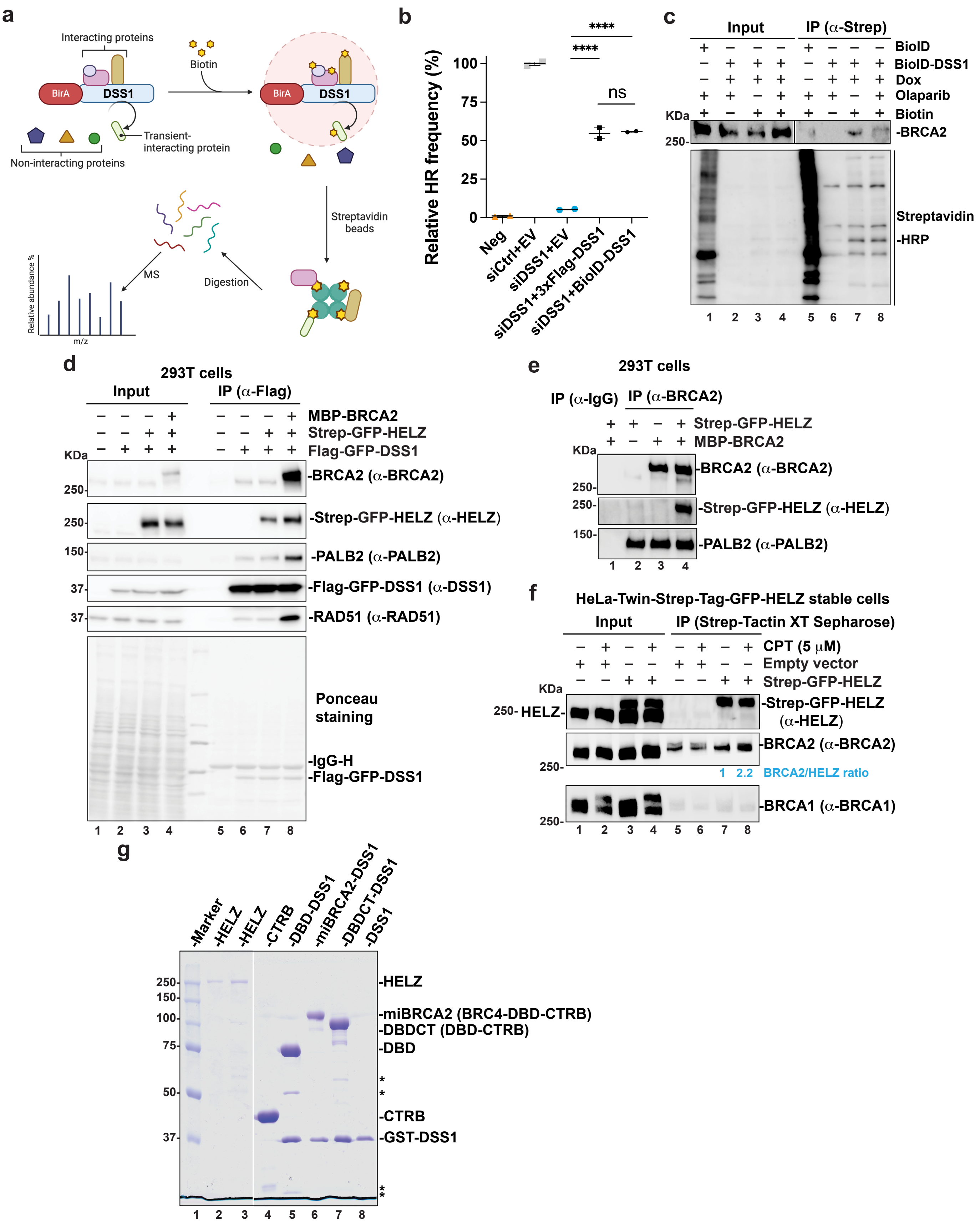

Supplementary Fig. 1 Identifying the interaction between HELZ and BRCA2.

a. Schematic of the proximity-dependent biotinylation (PDB) assay using BioID-DSS1 (Created in BioRender. Zhao, W. (2026) https://BioRender.com/g34v313). In this assay, BirA is fused in-frame to DSS1 and stably expressed in HeLa cells. BirA utilizes biotin to catalyze the formation of activated biotin, which covalently tags lysine residues of proximal proteins. Following cell lysis, biotinylated proteins are affinity-purified using streptavidin-coated beads, digested into peptides by proteolysis, and analyzed by LC-MS/MS for protein identification.
b. Quantification of HR assay results from DR-GFP-U2OS cells upon transfection with siRNA against DSS1 and control (siCtrl) along with transient expressing EV, 3xFlag-DSS1 and BioID-DSS1. Error bars, SD (n=2 independent experiments). Symbol: EV, empty vector. ns, not significant, ****, P ≤ 0.0001(two-sided Student's t-test). Source data are provided as a Source Data File.
c. Western blot analysis to assess enriched biotinylated proteins captured by streptavidin-coated beads before the beads were processed for mass spectrometry. Anti-BRCA2 antibody and anti-streptavidin conjugated HRP were used for probing. Source data are provided as a Source Data File.
d. Anti-Flag beads pulldown Flag-DSS1 in 293T with the transient transfection of MBP-BRCA2, GFP-Strep-HELZ and Flag-DSS1. Anti-BRCA2, HELZ, PALB2, Flag (for DSS1) and RAD51antibodies were used for the Western Blot. Source data are provided as a Source Data File.
e. IP by anti-BRCA2 or mouse IgG in 293T cells with the transient transfection of MBP-BRCA2, GFP-Strep-HELZ. Anti-BRCA2, HELZ, PALB2 antibodies were used for the Western Blot. Source data are provided as a Source Data File.
f. Streptavidin-coated beads were used to pull down GFP-Strep-HELZ in HeLa-HELZ stable cells with CPT (5uM) for 4h. Anti-BRCA2, HELZ, BRCA1 antibodies were used for the Western Blot. The ratio of HELZ to BRCA2 from the IP experiment is shown in cyan. Source data are provided as a Source Data File.
g. SDS-PAGE of purified HELZ, miBRCA2, and various functional domains of miBRCA2 shown in Fig. 1c. Source data are provided as a Source Data File.

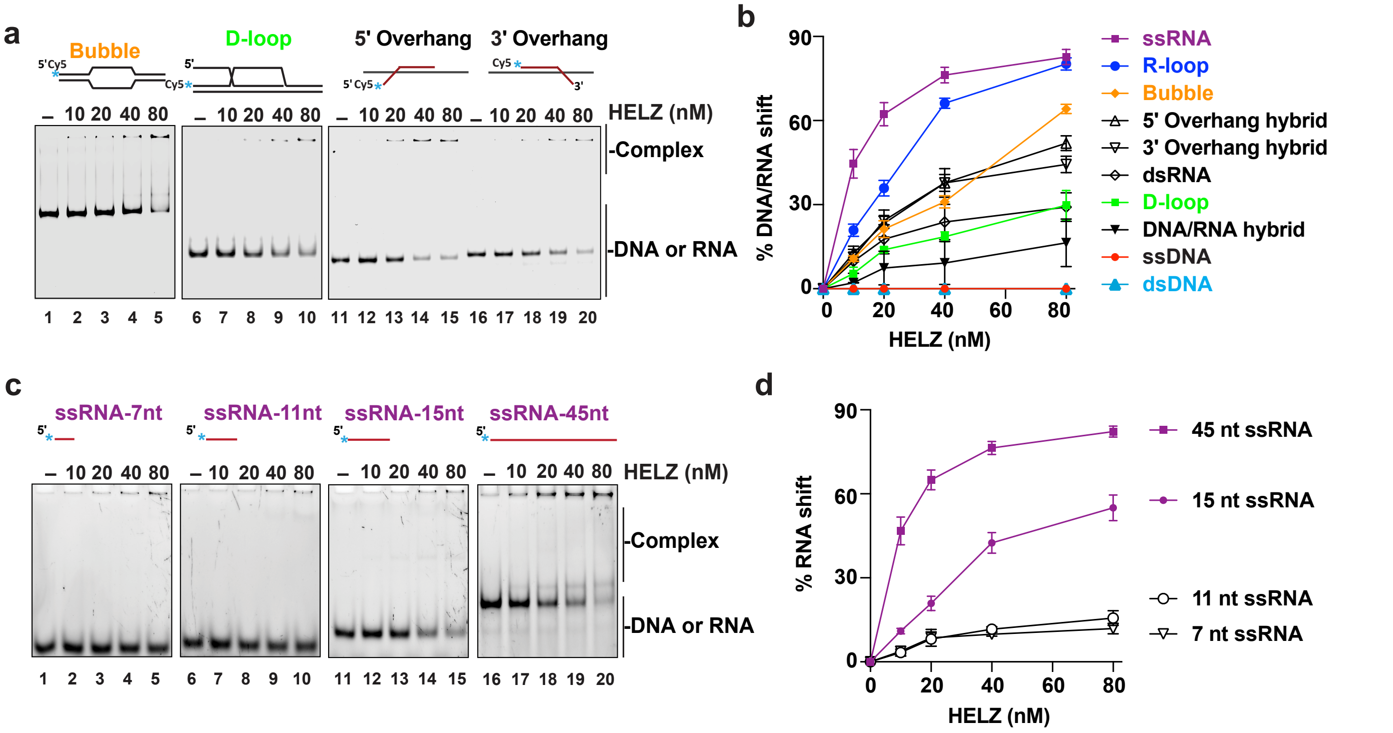

Supplementary Fig. 2 HELZ prefers to bind ssRNA and R-loop.

a. Binding of bubble, D-loop, RNA/DNA hybrid with 5′-RNA overhang, and RNA/DNA hybrid with 3′-RNA overhang by HELZ as examined by EMSA. Source data are provided as a Source Data File.

b. Quantification of the EMSA binding results of a and Fig. 2a. The data represents the mean ± SEM of at least two independent experiments (Bubble, n=3; R-loop, n=6; D-loop, n=2; 5' Overhang, n=5; 3' Overhang n=3; ssRNA, n=3; dsDNA, n=3; ssDNA, n=3; dsRNA, n=3; dsRNA, n=3; RNA/DNA, n=2). Source data are provided as a Source Data File.

c. Binding of 7 nt, 11nt, 15 nt and 45 nt ssRNA by HELZ as examined by EMSA. Source data are provided as a Source Data File.

d. Quantification of the EMSA binding results of c. The data represents the mean ± SEM of three independent experiments. Source data are provided as a Source Data File.

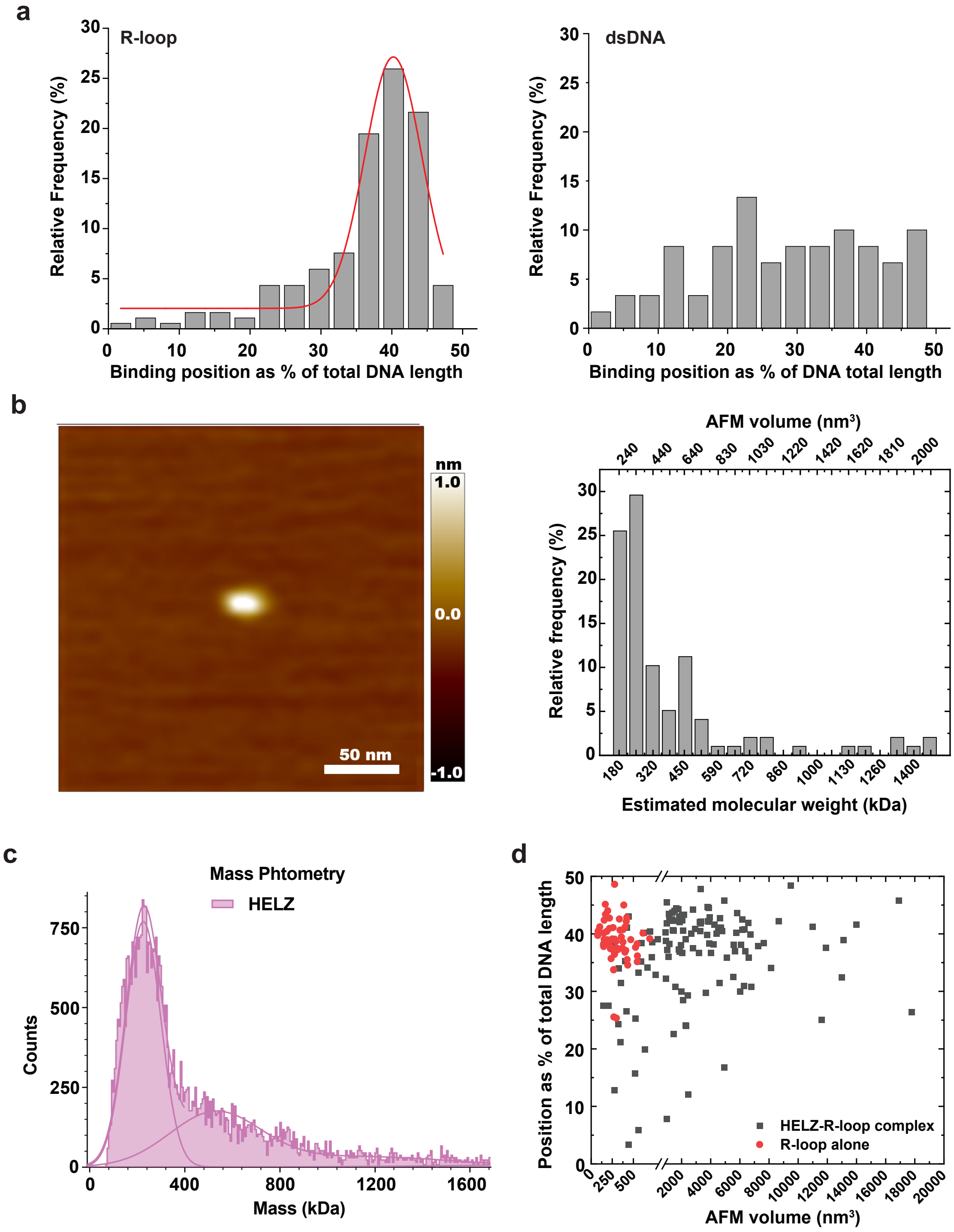

Supplementary Fig. 3 AFM and mass photometry analysis of HELZ and its binding to R-loop and dsDNA.
a. Distributions of HELZ binding positions along the R-loop DNA (n=185 events of one experiment, left) and control dsDNA (n=64 events of one experiment, right). Structures on the R-loop DNA with AFM heights greater than the height of the R-loop alone (1.15 nm) were selected as R-loop-HELZ complexes. HELZ binding position was measured as the distance to the nearest DNA end along the linear DNA and was compared on R-loop DNA and control dsDNA as show in Fig.2c. The percentages of HELZ complexes binding at the 38-42% region are 65.8% and 20.3% on the R-loop DNA and control DNA, respectively. Source data are provided as a Source Data File.
b. The represent AFM image of HELZ monomer in the absence of DNA (left) (n=3 independent experiments). Distribution of estimated molecular weight of HELZ proteins (n=98 events of one experiment, right) shows HELZ primarily exists as a monomer (~200 kDa) in the absence of DNA. The molecular weight was estimated based on measured AFM volume using volume (nm^3) = 1.45 x molecular weight (kDa)-21.59, which was derived through previous calibration using proteins with different molecular weights. Source data are provided as a Source Data File.
c. The represent mass photometry profile of HELZ of three independent experiments. Source data are provided as a Source Data File.
d. Comparison of the AFM volume (nm^3) of HELZ-R-loop complexes (n=127 events of one experiment) to the R-loop alone (n=60 events of one experiment) shows multiple HELZ proteins simultaneously bind to the R-loop site. Source data are provided as a Source Data File.

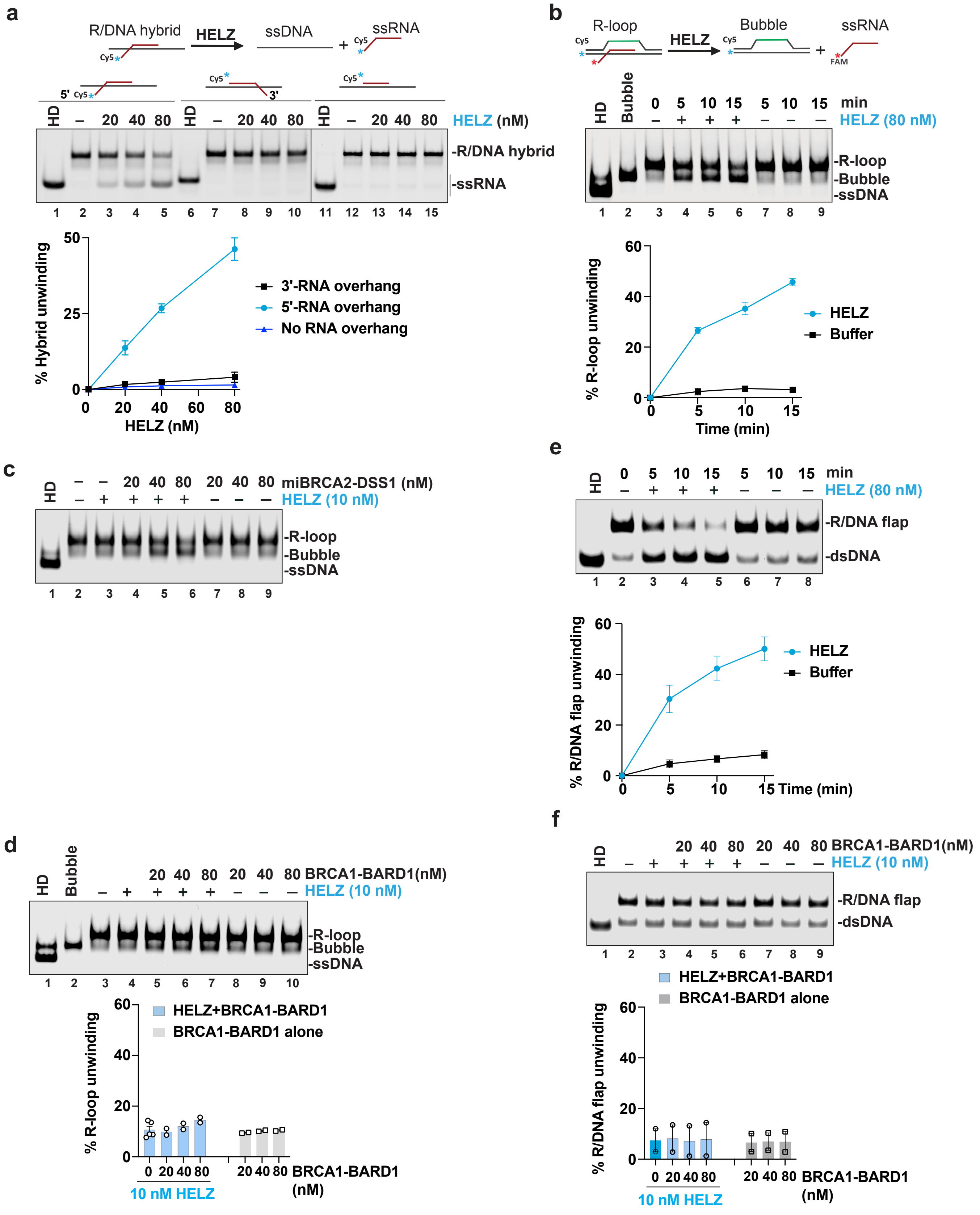

Supplementary Fig. 4 HELZ prefers unwinding RNA-DNA hybrid with 5′-RNA overhang and BRCA2 enhances HELZ unwinding activity

a. Schematic of the RNA/DNA hybrid unwinding assay by HELZ (top). Native PAGE gel showing representative RNA/DNA hybrid (5′-RNA overhang, 3′-RNA overhang, and no RNA overhang) unwinding by HELZ (20, 40, and 80 nM) at 15 min (middle). Quantification of RNA/DNA hybrid unwinding experiments by HELZ (bottom). The data represent the mean ± SEM of two independent experiments. HD: Heat-Denatured. Source data are provided as a Source Data File.
b. Schematic of the R-loop unwinding assay by HELZ (top). PAGE gel showing representative R-loop unwinding by HELZ (80 nM) at 5, 10, and 15 mins (middle), monitored by the Cy5 label on the bubble DNA. Quantification of R-loop unwinding experiments by HELZ at 5, 10, and 15 mins (bottom), in comparison to buffer only. The data represent the mean ± SEM of two independent experiments. Source data are provided as a Source Data File.
c. Native PAGE gel showing representative R-loop unwinding activity by HELZ (10 nM) in the presence of miBRCA2-DSS1 (20, 40, and 80 nM) and by miBRCA2-DSS1 alone at 15 min. The representative image of three independent experiments was provided. Quantification of unwinding is shown in Fig. 2g. Source data are provided as a Source Data File.
d. Native PAGE gel showing representative R-loop unwinding activity of HELZ (10 nM) in the presence of BRCA1-BARD1 (20, 40, and 80 nM) and of BRCA1-BARD1 alone at 15 min (top). Quantification of R-loop unwinding efficiency of HELZ stimulated by BRCA1-BARD1. The data represent the mean ± SEM of two independent experiments. Source data are provided as a Source Data File.
e. Native PAGE gel showing representative RNA-DNA flap unwinding by HELZ (80 nM) at 5, 10, and 15 mins (top). Quantification of RNA-DNA flap unwinding experiments by HELZ at 5, 10, and 15 mins in comparison to buffer alone (bottom). The data represent the mean ± SEM of three independent experiments. Source data are provided as a Source Data File.
f. Native PAGE gel showing representative RNA-DNA flap unwinding activity of HELZ (10 nM) in the presence of BRCA1-BARD1 (20, 40, and 80 nM) and by BRCA1-BARD1 alone at 15 min (top). Quantification of RNA-DNA flap unwinding efficiency of HELZ in the presence of BRCA1-BARD1. The data represent the mean ± SEM of two independent experiments. Source data are provided as a Source Data File.

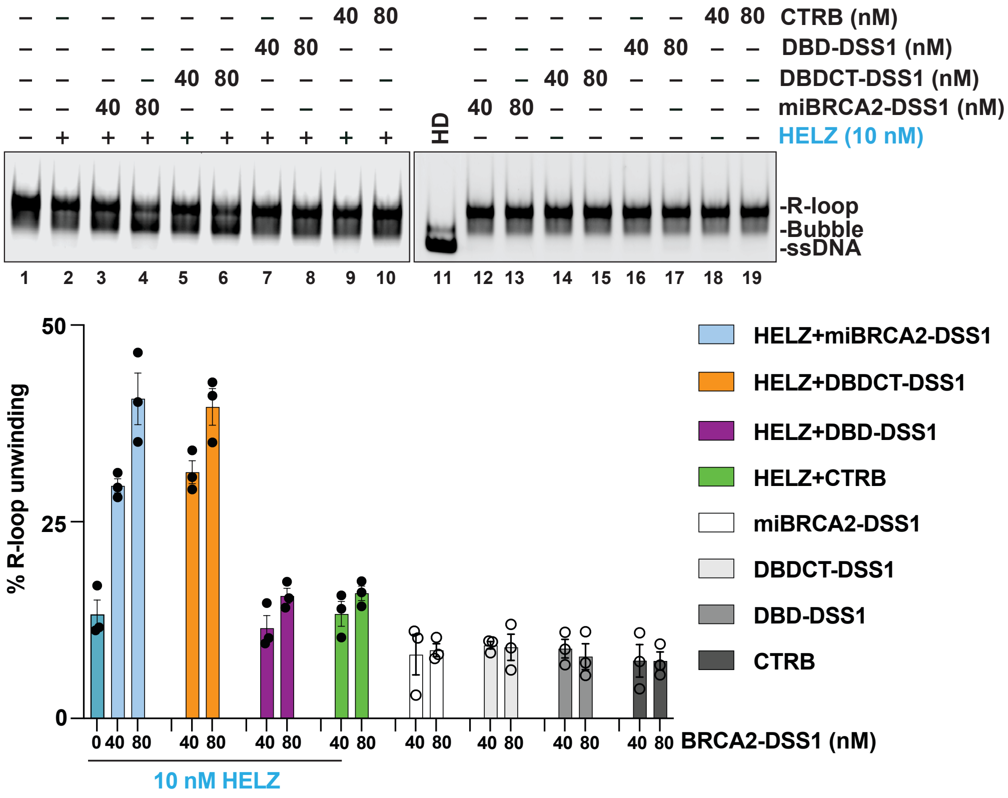

Supplementary Fig. 5 Domain requirement for miBRCA2 to enhance HELZ unwinding activity
Native PAGE gel showing representative R-loop unwinding activity of HELZ (10 nM) in the presence of 40 and 80 nM of miBRCA2-DSS1, DBDCT-DSS1, DBD-DSS1 and CTRB (top). Quantification of R-loop unwinding experiments by HELZ with miBRCA2-DSS1, DBDCTRB-DSS1, DBD-DSS1 and CTRB (bottom). The data represent the mean ± SEM of three independent experiments. HD: Heat-Denatured. Source data are provided as a Source Data File.

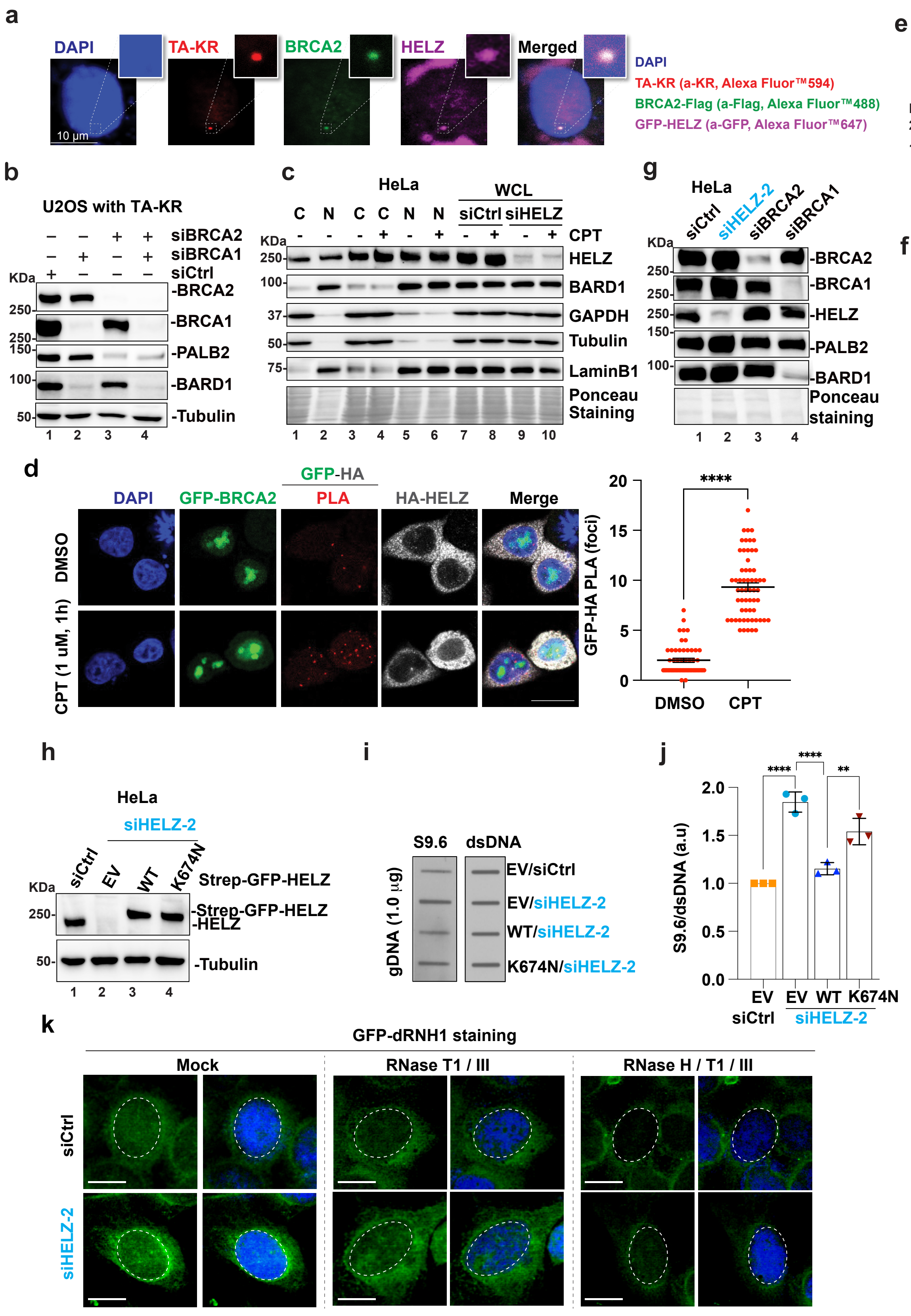

Supplementary Fig. 6 HELZ depletion induced R-loop accumulation.

a. U2OS-TRE cells transfected with pBROAD3 TA-KR and the expression vector of GFP-HELZ and MBP-BRCA2-Flag were light-activated and recovered 20 min before fixation. Representative images of GFP and Flag foci at sites of TA-KR from three independent experiments were shown. Flag-BRCA2 (Alexa Fluor™ 488) and HA-HELZ (Alexa Fluor™ 647) detected with anti-Flag and anti-HA antibodies. DAPI marks nuclei (blue); TA-KR (Alexa Fluor™ 594) marks transcriptionally active DNA damage sites. Scale bar: 10 µm
b. Western blot analysis to assess the knockdown efficiency of BRCA1 and BRCA2 using siRNA in U2OS cells employing the TA-KR system for Fig. 3d. Antibodies against BRCA2, BRCA1, PALB2, BARD1, and Tubulin were used for probing. Source data are provided as a Source Data File.
c. Western blot to verify the nuclear localization of endogenous HELZ in HeLa cells. The cytoplasmic and nuclear fractions were analyzed. Lamin B1 and BARD1 mark nuclear fraction; Tubulin and GAPDH mark cytoplasmic fraction. Ponceau S staining was used as a loading control. Source data are provided as a Source Data File.
d. Representative micrographs of PLA foci (red) showing colocalization between BRCA2 (α-GFP) and HELZ (α-HA) in the nuclei of HeLa cells transient expressing BRCA2-GFP and HA-HELZ after the treatment of DMSO or CPT (1µM; 1 h) (left). Blue: DAPI. Green: BRCA2-GFP. Grey: HA-HELZ. Scale bar: 10 µm. Average number (±SEM) of PLA foci from 50-60 nuclei (53 for DMSO and 59 for CPT) from one experiment were plotted (right). Statistical analysis was done with the two-sided Student's t-test, ****, $P= 4.61199E-29 \leq 0.0001$. Source data are provided as a Source Data File.
e. Western blot analysis to assess the knockdown efficiency of HELZ using siRNA in U2OS cells employing the TA-KR system for Fig. 3e and 3f. Antibodies against HELZ, PALB2 and H3 were used for probing. Source data are provided as a Source Data File.
f, g. Western blot analysis to assess the knockdown efficiency of HELZ with two different siRNA in HeLa cells (f) and to check the off-target effect on BRCA1, BRCA2, PALB2 and BARD1 of siHELZ-2 (g), where siBRCA1 and siBRCA2 as the controls. Antibodies against HELZ, BRCA2, BRCA1, PALB2, BARD1 and Tubulin were used for probing. Source data are provided as a Source Data File.
h. Western blot analysis to evaluate the knockdown efficiency of HELZ and the ectopic expression of siRNA-resistant HELZ ^{WT} and HELZ^{K674N} for the S9.6 slot blot presented in i. Symbol: EV, empty vector. Source data are provided as a Source Data File.
i. Representative RNA/DNA hybrid slot blot of genomic DNA from HeLa cells with the HELZ knockdown and ectopic expression of HELZ siRNA resistant HELZ ^{WT} and HELZ^{K674N} from three independent experiments. dsDNA antibody was used for control input of each sample. dsDNA antibody as a loading control. Source data are provided as a Source Data File.
j. Quantification (mean ± SEM) of enrichment of R-loop (detected by S9.6 antibody) into genomic DNA of HeLa cells with the HELZ knockdown and ectopic expression of HELZ siRNA resistant HELZ ^{WT} and HELZ^{K674N} for i. au: arbitrary unit. Statistical analysis was done from three independent experiments with the two-sided Student's t-test, ** $p \leq 0.01$, ****$p \leq 0.0001$. Source data are provided as a Source Data File.
k. Representative confocal images from three independent experiments showing accumulation of RNA-DNA hybrids by GFP-dRNH1 (green fluorescence) staining in U2OS cells transfected with control or HELZ siRNA in Fig.3f. Nuclei were stained with DAPI (blue). After fixation, the cells were treated with the following enzymes: no treatment (Mock); RNase T1 and RNase III combined (T1/III); RNase H, RNase T1, and RNase III combined (H/T1/III). Scale bars: 5 µm.

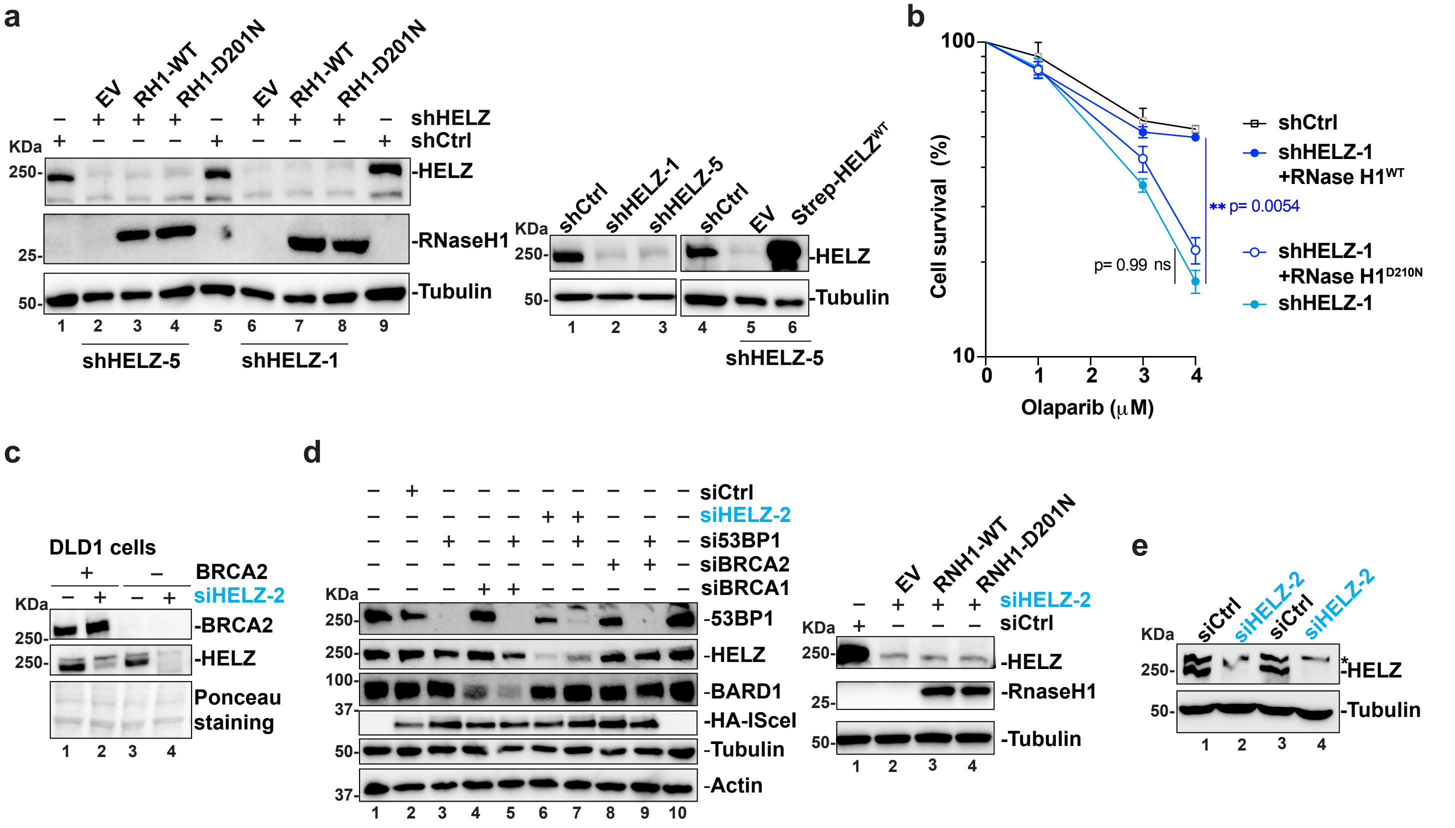

Supplementary Fig. 7 R-loop accumulation by HELZ depletion promotes HR and cell survival.

a. Western blot analysis to evaluate the knockdown efficiency of HELZ and the ectopic expression of HELZ, RNase H1^{WT} and RNase H1^{D210N} in HeLa-shHELZ-5 and -shHELZ-1 stable cell lines for Fig. 4b and b. Anti-HELZ, HA (RNase H1) and Tubulin antibodies were used for the probing. Source data are provided as a Source Data File.

b. Clonogenic survival of HeLa cells depleted with shHELZ-1 and ectopic expression of RNase H1^{WT} and RNase H1^{D210N} upon treatment with olaparib, Error bars, SEM (n=3 independent experiments). ns, not significant; **, P ≤ 0.01 (two-way ANOVA). Source data are provided as a Source Data File.

c. Western blot analysis to assess the knockdown efficiency of HELZ using siRNA in DLD1 cells for Fig. 4c. Antibodies against BRCA2, HELZ were used for probing. Ponceau staining was used for loading control. Source data are provided as a Source Data File.

d. Western blot analysis to assess the knockdown efficiency of HELZ, BRCA1, BRCA2 and 53BP1 using siRNA in U2OS-DRGFP cells for Fig. 4d. Antibodies against 53BP1, HELZ, BARD1, HA (I-*Sce*I and RNase H1), BARD1, Tubulin, Actin were used for probing. Tubulin blot and/or Ponceau staining blot were used for loading control. Source data are provided as a Source Data File.

e. Western blot analysis to assess the knockdown efficiency of HELZ using siRNA in U2OS-Tet-DR-GFP cells for Fig. 4e. Antibodies against HELZ and Tubulin were used for probing. *: non-specific band. Source data are provided as a Source Data File.

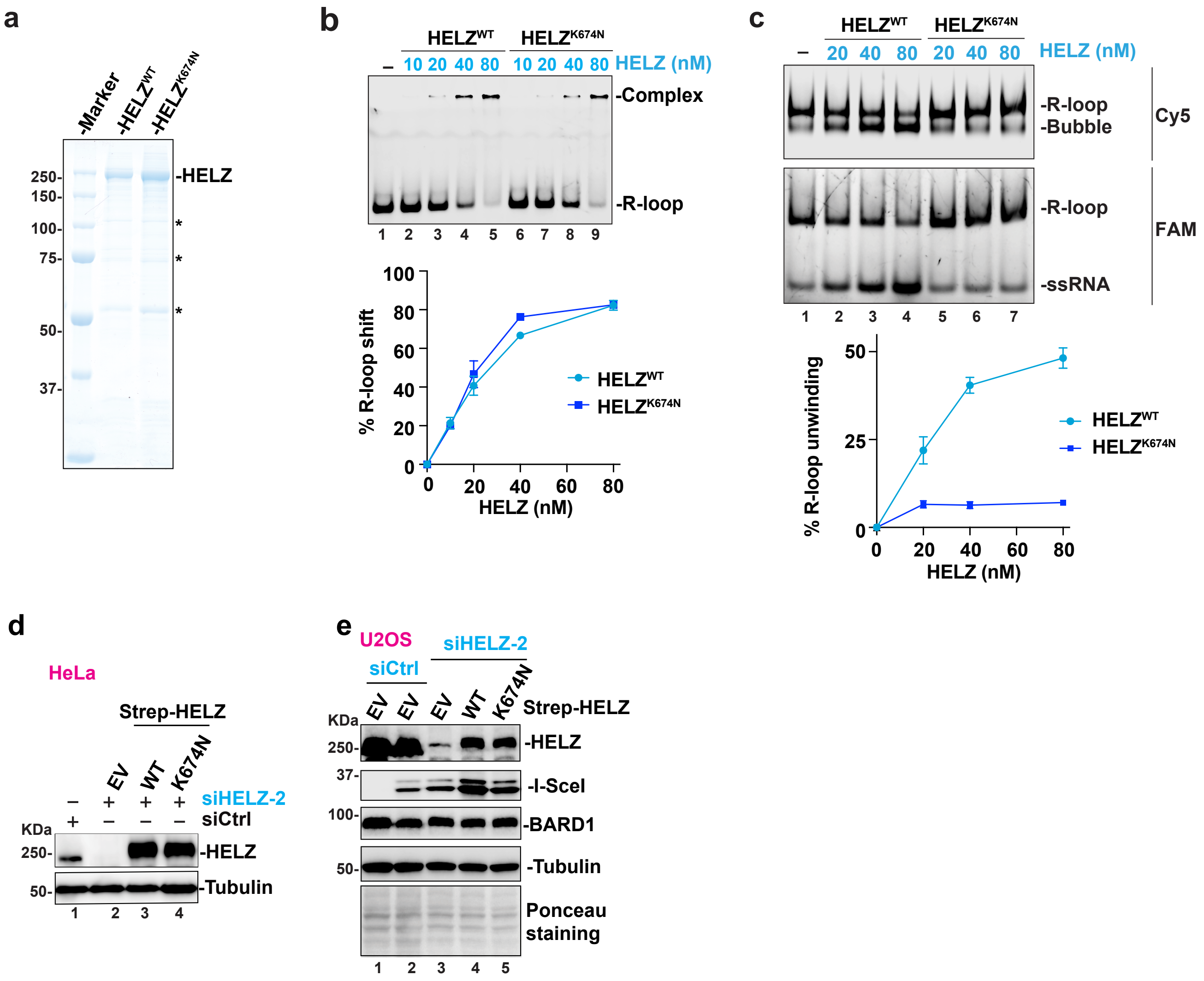

Supplementary Fig. 8 Residue K674 is the key site for HELZ unwinding activity.

a. SDS-PAGE of purified HELZ^{WT} and HELZ^{K674N} mutant. *: non-specific band. Source data are provided as a Source Data File.

b. Binding of R-loop by HELZ^{WT} and HELZ^{K674N} as examined by EMSA (top). Quantification of the EMSA results (bottom). The data represent the mean ± SEM of three independent experiments. Source data are provided as a Source Data File.

c. R-loop unwinding activity of HELZ^{WT} and HELZ^{K674N} mutant at 20, 40, and 80 nM (top). Quantification of the RNA-DNA flap unwinding activity (bottom). The data represent the mean ± SEM of three independent experiments. Source data are provided as a Source Data File.

d. Western blot analysis to evaluate the knockdown efficiency of HELZ and the ectopic expression of siRNA-resistant HELZ^{WT} and HELZ^{K674N} under the HELZ siRNA treatment for Fig. 5c. Antibodies against HELZ and Tubulin were used for probing. Tubulin blot served as loading control. Source data are provided as a Source Data File.

e. Western blot analysis to evaluate the knockdown efficiency of HELZ and the ectopic expression of siRNA-resistant HELZ^{WT} and HELZ^{K674N} under the HELZ siRNA treatment for Fig. 5d. Antibodies against HELZ, BARD1, HA (I-*Sce*I), BARD1 and Tubulin were used for probing. Tubulin blot and Ponceau staining served as loading controls. Source data are provided as a Source Data File.

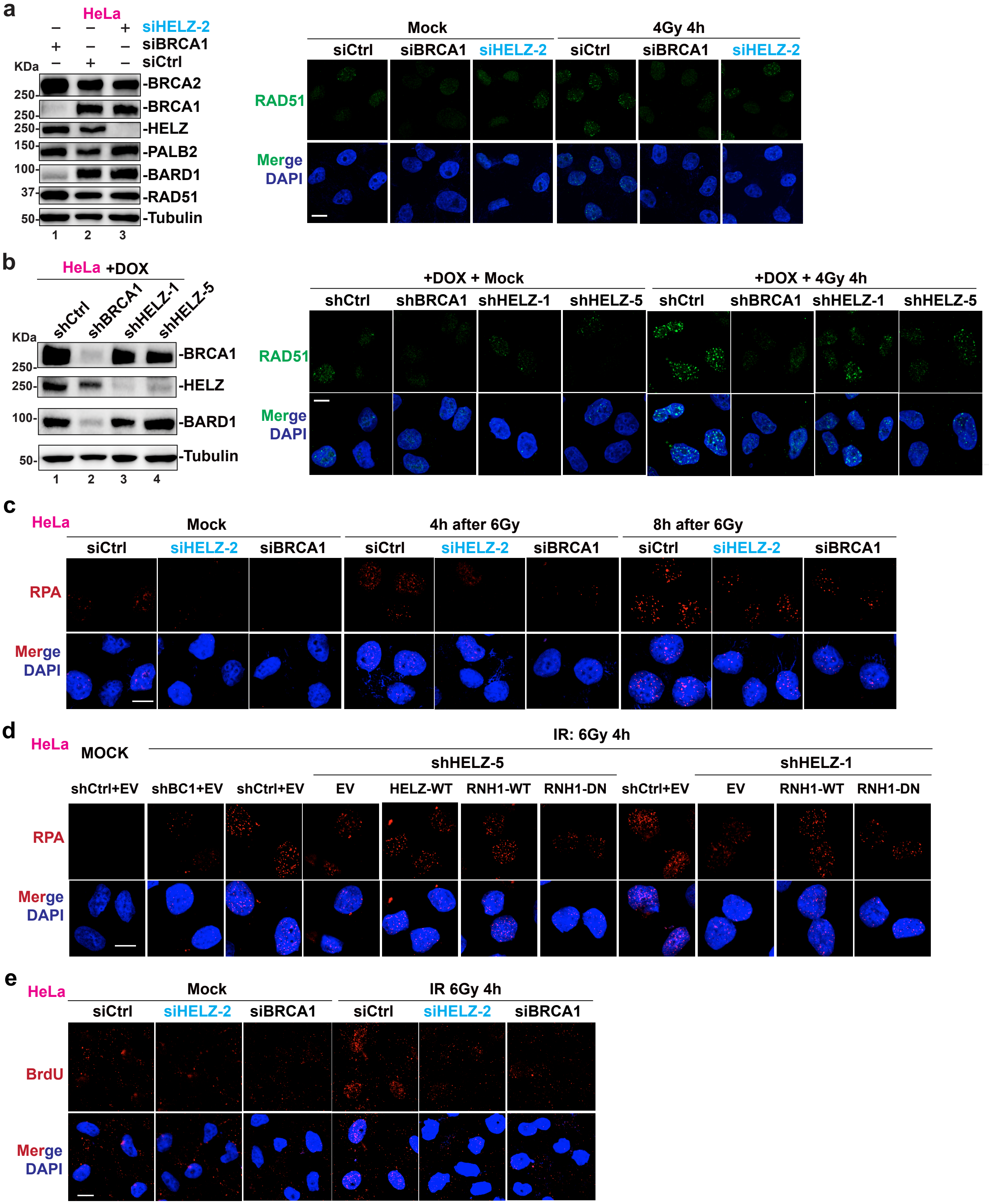

Supplementary Fig. 9 HELZ depletion reduces the focus formation of RAD51, RPA and BrdU in HeLa cells.

a. Western blot analysis to assess the knockdown efficiency of HELZ and BRCA1 using siRNA in HeLa cells for Fig. 5a (left). Antibodies against BRCA1, HELZ, BARD1, BRCA2, PALB2, RAD51 and Tubulin were used for probing. Representative images of RAD51 foci (green) in HeLa nuclei at 4hr after exposure to 4 Gy X-rays or sham irradiation (right). DAPI: blue. Scale bar:10 μm. Source data are provided as a Source Data File.
b. Western blot analysis to assess the knockdown efficiency of HELZ and BRCA1 using shRNA in HeLa cells for Fig. 5b (left). Antibodies against BRCA1, HELZ, BARD1 and Tubulin were used for probing. Representative images of RAD51 foci (green) in HeLa nuclei at 4hr after exposure to 4 Gy X-rays or sham irradiation (right). DAPI: blue. Scale bar:10 μm. Source data are provided as a Source Data File.
c. Representative images of RPA foci (red) for Fig. 6c in HeLa nuclei at 4hr and 8h after exposure to 6 Gy X-rays or sham irradiation. DAPI: blue. Scale bar:10 μm.
d. Representative images of RPA foci (red) for Fig. 6d in shHELZ-1 and shHELZ-5 HeLa nuclei at 4hr after exposure to 6 Gy X-rays or sham irradiation. DAPI: blue. Scale bar:10 μm.
e. Representative images of BrdU foci (red) for Fig. 6e in HeLa nuclei at 4hr after exposure to 6 Gy X-rays or sham irradiation. DAPI: blue. Scale bar:10 μm.

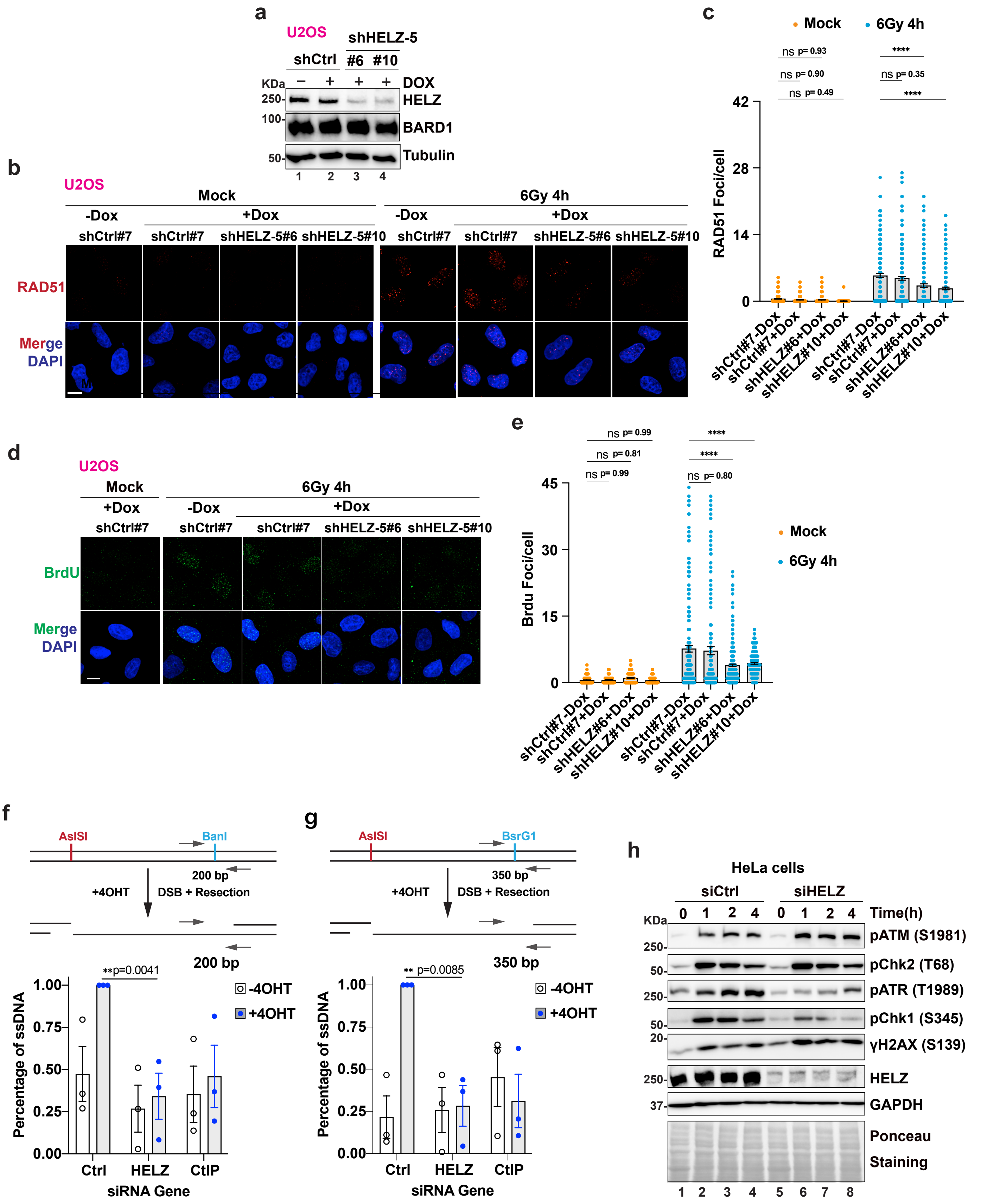

Supplementary Fig. 10 HELZ depletion reduces the focus formation of RAD51/BrdU and ssDNA production in U2OS cells.

a. Western blot analysis to assess the knockdown efficiency of HELZ using shRNA in U2OS cells (two different single clones) by the treatment of doxycycline. Antibodies against HELZ, BARD1 and Tubulin antibodies were used for probing. Source data are provided as a Source Data File.
b. Representative images of RAD51 foci (red) (n=3 three independent experiments) in U2OS nuclei at 4hr after exposure to 6 Gy X-rays or sham irradiation. DAPI: blue. Scale bar:10 μm.
c. Quantification of b. Mean values ± SEM for at least 100 cells from one experiment were counted. ns, not significant; ****, $P \leq 0.0001$(two-way ANOVA). Source data are provided as a Source Data File.
d. Representative images of BrdU foci (green) (n=3 three independent experiments) in U2OS nuclei at 4hr after exposure to 6 Gy X-rays or sham irradiation. DAPI: blue. Scale bar:10 μm.
e. Quantification of d. Mean values ± SEM for at least 100 cells from one experiment were counted. ns, not significant; ****, $P \leq 0.0001$(two-way ANOVA). Source data are provided as a Source Data File.
f. DIvA (AsiSI-ER-U2OS) cells were treated with 300 nM 4-OHT for 4 h or mock, genomic DNA (gDNA) was extracted and digested or mock digested with BanI overnight. DNA end resection adjacent to DSB was measured by qPCR from three independent experiments. **, $P \leq 0.01$ (two-sided Student's t-test).
g. DIvA (AsiSI-ER-U2OS) cells were treated with 300 nM 4-OHT for 4 h or mock, gDNA was extracted and digested or mock digested with BsrG1 overnight. DNA end resection adjacent to DSB was measured by qPCR from three independent experiments. **, $P \leq 0.01$ (two-sided Student's t-test).
h. Western blot analysis to assess DNA damage response signaling upon HELZ depletion in HeLa cells. Antibodies against pATM (S1981), pChk2 (T68), pATR(T1989), pChk1(S345), γH2AX (S139) and HELZ were used for probing. GAPDH blot and Ponceau staining served as loading controls. Data are representative of n=3 independent experiments with similar results. Source data are provided as a Source Data File.

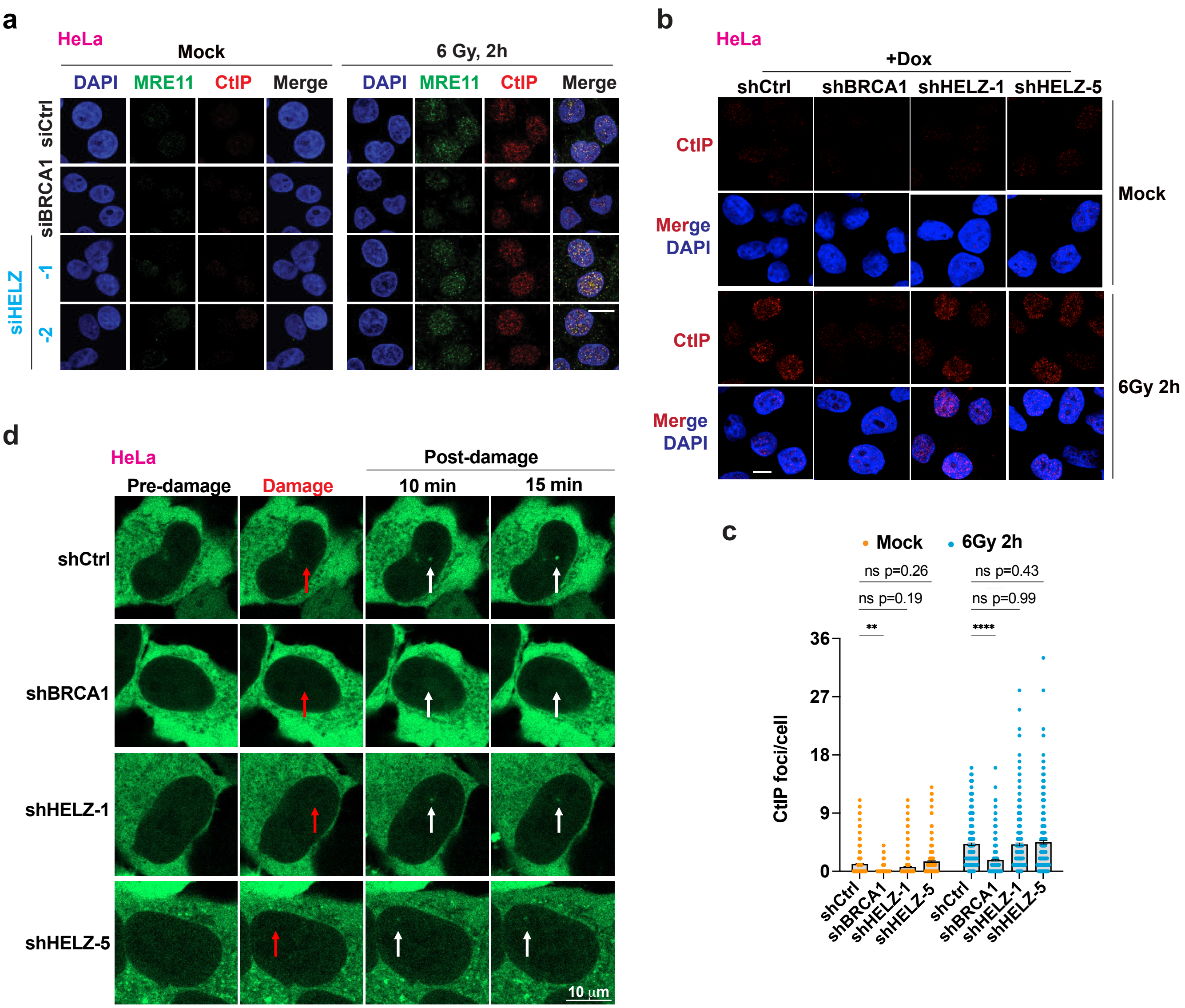

Supplementary Fig. 11 The effect of HELZ depletion on the recruitment of short and long DNA end resection factors.

a. Representative images of MRE11 foci (green) and CtIP Foci (red) in HeLa nuclei at 2hr after HELZ siRNA (siHELZ-1 and siHELZ-2) knockdown after exposure to 6 Gy X-rays or sham irradiation from three independent experiments (Quantification shown in Fig. 5f, g). DAPI: blue. Scale bar:10 μm.

b. Representative images of CtIP foci (red) (n=3 three independent experiments) in HeLa-shHELZ and -shBRCA1 nuclei at 2hr after exposure to 6 Gy X-rays or sham irradiation. DAPI: blue. Scale bar:10 μm.

c. Quantification of b. Mean values ± SEM for at least 100 cells from one experiment were counted. ns, not significant; **, P<0.01, ****, P<0.0001 (Two-way ANOVA). Source data are provided as a Source Data File.

d. Representative images of GFP-DNA2 recruitment (n=3 three independent experiments) to sites of laser-induced micro-irradiation (405 nm, 100% power) in HeLa cells expressing shRNA against HELZ or BRCA1 as indicated. The damaged area is indicated with a red arrow; the corresponding region-of-interest is indicated with a white arrow. Scale bar:10 μm.

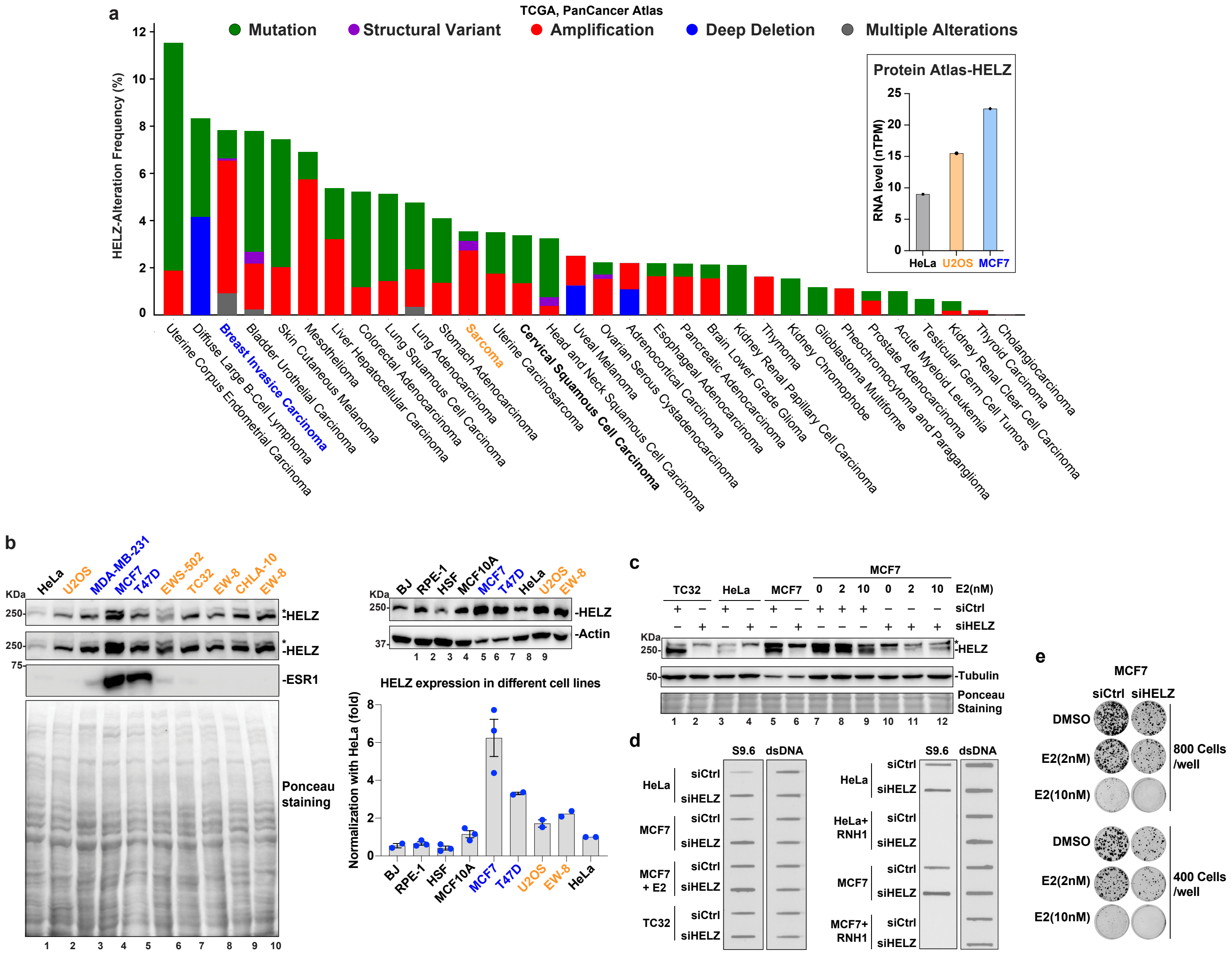

Supplementary Fig. 12 HELZ is upregulated in R-loop stressed cancer cells.

a. TCGA data indicating the amplification status of HELZ across various cancer types.

b. Western blot to check HELZ expression in various cancer and normal cells. Anti-HELZ and ESR1 antibody were used for probing, the Ponceau Staining was used as the loading control. *, non-specific band. HELZ expression levels were quantified and normalized to Actin. The representative image of three independent experiments was provided. Source data are provided as a Source Data File.

c. Western blot analysis to evaluate the knockdown efficiency of HELZ in the samples used in the d-g. Antibodies against HELZ and Tubulin were used for probing. Ponceau Staining was used as the loading control. *: non-specific band. Source data are provided as a Source Data File.

d. Representative RNA/DNA hybrid slot blot of genomic DNA from HeLa (-/+ RNase H), MCF (-/+ E2; -/+ RNase H), and TC32 cells with the HELZ knockdown and the treatment of estrogen from three independent experiments. E2: treatment of estrogen. dsDNA antibody was used for control input of each sample. Source data are provided as a Source Data File.

e. Representative images of clonogenic survival in MCF7 cells upon (0, 2 and 10 nM) E2 and siHELZ treatment. The representative image of three independent experiments was provided.

Supplementary table 1: Oligos used in this study

Name	Sequence
Oligo 1	5'/Cy5/-TTATATCCTTTACTTTGAATTCTATGTTTAACCTTTTACTTATTTTGTATTAGCC GGATCCTTATTTCAATTATGTTCAT-3'
Oligo 2	5'-ATGAACATAATTGAAATAAGGATCCACTCTACATGCTCACACACTCGAACTCATG ATAGAATTCAAAGTAAAGGATATAA-3'
Oligo 3	5'/FAM/-CUAGUGGAAGCGAGUCAUGAGUUCGAGUGUGUGAGCAUGUAGAGU-3'
Oligo 4	5'-CTAGTGGAAGCGAGTCATGAGTTCGAGTGTGTGAGCATGTAGAGT-3'
Oligo 5	5'-ACGCTGCCGAATTCTACCAGTGCCTTGCTAGGACATCTTTGCCCACCTGCA GGTTCACCC-3'
Oligo 6	5'-GGGTGAACCTGCAGGTGGGCAAAGATGTCCCAGCAAGGCACTGGTAGAATTCGG CAGCGT/800CW/--3'
Oligo 7	5'-GGGUGAACCUGCAGGUGGGCAAAGAUGUCC-3'
Oligo 8	5'-GTCACTTGATAAGAGGTCATTTGAATTCATGGCTTAGAGCTTAATTGCTGAATCTG GTGCTGGGATCCAACATGTTTTAAATATGCAATG-3'
Oligo 9	5'/Cy5/-UCGUAGCUCGGGAGUGCACCAGAUUCAGCAAUUAAGCUCUAGCC-3'
Oligo 10	5'/Cy5/-GCACCAGAUUCAGCAAUUAAGCUCUAAGCCGCUGACGGCUCGAUG-3'
Oligo 11	5'/Cy5/-ACTCTACATGCTCACACACTCGAACTCATGACTCGCTTCCACTAG-3'
Oligo 12	5'/Cy5/-GCACCAGAUUCAGCAAUUAAGCUCUAAGCC-3'
Oligo 13	5'-ACUCUACAUGCUCACACACUCGAACUCAUGACUCGCUUCCACUAG-3’
Oligo 14	5'/FAM/-CUAGUGG-3'
Oligo 15	5'/FAM/-CUAGUGGAAGC-3'
Oligo 16	5'/FAM/-CUAGUGGAAGCGAGU-3'
Oligo 17	5'-ACTCTACATGCTCACACACTCGAACTCATG-3'
Oligo 18	5'-GGCTTAGAGCTTAATTGCTGAATCTGGTGC-3'
