# Supplementary material for: HELZ-BRCA2 complex resolves R-loops to drive transcription-coupled homologous recombination
Source: Nat Commun. 2026 Jul 23;17:6969. doi: 10.1038/s41467-026-75088-4 (PMC13396364; doi:10.1038/s41467-026-75088-4)
Supplement: Supplementary file 2 — Reporting Summary [file 41467_2026_75088_MOESM2_ESM.pdf]

nature portfolio

Corresponding author(s): Jae-Hoon Ji, Li Lan, Weixing Zhao

Last updated by author(s): Jan 6, 2026

Reporting Summary

Nature Portfolio wishes to improve the reproducibility of the work that we publish. This form provides structure for consistency and transparency in reporting. For further information on Nature Portfolio policies, see our Editorial Policies and the Editorial Policy Checklist.

Statistics

For all statistical analyses, confirm that the following items are present in the figure legend, table legend, main text, or Methods section.

n/a	Confirmed	
☐	☒	The exact sample size (*n*) for each experimental group/condition, given as a discrete number and unit of measurement
☐	☒	A statement on whether measurements were taken from distinct samples or whether the same sample was measured repeatedly
☐	☒	The statistical test(s) used AND whether they are one- or two-sided<br>*Only common tests should be described solely by name; describe more complex techniques in the Methods section.*
☐	☒	A description of all covariates tested
☐	☒	A description of any assumptions or corrections, such as tests of normality and adjustment for multiple comparisons
☐	☒	A full description of the statistical parameters including central tendency (e.g. means) or other basic estimates (e.g. regression coefficient) AND variation (e.g. standard deviation) or associated estimates of uncertainty (e.g. confidence intervals)
☐	☒	For null hypothesis testing, the test statistic (e.g. F, t, r) with confidence intervals, effect sizes, degrees of freedom and P value noted<br>*Give P values as exact values whenever suitable.*
☒	☐	For Bayesian analysis, information on the choice of priors and Markov chain Monte Carlo settings
☒	☐	For hierarchical and complex designs, identification of the appropriate level for tests and full reporting of outcomes
☒	☐	Estimates of effect sizes (e.g. Cohen's d, Pearson's r), indicating how they were calculated

Our web collection on statistics for biologists contains articles on many of the points above.

Software and code

Policy information about availability of computer code

Data collection	TCGA
Data analysis	R (version 4.4.0) for mass spectrum data analysi; FlowJo software for Flow cytometry analsysis; Prism 9 -GraphPad Software for all the statistic data analysis, BioRender was used to generate part of Figure 2b, Figure 7d, and Supplementary Fig. 1a.

For manuscripts utilizing custom algorithms or software that are central to the research but not yet described in published literature, software must be made available to editors and reviewers. We strongly encourage code deposition in a community repository (e.g. GitHub). See the Nature Portfolio guidelines for submitting code & software for further information.

Data

Policy information about availability of data

All manuscripts must include a data availability statement. This statement should provide the following information, where applicable:

- Accession codes, unique identifiers, or web links for publicly available datasets
- A description of any restrictions on data availability
- For clinical datasets or third party data, please ensure that the statement adheres to our policy

All data is available and provided in the Source Data File-HELZ-NCOMMS-25-55720A, all homemade materials used in this research will be available upon request to the lead correspondence author: Weixing Zhao, zhaow2@uthscsa.edu. The Mass spectrum data generated in this study have been deposited in Project ProteomeXchange with identifier PXD066766 with public access. Project Webpage: https://www.ebi.ac.uk/pride/archive/projects/PXD066766.

Research involving human participants, their data, or biological material

Policy information about studies with human participants or human data. See also policy information about sex, gender (identity/presentation), and sexual orientation and race, ethnicity and racism.

Reporting on sex and gender	N/A
Reporting on race, ethnicity, or other socially relevant groupings	N/A
Population characteristics	N/A
Recruitment	N/A
Ethics oversight	N/A

Note that full information on the approval of the study protocol must also be provided in the manuscript.

Field-specific reporting

Please select the one below that is the best fit for your research. If you are not sure, read the appropriate sections before making your selection.

[x] Life sciences [ ] Behavioural & social sciences [ ] Ecological, evolutionary & environmental sciences

For a reference copy of the document with all sections, see nature.com/documents/nr-reporting-summary-flat.pdf

Life sciences study design

All studies must disclose on these points even when the disclosure is negative.

Sample size	No sample size calculation was performed. We have used the data from at least three independent experiments. 2-3 repeats is usually a good starting place for evaluating the spread of the data. Importantly, the P values results from statistical analyses suggested that the samples sizes in our study are sufficient.
Data exclusions	N/A
Replication	The experimental findings were reliably reproduced and the data are analyzed from at least 2-3 independent experiments.
Randomization	N/A
Blinding	Blinding was not part of the study design. The research involved in molecular analysis of specific proteins and the researchers needed to produce and characterize them and their truncations or domains.

Reporting for specific materials, systems and methods

We require information from authors about some types of materials, experimental systems and methods used in many studies. Here, indicate whether each material, system or method listed is relevant to your study. If you are not sure if a list item applies to your research, read the appropriate section before selecting a response.

Materials & experimental systems

n/a	Involved in the study	
[ ]	[x]	Antibodies
[ ]	[x]	Eukaryotic cell lines
[x]	[ ]	Palaeontology and archaeology
[x]	[ ]	Animals and other organisms
[x]	[ ]	Clinical data
[x]	[ ]	Dual use research of concern
[x]	[ ]	Plants

Methods

n/a	Involved in the study	
[x]	[ ]	ChIP-seq
[ ]	[x]	Flow cytometry
[x]	[ ]	MRI-based neuroimaging

Antibodies

Antibodies used	HELZ (home-made, a gift from David Yu's lab), HA (3724S, Cell Signaling; 1:1000), BRCA1(SC6954, Santa Cruz; 1:500), BRCA2 (EMD Millipore, OP95-100UG; 1:1000), PALB2(home-made, a gift from Bing Xia's lab), DSS1 (SC28848, Santa Cruz; 1:500), Tubulin (2128S,

	Cell Signaling; 1:2000), GAPDH (2118S, Cell Signaling; 1:15000), Estrogen Receptor α (SC8002, Santa Cruz: 1:500), anti-Streptavidin-HRP (NC9705430, Jackson Immuno Research Labs; 1:3000), Flag M2-HRP (Sigma, A8592; 1:3000), p-ATM (S1981) (13050, Cell Signaling; 1:1000), p-Chk2 (T68) (2197, Cell Signaling; 1:1000), p-ATR (T1989) (30632, Cell Signaling; 1:1000), p-Chk1 (S345) (2348, Cell Signaling; 1:1000), and γH2AX (S139) (9718, Cell Signaling; 1:1000) for WB. RAD51(8875S, Cell Signaling; 1:500), RPA (MABE285, Millipore, 1:500), BrdU (347580, BD Biosciences; 1:20), BRCA1 (SC6954, Santa Cruz; 1:500), MRE11(NB100-473, Novus; 1:500) and CtIP (PA5-84133, ThermoScientific; 61141, Active Motif; 1:500) for IF.
Validation	Specificity validation data from the manufacturer or from experiments using specific siRNA.

Eukaryotic cell lines

Policy information about cell lines and Sex and Gender in Research

Cell line source(s)	HEK293T (ATCC); HeLa (ATCC); U2OS-DR-GFP (gift from Jeremy Stark); U2OS-TRE and U2OS-Tet-on DR-GFP cells(U2OS cells were purchased from ATCC and integrated with TRE repeats and tet-DR-GFP cassette); MCF7 , T47D and MDA-MB-231(gift from Dr. Ratna Vadlamudi and Suryavathi Viswanadhapalli); TC32, EwS502, EwS8 and CHLA10(gift from Dr. Alex Bishop).
Authentication	Short Tandem Repeat (STR) profiling were preformed by ATCC. The gifted cell lines have not been authenticated by us.
Mycoplasma contamination	Mycoplasma contamination were tested by Bionique testing labs (http://www.bionique.com/) and e-myco kit from LiliF Diagnostics.
Commonly misidentified lines (See ICLAC register)	N/A

Plants

Seed stocks	N/A
Novel plant genotypes	N/A
Authentication	N/A

Flow Cytometry

Plots

Confirm that:

☒ The axis labels state the marker and fluorochrome used (e.g. CD4-FITC).

☒ The axis scales are clearly visible. Include numbers along axes only for bottom left plot of group (a 'group' is an analysis of identical markers).

☒ All plots are contour plots with outliers or pseudocolor plots.

☒ A numerical value for number of cells or percentage (with statistics) is provided.

Methodology

Sample preparation	The DR-U2OS cell line containing a single integrated copy of the DR-GFP reporter was used. After indicated knockdown and treatment, U2OS were trypsinized and wash twice by PBS. Live cells were used for HR, SSA and NHEJ proficiency determination by counting the fraction of GFP-positive cells.<br>The Tet-DR-GFP cell line containing tet-DR-GFP cassette was used. After siRNA treatment, mCherry labeled I-SCEI were expressed in cells. After 62 hours, the cells were collected in PBS, and fixed in 1% PFA. GFP-positive cells were determined for HR proficiency of transcription positive status.(details were described in the Materials and Method.)
Instrument	BD FACS Calibur S instrument, LSRII
Software	Flowjo 10.6.2
Cell population abundance	All single live cells were used for analysis.

Gating strategy

For U2OS-DR-GFP reporter assay, untreated cells(GFP-negative) were used for gating and GFP-positive single cells were counted in the single live cell population.
For U2OS-Tet-on DR-GFP reporter assay, the normal cell population was gated by SSC-A and FSC-A. The HR rate was then calculated as the ratio of GFP-positive cell number to mCherry-positive cell number.

☒ Tick this box to confirm that a figure exemplifying the gating strategy is provided in the Supplementary Information.
