# Supplementary material for: HELZ-BRCA2 complex resolves R-loops to drive transcription-coupled homologous recombination
Source: Nat Commun. 2026 Jul 23;17:6969. doi: 10.1038/s41467-026-75088-4 (PMC13396364; doi:10.1038/s41467-026-75088-4)
Supplement: Supplementary file 3 — Transparent Peer Review file [file 41467_2026_75088_MOESM3_ESM.pdf]

nature portfolio

Peer Review File

HELZ-BRCA2 Complex Resolves R-loops to Drive Transcription-coupled Homologous Recombination

Corresponding Author: Professor Weixing Zhao

This file contains all reviewer reports in order by version, followed by all author rebuttals in order by version.

Version 0:

Reviewer comments:

Reviewer #1

(Remarks to the Author)
In this manuscript, Li et al describes a direct interaction between HELZ and BRCA2 and its role in R-loop homeostasis and transcription-coupled homologous recombination. The authors determined the ssRNA helicase activity of HELZ and how BRCA2 physically and functionally interacts with HELZ in vitro and in cells. They also showed evidence for its role in end resection during HR and demonstrated the important role of HELZ for survival of R-loop-rich cancer cells. It is a novel and interesting study. The manuscript is comprehensive, well-organized, and easy to follow. All experiments are expertly designed and executed. This reviewer supports its publication if the authors can adequately address the following concerns:

Abstract line 31: Is it ‘a’ or ‘the first’?

When DSS1 is first mentioned in the introduction (page1 line 50), more details should be included as it may potentially affect the HELZ R-loop resolvase activity.

Page 2 lines 80-81: This sentence (Below……repair.) seems unnecessary to me.

Figures 1d-1f: The interaction studies in these figures are convincing. It would be nice to see what cotransfections of the insect cells by different combinations of HELZ, BRCA2, and/or DSS1 baculoviruses will result in, as it is possible to have more insights into how they interact with each other. If the authors did it, it would be nice to include them. If not, it is optional.

Page 3 lines 119-123: It would be helpful to determine the minimum length requirement of ssRNA for HELZ to interact with. This experiment will be helpful for learning and explaining the protein-RNA complexes in the wells of EMSA (extended figures 2a-2c) and the stoichiometry of the complex formed between purified HELZ and R-loops. At the very least, the authors should give their best estimates about stoichiometry.

Page 3 lines 140-141: I would be careful about the claim of ‘the first’ (line 265 in Discussion too). The conclusion based on the data seems to be some like “purified HELZ unwinds RNA-DNA hybrid with a 5’-RNA overhang and BRCA2 stimulates its unwinding. Furthermore, DSS1’s role in HELZ-mediated unwinding should be specified or explained in greater detail. It can be helpful to explain why CTRB does not enhance the helicase activity.

Title and page 4 lines 188-198: I assume that co-transcriptional homologous recombination equals to transcription-coupled homologous recombination. To avoid confusion, it would be helpful to use only one term, either TC-HR or co-transcriptional HR but not both.

Page 5 line 233: R-loop-enriched or R-loop-rich?

Reviewer #2

(Remarks to the Author)
In this manuscript Li, Wu and colleagues are reporting a new interaction between the tumor suppressor BRCA2 and the putative RNA helicase HELZ. The manuscript then further describes the RNA helicase activity of HELZ both in vitro and in

cellulo. Then showing that HELZ binds to RNA:DNA hybrids at the level of DNA breaks sites to promote DNA repair by homologous recombination. Authors' results suggest that HELZ favors long-range resection by displacing R-loops, therefore impacting on homologous recombination efficiency. This model is interesting notably when DNA breaks occur at the level of transcribed genes, where RNA:DNA hybrids can form.

The interaction is properly validated and controlled with different constructs and in vitro and in cellulo validations. The interest of the paper is to characterize the activity of HELZ and its impact on DNA repair. Indeed, HELZ is very poorly described in the literature with only a few papers published and its function has remained largely unknown. The data is convincing and support the conclusions. The manuscript is well-written and structured. We only here below list minor comments that we think need to be clarified.

Minor issues.

- The main limitation of the paper is that it does not really address the specificity of HELZ compared to other ribonucleases or helicases that remove R-loops in cells. Notably we are thinking about Senataxin (work of Legube and others) and DDX5 (work of A. Carreira). Is HELZ redundant to these activities? Are they recruited to different genomic locations? Do they work at different times in the cell-cycle? Cells do already have RNase H1 and RNase H2, why aren't they sufficient to perform the work (to control the RNA:DNA hybrids level genome-wide). Especially RNase H2 (work of d'Adda di Fagagna) and DDX5 (A. Carreira) were reported to interact with BRCA2 too. These aspects, even if speculative, could be further discussed in the discussion. It is important to use and cite properly the recent and abundant literature on hybrids at DNA breaks to extend this discussion.

- The manuscript lacks a basic characterization of HELZ. In which tissue, cell-type is it expressed? (RNA and protein expression levels). Comparing non-cancerous and cancerous cells. This is important because most of the experiments are performed in Hela cells. Protein localization should be further shown with cell fractionation for instance.

- Resection can be better assessed by SMART assay or PCR based assays in a site-specific DNA break system, as developed in the lab of Dr Gaelle Legube.

- It is important to know whether HELZ status can impact on ATR/ATM DNA damage signaling.

- In term of structure and connection between parts, the section (page5, lines 221 to 231) could come earlier, before dissecting the mechanism into cells. "Role of HELZ helicase activity in R-loop resolution and HR repair ».

- Please, consider non-expert readers of your work. In the introduction, "homologous recombination (HR) » is not explained. Provide a description at least briefly. Similarly, « RFP- replication fork protection" is not explained ". Refer to the work of Pr Vincenzo Costanzo showing that in absence of RAD51, ssDNA gaps accumulate at the level of replication forks.

- From the introduction, it also lacks a small paragraph of why R-loop would be present at DNA breaks. Is it normal situation? Are they newly transcribed or already present transcripts? Be more precise on the mechanism of formation and the role of hybrids at DNA breaks. It is important to introduce the work on RNAs at breaks from D'Adda di Fagagna's and Legube's groups.

- Figure 2b : The representation of the data is confusing. We understand it is the result of a nice technique (atomic force microscopy), but why not add also in this figure the result of a S9.6 dot-plot experiment like in the extended figure 12d-e ? or the in vitro interaction with different nucleic acid structures of extended figure 2 showing that HELZ binds preferentially to ssRNA and R-loop structures?

- Figure 5 and all the IF results in general : The representation of the IF data is confusing. In the legend, it talks about 3 independent experiments but according to the plot, it looks like it's the result of only one experiment. If only one representative experiment is shown it should be stated in the figure legends.

- page 3, line 143. Please rephrase « HELZ is recruited to R-loops that is facilitated by BRCA2 in cells » with something like : « HELZ recruitment to R-loops is facilitated by BRCA2 in human(cancer) cells »

- Authors mention an amplification of HELZ. But is it correlated to overexpression in tumors ? A quick check on public databases could help (here or elsewhere https://tnmplot.com/analysis/).

-page 4, lines 198 « These results highlight the essential role of HELZ in the DNA damage response and efficient DSB repair by HR, particularly in co-transcriptional HR.. » typo with two dots.

-page 4, line 200 « Frist, we found », typo to correct.

-ref 13. This preprint is now published : please update your citations. Here the reference: PMID: 38244544 ; PMCID: PMC11188060 ; DOI: 10.1016/j.molcel.2023.12.025

Reviewer #3

(Remarks to the Author)
In the manuscript “HELZ-BRCA2 Complex Resolves R-loops to Drive Co-Transcriptional Homologous Recombination”, the authors report that HELZ is a strong BRCA2 interactor and acts as an R-loop resolvase in cancer cells. The study includes a large amount of experimental work that is well executed and presented clearly. The main idea is convincing, and the manuscript highlights the novel role of the HELZ–BRCA2 complex in co-transcriptional HR. Below, I have several concerns that should be addressed before the manuscript can be considered for publication in Nature Communications.

1. Extended Fig. 2a shows that HELZ has negligible DNA/RNA binding affinity. Yet HELZ alone can unwind a 5′ RNA–DNA flap structure that lacks a 5′ RNA overhang (Extended Fig. 4e). Can this be explained?

2. In Fig. 3d and Extended Fig. 6a, BRCA2 silencing looks nearly complete, yet ~50% of GFP-HELZ/TK-KR co-foci cells remain. Why? Does this suggest that additional factors help recruit HELZ to damage-induced R-loop sites?

3. More evidence is needed to show that BRCA2 and HELZ co-localize at R-loop–associated breaks. Silencing BRCA2 alone doesn’t seem sufficient to support the model in Fig. 6e.

4. In Extended Fig. 12a–b, it would be useful to compare HELZ protein levels in immortalized non-cancer cell lines (e.g., fibroblasts or epithelial cells) and quantify levels across the tested cancer vs. non-cancer cells.

5. If HELZ is highly expressed in certain cancers to counteract R-loop accumulation, one would expect higher HELZ levels to correlate with lower R-loop levels. So why do cancer cells still consistently show enriched R-loops?

6. If HELZ mainly functions in co-transcriptional HR of DNA DSBs (line 198), why are global R-loops significantly elevated in HELZ-silenced cancer cells even without exogenous DNA damage? Conversely, if HELZ also removes physiologically formed R-loops, then multiple GFP-HELZ foci should appear in the nucleus even without TK-KR (Fig. 3a). Also, note there are two commas at the end of line 198.

7. CPT treatment does not show enhanced BRCA2–HELZ interaction in Extended Fig. 1f, which seems inconsistent with Fig. 1b (BRCA2-IP).

8. Extended Fig. 1f: the asterisk marks endogenous HELZ, but the signal looks too weak to be convincing, and similar bands also appear in lanes 5 and 6.

9. Extended Fig. 1a: Is BirA directly fused to DSS1, or is it a BRCA2–DSS1 chimera? The schematic doesn’t match the description in the text.

Version 1:

Reviewer comments:

Reviewer #1

(Remarks to the Author)
All my concerns have been adequately addressed in the revised manuscript.

Reviewer #2

(Remarks to the Author)
Dear authors,

We have now checked your revised manuscript and your answers to reviewers comments, and we are very satisfied with your work and the way you adressed all concerns, including important new results, new controls and extended discussion.

We here only have one minor suggestion for you that we think is important for the strength of your article. You did a very nice experiment, shown in Fig R4, to directly address the impact of HELZ depletion on resection with a PCR-based assay. The result is extremely convincing and thus we suggest you to include it in the final manuscript.

Aside this minor suggestion, we are happy with the revision and we recommend the publication of this article.

Best wishes

Reviewer #3

(Remarks to the Author)
The authors have addressed all my remaining concerns, and the revised manuscript is significantly improved in both data

quality and clarity. As the conclusions are now fully supported by the results, I recommend accepting the manuscript for publication.

Reviewer #4

(Remarks to the Author)
I co-reviewed this manuscript with one of the reviewers who provided the listed reports. This is part of the Nature Communications initiative to facilitate training in peer review and to provide appropriate recognition for Early Career Researchers who co-review manuscripts.

Open Access This Peer Review File is licensed under a Creative Commons Attribution 4.0 International License, which permits use, sharing, adaptation, distribution and reproduction in any medium or format, as long as you give appropriate credit to the original author(s) and the source, provide a link to the Creative Commons license, and indicate if changes were made.
In cases where reviewers are anonymous, credit should be given to 'Anonymous Referee' and the source.
The images or other third party material in this Peer Review File are included in the article's Creative Commons license, unless indicated otherwise in a credit line to the material. If material is not included in the article's Creative Commons license and your intended use is not permitted by statutory regulation or exceeds the permitted use, you will need to obtain permission directly from the copyright holder.
To view a copy of this license, visit https://creativecommons.org/licenses/by/4.0/

Point by point response to reviewers' comments

We thank the reviewers for their positive comments and suggestions to improve the manuscript. We have responded to each concern with new experiments, analysis, and/or edits to the text. Each reviewer comment is shown in *italics/blue* and is followed by our response.

REVIEWER COMMENTS

Reviewer #1 (Remarks to the Author): In this manuscript, Li et al describes a direct interaction between HELZ and BRCA2 and its role in R-loop homeostasis and transcription-coupled homologous recombination. The authors determined the ssRNA helicase activity of HELZ and how BRCA2 physically and functionally interacts with HELZ in vitro and in cells. They also showed evidence for its role in end resection during HR and demonstrated the important role of HELZ for survival of R-loop-rich cancer cells. It is a novel and interesting study. The manuscript is comprehensive, well-organized, and easy to follow. All experiments are expertly designed and executed. This reviewer supports its publication if the authors can adequately address the following concerns:

Response: We thank Reviewer #1 for the positive assessment of our work and for recognizing the novelty, organization, and technical rigor of the study. We appreciate the reviewer's constructive comments and have addressed each concern through additional experiments, data reanalysis, and clarification within the revised manuscript. Detailed point-by-point responses follow below.

Abstract line 31: Is it 'a' or 'the first'?

Response: We thank the reviewer for the comment. We have revised the wording to use "an" reflecting that HELZ is an ssRNA-specific R-loop resolvase characterized in this context. The abstract and discussion have been updated accordingly. Although our data and the existing literature on other helicases suggest that HELZ may be the first resolvase with specificity for ssRNA and R-loops, unlike SETX, DDX5, and DHX9, a definitive conclusion cannot be made yet without direct side-by-side comparison.

When DSS1 is first mentioned in the introduction (page1 line 50), more details should be included as it may potentially affect the HELZ R-loop resolvase activity.

Response: We appreciate this suggestion. We have revised the Introduction to include additional details on DSS1 (Page 2 line 57-60 of the revised manuscript), including its roles in stabilizing BRCA2, retaining it in the nucleus, and regulating its DNA-binding specificity. These additions clarify why DSS1 may influence HELZ R-loop resolvase activity both *in vitro* and in cells.

Page 2 lines 80-81: This sentence (Below……repair.) seems unnecessary to me.

Response: We have removed the sentence from the revised manuscript to improve clarity and flow.

Figures 1d-1f: The interaction studies in these figures are convincing. It would be nice to see what cotransfections of the insect cells by different combinations of HELZ, BRCA2, and/or DSS1 baculoviruses will result in, as it is possible to have more insights into how they interact with each other. If the authors did it, it would be nice to include them. If not, it is optional.

Response: We appreciate the reviewer's thoughtful suggestion. We performed multiple combinations of baculovirus co-infection for complex purification (**Figure R1**). These experiments were consistent with our original results: HELZ associates with BRCA2 or BRCA2-DSS1 without detectable differences, and DSS1 does not bind HELZ, indicating that DSS1 does not significantly influence HELZ-BRCA2 complex formation. Because these additional datasets did not provide further mechanistic insight beyond Figures 1d-1f, we have not included them in the manuscript, but we would be happy to incorporate them upon request.

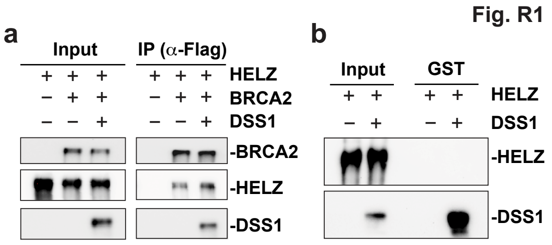

Figure R1. HELZ is a novel BRCA2 partner. a. Co-immunoprecipitation assay in Hi5 insect cells. Twin-strep-HELZ, Flag-BRCA2, and GST-DSS1 were co-infected in Hi5 cells as indicated. Cell lysates were subjected to immunoprecipitation using anti-Flag beads to isolate Flag-BRCA2 and its associated proteins. The input and IP fractions were analyzed by Western blotting using antibodies against FLAG, HELZ, and DSS1, respectively. b. GST pull-down assay in Hi5 insect cells. Twin-strep-HELZ and GST-DSS1 were expressed individually or together in Hi5 cells, and cell lysates were incubated with GST beads to capture GST-DSS1 complexes. Input and pull-down fractions were analyzed by Western blotting for HELZ and DSS1.

Page 3 lines 119-123: It would be helpful to determine the minimum length requirement of ssRNA for HELZ to interact with. This experiment will be helpful for learning and explaining the protein-RNA complexes in the wells of EMSA (extended figures 2a-2c) and the stoichiometry of the complex formed between purified HELZ and R-loops. At the very least, the authors should give their best estimates about stoichiometry.

Response: We have determined that the minimal ssRNA length required for HELZ binding is 15 nt (**Figure R2;** Supplementary Fig. 2c, d of the revised manuscript). Given this requirement, and the length of the ssRNA component and DNA bubble within the R-loop substrate, it is likely that

multiple HELZ molecules can bind simultaneously, consistent with our AFM observations showing multimeric HELZ-R-loop complexes. Because the amount of unbound HELZ present in the reaction cannot be precisely quantified under our EMSA conditions, we are unable to define an exact stoichiometry; however, the data support a model in which more than one HELZ molecule engages a single R-loop.

Fig. R2

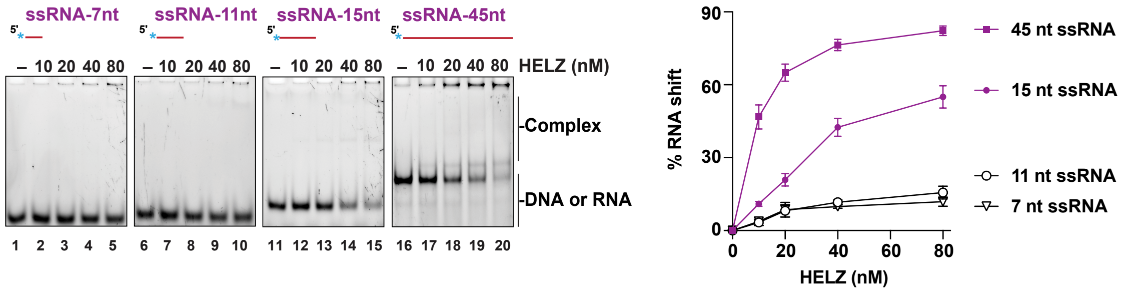

Figure R2. Minimal ssRNA length required for HELZ binding. Binding of 7nt, 11nt, 15 nt and 45nt ssRNA by HELZ as examined by EMSA (left panel). Quantification of the EMSA binding results (right panel). The data represent the mean ± SEM of at least 3 independent experiments.

Page 3 lines 140-141: I would be careful about the claim of 'the first' (line 265 in Discussion too). The conclusion based on the data seems to be some like "purified HELZ unwinds RNA-DNA hybrid with a 5'-RNA overhang and BRCA2 stimulates its unwinding. Furthermore, DSS1's role in HELZ-mediated unwinding should be specified or explained in greater detail. It can be helpful to explain why CTRB does not enhance the helicase activity.

Response: Thank you for this insightful comment. We have now removed the claim of "the first" (Page 3 line 147 and Page 6 line 268 and 282 of the revised manuscript) and incorporated an explanation in the Results section and added corresponding points to the Introduction and Discussion regarding the potential role of DSS1 (Page 3 line 142-143, Page 2 line 57-60 and Page 7 line 317-318 of the revised manuscript). Briefly, our results indicate that both the BRCA2 DNA-binding domain (DBD) and CTRB are required to stimulate HELZ helicase activity. Although CTRB is the primary HELZ-interacting region, it is not sufficient on its own. DSS1 modulates BRCA2-DBD's substrate specificity by suppressing dsDNA binding and promoting ssDNA engagement, which is essential for targeting the ssDNA region of R-loop structures. The lack of stimulation by CTRB alone likely reflects its inability to properly engage the R-loop substrate and therefore its inability to support HELZ recruitment. Thus, BRCA2-DBD and CTRB, together with DSS1, appear to be required to coordinate substrate presentation and promote HELZ-mediated unwinding.

Title and page 4 lines 188-198: I assume that co-transcriptional homologous recombination

equals to transcription-coupled homologous recombination. To avoid confusion, it would be helpful to use only one term, either TC-HR or co-transcriptional HR but not both.

Response: Thank you for pointing this out. We have standardized the terminology throughout the manuscript and now use "transcription-coupled HR" consistently.

Page 5 line 233: R-loop-enriched or R-loop-rich?

Response: We appreciate the reviewer's comment regarding terminology. In this context, we use "R-loop-enriched" to describe cancer cells with elevated R-loop levels, as this term more accurately reflects an experimentally defined enrichment of R-loop structures. "R-loop-enriched" is also consistent with common usage in the literature to denote cells or genomic regions identified through specific assays as having increased R-loop accumulation. We hope this clarification addresses the concern.

Reviewer #2 (Remarks to the Author): In this manuscript Li, Wu and colleagues are reporting a new interaction between the tumor suppressor BRCA2 and the putative RNA helicase HELZ. The manuscript then further describes the RNA helicase activity of HELZ both in vitro and in cellulo. Then showing that HELZ binds to RNA:DNA hybrids at the level of DNA breaks sites to promote DNA repair by homologous recombination. Authors' results suggest that HELZ favors long-range resection by displacing R-loops, therefore impacting on homologous recombination efficiency. This model is interesting notably when DNA breaks occur at the level of transcribed genes, where RNA:DNA hybrids can form.

The interaction is properly validated and controlled with different constructs and in vitro and in cellulo validations. The interest of the paper is to characterize the activity of HELZ and its impact on DNA repair. Indeed, HELZ is very poorly described in the literature with only a few papers published and its function has remained largely unknown. The data is convincing and support the conclusions. The manuscript is well-written and structured. We only here below list minor comments that we think need to be clarified.

Response: We thank Reviewer #2 for the positive evaluation of our work and for recognizing the significance of identifying the BRCA2-HELZ interaction and characterizing HELZ's role in R-loop regulation and homologous recombination. We appreciate the reviewer's thoughtful comments. All points listed have been carefully addressed through additional clarification, textual revisions, or inclusion of new data where appropriate. Detailed point-by-point responses are provided below.

Minor issues.

- The main limitation of the paper is that it does not really address the specificity of HELZ compared to other ribonucleases or helicases that remove R-loops in cells. Notably we are

thinking about Senataxin (work of Legube and others) and DDX5 (work of A. Carreira). Is HELZ redundant to these activities? Are they recruited to different genomic locations? Do they work at different times in the cell-cycle? Cells do already have RNase H1 and RNase H2, why aren't they sufficient to perform the work (to control the RNA:DNA hybrids level genome-wide). Especially RNase H2 (work of d'Adda di Fagagna) and DDX5 (A. Carreira) were reported to interact with BRCA2 too. These aspects, even if speculative, could be further discussed in the discussion. It is important to use and cite properly the recent and abundant literature on hybrids at DNA breaks to extend this discussion.

Response: We thank the reviewer for raising this important point. Our study focuses on identifying HELZ as a BRCA2 partner and a previously unrecognized ssRNA-directed helicase that removes R-loops, and on defining the resulting consequences for homologous recombination. We totally agree that comparing HELZ with other R-loop resolving enzymes—such as Senataxin (SETX), RNase H1, DDX5, and RNase H2, including the latter two known to also interact with BRCA2—is an important direction for future work.

In the revised Discussion, we now explicitly compare HELZ with other R-loop regulating helicases, including SETX, DDX5 and DHX9, and discuss their specificity, recruitment, and functional timing (see "**HELZ helicase activity and its role in R-loop resolution**"; Page 6 and 7). We emphasize that HELZ is an ssRNA-directed R-loop helicase whose depletion leads to global and DSB-associated R-loop accumulation and selectively impairs DNA2 loading and long-range resection, whereas SETX, DDX5, and DHX9 act at distinct stages of HR (fidelity control, post-resection hybrid clearance, and BRCA1-CtIP-dependent initiation, respectively).

Regarding why RNase H1 and H2 are not sufficient to control RNA-DNA hybrids genome-wide, our full rationale/speculation is outlined below, while a more concise version has been incorporated into the revised Discussion (Page 6 line 286-288 of the revised manuscript) to maintain focus and flow.

RNase H1/2 efficiently cleave RNA within relatively regular, accessible RNA-DNA duplexes but do not effectively act on chromatin-embedded, protein-stabilized, or structurally complex R-loops (*e.g.*, those containing G-quadruplexes, RNA secondary structures, or large ribonucleoprotein assemblies). In these contexts, RNA helicases act first to remodel or partially unwind the hybrid—by disrupting secondary structures and displacing bound proteins—and thereby either resolve the R-loop directly or convert it into simpler RNA-DNA duplex intermediates that then become competent substrates for RNase H. This division of labor is supported by genetic evidence: loss of RNase H causes global R-loop accumulation even when helicases are intact, whereas depletion of SETX, DDX5, or HELZ still leads to substantial R-loop buildup and repair defects in the presence of functional RNase H1/2, and combined perturbations are often synthetically deleterious. We

further note that RNase H2 is tightly coupled to S/G2 and replication-linked lesions (and its recruitment/function are at least partly BRCA2- and damage-signaling-dependent), whereas many of the R-loops relevant to our study arise in transcription-coupled DSB contexts that are not strictly replication driven. In BRCA2-deficient cells, RNase H2 is expressed but mislocalized, further restricting its ability to act at damage-associated R-loops. RNase H1, although not strongly cell-cycle regulated, faces similar accessibility constraints at protein-bound or structurally complex hybrids. Moreover, RNase H complexes are integrated into damage-response networks that are controlled by post-translational modifications, adding an additional layer of regulation over when and where RNase H can act. Together, these considerations support a model in which RNase H1/2 provide essential but context-limited hybrid degradation, while helicases including BLM, AQR, FANCM, HELZ, SETX, DDX5, and DHX9 are required to remodel distinct classes of R-loops at specific genomic sites and repair stages, explaining why RNase H activity alone is insufficient to maintain RNA-DNA hybrid homeostasis genome-wide.

- The manuscript lacks a basic characterization of HELZ. In which tissue, cell-type is it expressed? (RNA and protein expression levels). Comparing non-cancerous and cancerous cells. This is important because most of the experiments are performed in Hela cells. Protein localization should be further shown with cell fractionation for instance.

Response: We thank the reviewer for this important question. HELZ is broadly expressed at the RNA level across human tissues, with higher transcript levels in proliferative and hematopoietic compartments according to publicly available datasets (*e.g.*, TCGA/GTEx). Consistent with this, our Western blot analysis shows detectable HELZ protein in non-cancerous cell lines, including MCF10A (immortalized mammary epithelial cells), HSF (immortalized human splenic fibroblasts), BJ (immortalized human foreskin fibroblasts), and RPE-1 (immortalized retinal pigment epithelial cells), with generally higher expression in mammary-derived cells compared with fibroblasts. Among the cancer cell lines examined, HELZ protein is particularly elevated in ER^+ breast cancer cells (*e.g.*, MCF7) and in several sarcoma lines (**Figure R3a;** Supplementary fig. 12b of the revised manuscript). These data indicate that HELZ is expressed in multiple normal tissues but tends to be upregulated in specific cancer contexts, especially mammary and certain hematologic cancers (*e.g.,* Acute Myeloid Leukemia (AML); see **Figure R6**).

To assess subcellular localization, we performed cytoplasmic-nuclear fractionation (**Figure R3b;** Supplementary fig. 6c of the revised manuscript). HELZ displayed an approximately equal distribution between the cytoplasmic and nuclear fractions, indicating potential functions in both compartments.

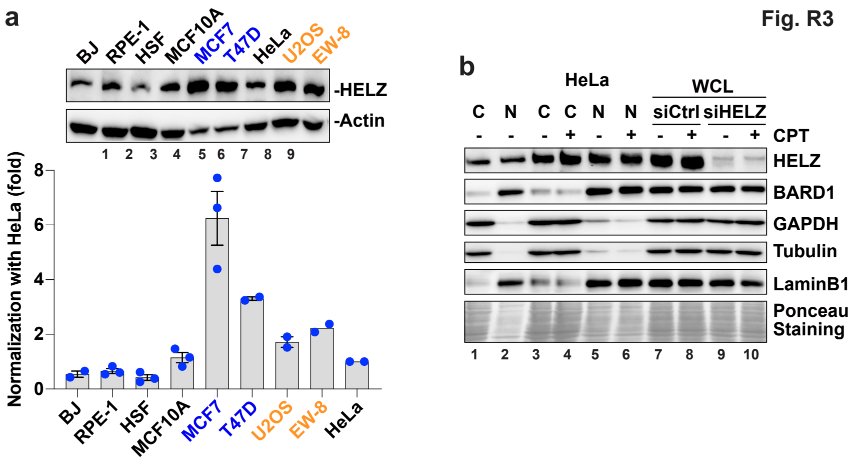

Figure R3. HELZ is upregulated in cancer cells and is localized in both cytoplasmic and nucleus. a. Western blot to check HELZ expression in various cancer and normal cells (top panel) and HELZ expression levels were quantified and normalized to actin (bottom panel). b. Western blot to verify the nuclear localization of endogenous HELZ in HeLa cells. The cytoplasmic (C)and nuclear (N) fractions were analyzed. Lamin B1 and BARD1 mark nuclear fraction; Tubulin and GAPDH mark cytoplasmic fraction. Ponceau S staining was used as a loading control. WCL stands for whole cell lysate.

- Resection can be better assessed by SMART assay or PCR based assays in a site-specific DNA break system, as developed in the lab of Dr Gaelle Legube.

Response: We appreciate this suggestion and therefore proceeded with a qPCR-based end-resection assay at a site-specific DSB (**Figure R4**), using the method developed by Dr. Gaëlle Legube's laboratory. Consistent with our RPA and BrdU foci analyses, we observed a clear defect in DNA end resection upon HELZ depletion under the qPCR-based end-resection assay.

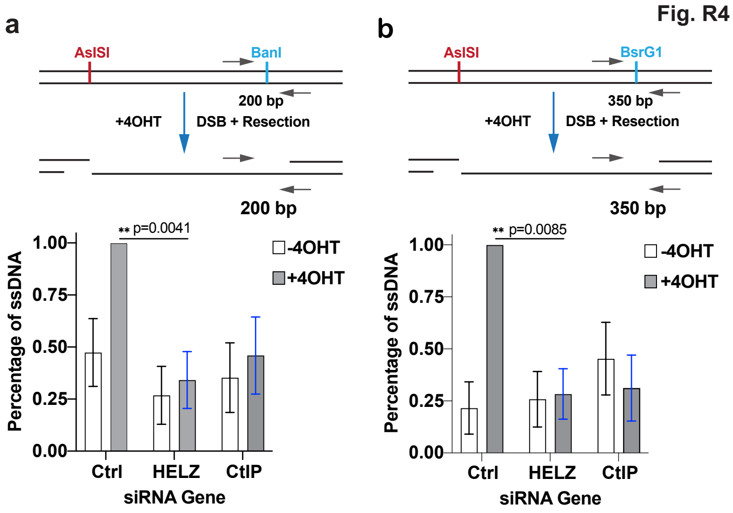

Figure R4. HELZ is required for DNA end resection. a. DIvA (AsiSI-ER-U2OS) cells were treated with 300 nM 4-OHT for 4 h or mock, genomic DNA (gDNA) was extracted and digested or mock digested with BanI overnight. DNA end resection adjacent to DSB was measured by qPCR from three independent experiments. **, P ≤ 0.01 (student's t-test). B. DIvA (AsiSI-ER-U2OS) cells were treated with 300 nM 4-OHT for 4 h or mock, gDNA was extracted and digested or mock digested with BsrG1 overnight. DNA end resection adjacent to DSB was measured by qPCR from three independent experiments. **, P ≤ 0.01 (student's t-test).

- It is important to know whether HELZ status can impact on ATR/ATM DNA damage signaling.

Response: Thank you for highlighting this important point. We examined ATR- and ATM-pathway activation by Western blot following HELZ knockdown (**Figure R5;** Supplementary fig. 10f of the revised manuscript). HELZ depletion resulted in reduced pATR and pChk1 levels, together with increased pATM and γH2AX after 6 Gy IR. These results are consistent with HELZ promoting DNA end resection: diminished resection leads to reduced ssDNA/RPA formation and weakened ATR-Chk1 signaling, whereas delayed repair maintains elevated γH2AX and pATM levels.

Fig. R5

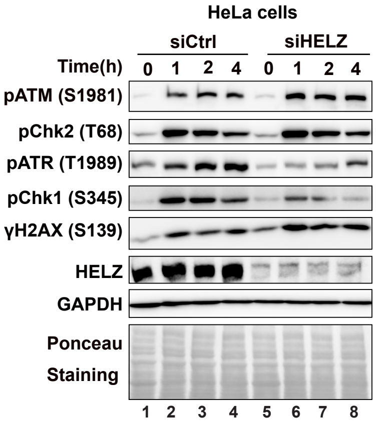

Figure R5. HELZ depletion causes the reduction of pATR and pChk1 and the accumulation of pATM and γH2AX. Western blot analysis to assess DNA damage response signaling upon HELZ depletion in HeLa cells. Antibodies against pATM (S1981), pChk2 (T68), pATR(T1989), pChk1(S345), γH2AX (S139) and HELZ were used for probing. GAPDH blot and Ponceau staining served as loading controls.

- In term of structure and connection between parts, the section (page5, lines 221 to 231) could come earlier, before dissecting the mechanism into cells. "Role of HELZ helicase activity in R-loop resolution and HR repair ».

Response: We agree with the reviewer's suggestion. We have moved this section, along with the corresponding data originally presented in Fig. 6a-c, to a new Fig. 5 so that the role of HELZ

helicase activity in R-loop resolution and HR is presented earlier, prior to the end-resection data.

- Please, consider non-expert readers of your work. In the introduction, "homologous recombination (HR) » is not explained. Provide a description at least briefly. Similarly, « RFP-replication fork protection" is not explained ". Refer to the work of Pr Vincenzo Costanzo showing that in absence of RAD51, ssDNA gaps accumulate at the level of replication forks.

Response: We appreciate this recommendation. In the revised Introduction, we now provide brief definitions of homologous recombination (HR) and replication-fork protection (RFP) to ensure accessibility for non-expert readers (Page 1 line 48-49; Page 2 line 56-57). We also incorporated work from Prof. Vincenzo Costanzo and others showing that RAD51 or BRCA2 deficiency leads to ssDNA-gap accumulation at replication forks, highlighting the importance of BRCA2-mediated RAD51 loading in HR, ssDNA-gap suppression, and RFP.

- From the introduction, it also lacks a small paragraph of why R-loop would be present at DNA breaks. Is it normal situation? Are they newly transcribed or already present transcripts? Be more precise on the mechanism of formation and the role of hybrids at DNA breaks. It is important to introduce the work on RNAs at breaks from D'Adda di Fagagna's and Legube's groups.

Response: We thank the reviewer for this valuable suggestion. In the revised Introduction, we have included a new section summarizing that R-loops may arise from either pre-existing transcripts or from damage-induced de novo transcription (Page 1 line 46-47), as shown in studies from the d'Adda di Fagagna and Legube groups. These additions provide clearer context for the presence and functional significance of R-loops at DSBs.

- Figure 2b : The representation of the data is confusing. We understand it is the result of a nice technique (atomic force microscopy), but why not add also in this figure the result of a S9.6 dot-plot experiment like in the extended figure 12d-e ? or the in vitro interaction with different nucleic acid structures of extended figure 2 showing that HELZ binds preferentially to ssRNA and R-loop structures?

Response: We appreciate this helpful suggestion. To improve clarity, we have moved the EMSA binding results from the original Supplementary fig. 2a into Fig. 2a so that they appear alongside the AFM data in Fig. 2b. This now provides a direct comparison of HELZ's binding preference for ssRNA and R-loop structures. We believe this integration addresses the reviewer's concern and makes the figure easier to interpret.

- Figure 5 and all the IF results in general: The representation of the IF data is confusing. In the legend, it talks about 3 independent experiments but according to the plot, it looks like it's the

result of only one experiment. If only one representative experiment is shown it should be stated in the figure legends.

Response: Thank you for pointing this out. The graphs in these figures present one representative experiment. We have revised the figure legends to clearly indicate this and to avoid confusion with the number of biological replicates performed.

- page 3, line 143. Please rephrase « HELZ is recruited to R-loops that is facilitated by BRCA2 in cells » with something like : « HELZ recruitment to R-loops is facilitated by BRCA2 in human(cancer) cells »

Response: We thank the reviewer for the suggestion. We have revised the subheading to: “HELZ is recruited to R-loops, and this recruitment is facilitated by BRCA2 in cells.

- Authors mention an amplification of HELZ. But is it correlated to overexpression in tumors? A quick check on public databases could help (here or elsewhere https://tnmplot.com/analysis/).

Response: We thank the reviewer for this helpful suggestion. In addition to the cBioPortal analysis presented in Supplementary Fig. 12a of the original and revised manuscript, we examined HELZ expression using TNMplot, GEPIA2, and the Human Protein Atlas (HPA) (**Figure R6**). These datasets indicate that HELZ is overexpressed in several tumor types compared with normal tissues, although this pattern is not uniform across all cancers.

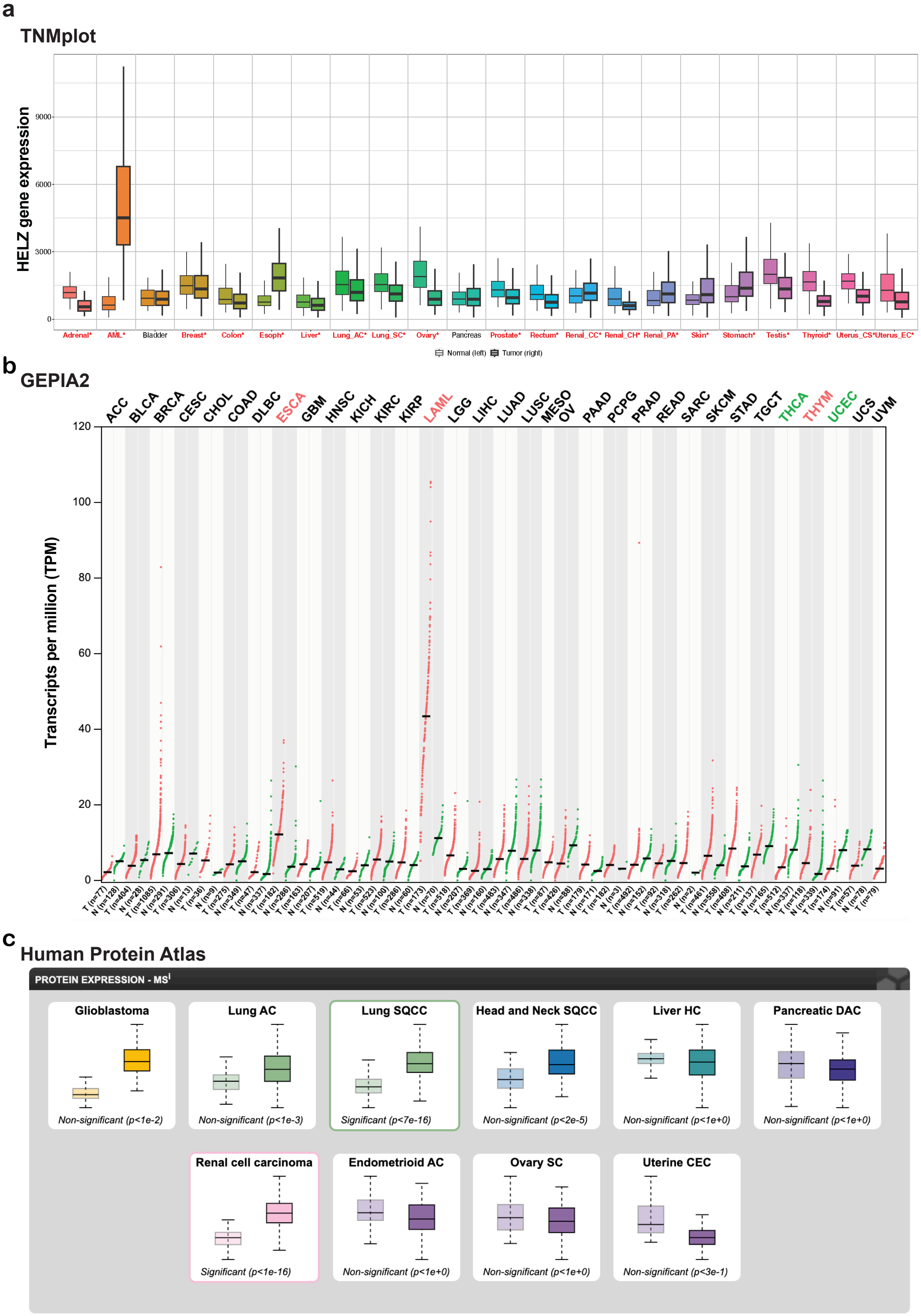
a
TNMplot
HELZ gene expression
9000
6000
3000
0
Adrenal*
AML*
Bladder
Breast*
Colon*
Esoph*
Liver*
Lung_AC*
Lung_SC*
Ovary*
Pancreas
Prostate*
Rectum*
Renal_CC*
Renal_CH*
Renal_PA*
Skin*
Stomach*
Testis*
Thyroid*
Uterus_CS*
Uterus_EC*
Normal (left)
Tumor (right)
b
GEPIA2
ACC
BLCA
BRCA
CESC
CHOL
COAD
DLBC
ESCA
GBM
HNSC
KICH
KIRC
KIRP
LAML
LGG
LIHC
LUAD
LUSC
MESO
OV
PAAD
PCPG
PRAD
READ
SARC
SKCM
STAD
TGCT
THCA
THYM
UCEC
UCS
UVM
Transcripts per million (TPM)
120
100
80
60
40
20
0
c
Human Protein Atlas
PROTEIN EXPRESSION - MS
Glioblastoma
Non-significant (p<1e-2)
Lung AC
Non-significant (p<1e-3)
Lung SQCC
Significant (p<7e-16)
Head and Neck SQCC
Non-significant (p<2e-5)
Liver HC
Non-significant (p<1e+0)
Pancreatic DAC
Non-significant (p<1e+0)
Renal cell carcinoma
Significant (p<1e-16)
Endometrioid AC
Non-significant (p<1e+0)
Ovary SC
Non-significant (p<1e+0)
Uterine CEC
Non-significant (p<3e-1)

Figure R6. HELZ expression in tumor vs non-tumor tissue. a. The pan-cancer box plot (TNMplot) shows the expression of HELZ across normal and cancer tissues using RNA-Seq data from GEO, GTEx, TCGA, and TARGET repositories. b. The dot plot (GEPIA2) shows the expression of HELZ across all tumor samples and paired normal tissues using RNA-seq data from TCGA/GTEx. Each dot represents the expression of a sample. c. The box plot shows the HELZ protein level across tumor samples and paired normal tissues from the Human Protein Atlas.

-page 4, lines 198 « These results highlight the essential role of HELZ in the DNA damage response and efficient DSB repair by HR, particularly in co-transcriptional HR.. » typo with two dots.

Response: We thank the reviewer for the careful observation. We have removed the extra period.

-page 4, line 200 « Frist, we found », typo to correct.

Response: We thank the reviewer for the careful observation. We have corrected "Frist" to "First."

-ref 13. This preprint is now published : please update your citations. Here the reference: PMID: 38244544 ; PMCID: PMC11188060 ; DOI: 10.1016/j.molcel.2023.12.025

Response: We have updated the citation (ref 22 of the revised manuscript) accordingly.

Reviewer #3 (Remarks to the Author): In the manuscript "HELZ-BRCA2 Complex Resolves R-loops to Drive Co-Transcriptional Homologous Recombination", the authors report that HELZ is a strong BRCA2 interactor and acts as an R-loop resolvase in cancer cells. The study includes a large amount of experimental work that is well executed and presented clearly. The main idea is convincing, and the manuscript highlights the novel role of the HELZ–BRCA2 complex in co-transcriptional HR. Below, I have several concerns that should be addressed before the manuscript can be considered for publication in Nature Communications.

Response: We thank Reviewer #3 for the positive assessment of our work and the constructive comments. We appreciate the acknowledgment of the experimental depth and clarity of the study. We have carefully considered all concerns raised and have revised the manuscript accordingly. Detailed point-by-point responses are provided below.

1. Extended Fig. 2a shows that HELZ has negligible DNA/RNA binding affinity. Yet HELZ alone can unwind a 5′ RNA–DNA flap structure that lacks a 5′ RNA overhang (Extended Fig. 4e). Can this be explained?

Response: We thank the reviewer for this insightful question. Although HELZ shows minimal binding to isolated RNA-DNA hybrids without an overhang, it binds efficiently to the RNA-DNA flap substrates used in the unwinding assays when examined at 4 °C (**Figure R7**). These flap structures are partially unstable and undergo transient RNA unwinding at 37 °C (Lanes 2, 6, 7 and 8 of **Figure R7a**), briefly exposing short ssRNA segments. We believe that HELZ could load onto these exposed regions to initiate unwinding even without a defined 5′ RNA overhang or that HELZ could stabilize the ssRNA product. This mechanistic explanation could account for the difference between the EMSA binding result of RNA-DNA hybrid and the unwinding activity on RNA-DNA flap (**Figure R7a**; Supplementary fig. 4e)

Because the RNA-DNA flap-binding EMSA was performed at 4 °C, whereas the other EMSAs were all performed at 37 °C, we did not include this comparison in the manuscript to avoid misinterpretation. We can add these data if the reviewer considers them necessary.

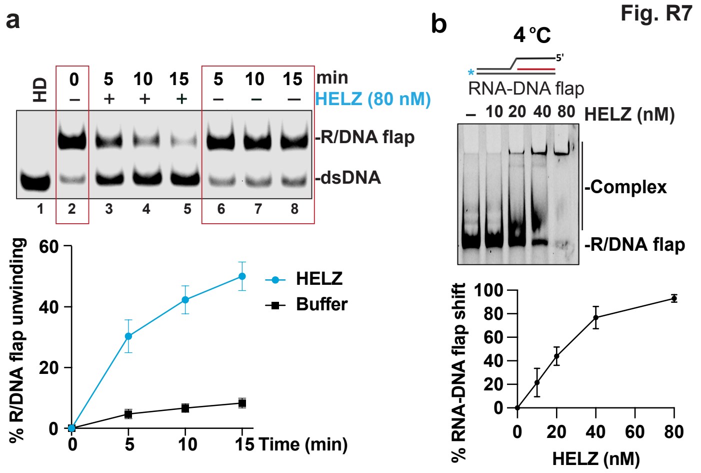

Figure R7. RNA-DNA flap binding and unwinding by HELZ. a. Native PAGE gel showing representative RNA-DNA flap unwinding by HELZ (80 nM) at 5, 10, and 15 mins (top). Quantification of RNA-DNA flap unwinding experiments by HELZ at 5, 10, and 15 mins in comparison to buffer alone (bottom). The data represent the mean ± SEM of three independent experiments. b. Binding of RNA-DNA flap by HELZ as examined by EMSA (top panel). Quantification of the EMSA binding results (right panel). The data represent the mean ± SEM of two independent experiments.

2. In Fig. 3d and Extended Fig. 6a, BRCA2 silencing looks nearly complete, yet ~50% of GFP-HELZ/TK-KR co-foci cells remain. Why? Does this suggest that additional factors help recruit HELZ to damage-induced R-loop sites?

Response: We agree with the reviewer that HELZ recruitment is not solely dependent on BRCA2. The residual GFP-HELZ/TA-KR co-foci observed after BRCA2 silencing likely reflect BRCA2-independent mechanisms that can still guide HELZ to damage-induced R-loop sites. HELZ may

be recruited through additional protein factors or through its intrinsic affinity for ssRNA/R-loop structures. Identifying these additional regulators will be an important direction for future studies, and we have noted this point in the result section of the revised manuscript (Page 4 line 167-169).

3. More evidence is needed to show that BRCA2 and HELZ co-localize at R-loop–associated breaks. Silencing BRCA2 alone doesn't seem sufficient to support the model in Fig. 6e.

Response: We have added additional evidence supporting BRCA2-HELZ co-localization at R-loop associated breaks. Immunofluorescence analysis shows clear co-recruitment of HELZ and BRCA2 at TA-KR induced damage sites (**Figure R8a**). Moreover, proximity ligation assays (PLA) demonstrate a significant increase in HELZ-BRCA2 interaction following camptothecin (CPT) treatment (**Figure R8b**), consistent with enhanced co-localization at R-loop enriched DNA damage sites. These results, included in Supplementary fig. 6a, d of the revised manuscript, further support the model proposed in original Fig. 6e (Fig.7d of the revised manuscript).

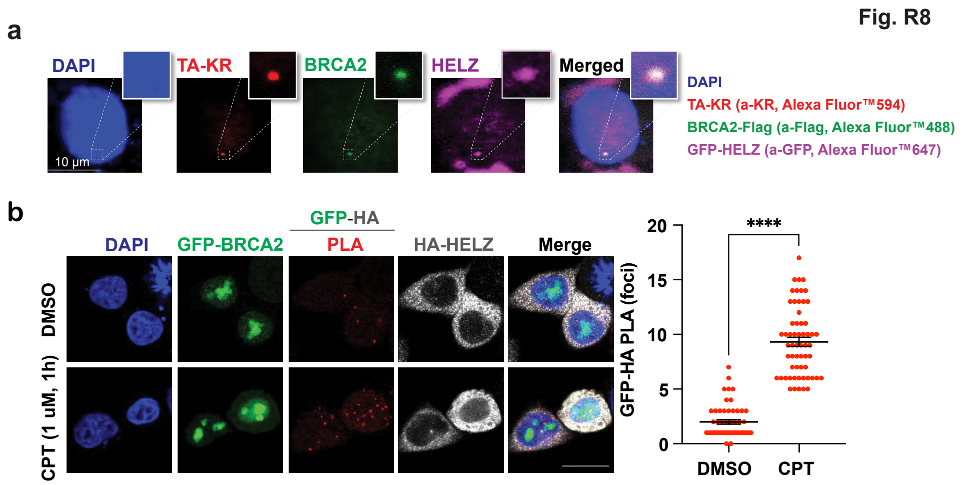

Figure R8. The colocalization of HELZ and BRCA2 with R-loops in cells. a. U2OS-TRE cells transfected with pBROAD3 TA-KR and the expression vector of GFP-HELZ and MBP-BRCA2-Flag were light-activated and recovered 20 min before fixation. Representative images of GFP and Flag foci at sites of TA-KR were shown. Flag-BRCA2 (Alexa Fluor™ 488) and HA-HELZ (Alexa Fluor™ 647) detected with anti-Flag and anti-HA antibodies. DAPI marks nuclei (blue); TA- b. Representative micrographs of PLA foci (red) showing colocalization between BRCA2 (α-GFP). and HELZ (α-HA) in the nuclei of HeLa cells transient expressing BRCA2-GFP and HA-HELZ after the treatment of DMSO or CPT (1 µM; 1 h) (left). Blue: DAPI. Green: BRCA2-GFP. Grey: HA-HELZ. Scale bar: 10 µm. Average number (±SEM) of PLA foci from 50-60 nuclei were plotted (right). Statistical analysis was done with the two-sided student's t-test, ****$P \leq 0.0001$.

4. In Extended Fig. 12a–b, it would be useful to compare HELZ protein levels in immortalized

non-cancer cell lines (e.g., fibroblasts or epithelial cells) and quantify levels across the tested cancer vs. non-cancer cells.

Response: Thanks for the suggestion. As noted in our response to Reviewer #2 (see **Fig. R3**), we performed Western blot analyses to compare HELZ expressions across non-cancerous (MCF10A, HSF, BJ, and RPE-1) and cancerous cell lines (MCF7, T47D, U2OS and EW-8). HELZ expression was generally higher in mammary-derived cells than in other cells, with the highest levels observed in the ER+ breast cancer line MCF7, suggesting tissue- and tumor-associated variation in HELZ abundance.

5. If HELZ is highly expressed in certain cancers to counteract R-loop accumulation, one would expect higher HELZ levels to correlate with lower R-loop levels. So why do cancer cells still consistently show enriched R-loops?

Response: We appreciate this thoughtful question. Elevated HELZ expression in cancer cells likely reflects a compensatory response to increased transcriptional activity and heightened R-loop formation rather than an ability to fully suppress R-loops. Cancer cells exhibit high proliferation rates, strong transcriptional upregulation, replication stress, and oncogene-driven transcription-replication conflicts, all of which generate R-loop loads that exceed the capacity of any single resolvase. Thus, even when HELZ is upregulated, its activity might be insufficient to fully counterbalance this elevated burden, allowing high HELZ levels to coexist with enriched R-loops.

In addition, HELZ might act on only a subset of R-loops. Certain R-loop prone loci, such as telomeres, rDNA repeats, and other highly transcribed regions, may accumulate hybrids that escape HELZ-mediated processing or require other factors such as SETX, DDX5, or RNase H2 for efficient resolution. Finally, HELZ abundance does not necessarily equate to HELZ activity; post-translational regulation, subcellular mislocalization, or restricted accessibility by R-loop binding protein or chromatin structures may limit its ability to access or unwind R-loops in cells.

6. If HELZ mainly functions in co-transcriptional HR of DNA DSBs (line 198), why are global R-loops significantly elevated in HELZ-silenced cancer cells even without exogenous DNA damage? Conversely, if HELZ also removes physiologically formed R-loops, then multiple GFP-HELZ foci should appear in the nucleus even without TK-KR (Fig. 3a). Also, note there are two commas at the end of line 198.

Response: We thank the reviewer for raising this important point. HELZ participates in co-transcriptional HR at DSBs, but our data does not indicate that its R-loop resolving activity is restricted to break sites. Loss of HELZ leads to elevated global R-loops because physiologically generated, endogenous damage-induced, or replication stress-triggered R-loops also require HELZ for efficient turnover, independent of exogenous DNA damage. However, these endogenous R-loops are broadly distributed and generally below the spatial resolution needed to form discrete nuclear GFP-HELZ foci detectable by microscopy. In contrast, TA-KR induces highly localized,

concentrated R-loop formation at DSBs, producing the robust foci observed in Fig. 3a. We have clarified this distinction in the revised manuscript (Page 4 line 157-158, 162-163, 175-177 and 181-182) and corrected the duplicated comma at line 198.

7. CPT treatment does not show enhanced BRCA2–HELZ interaction in Extended Fig. 1f, which seems inconsistent with Fig. 1b (BRCA2-IP).

Response: Thank you for pointing this out. We have now quantified the BRCA2-to-HELZ binding ratio, and the results indicate a clear enhancement upon CPT treatment (**Figure R9;** Supplementary fig. 1f of the revised manuscript).

Fig. R9

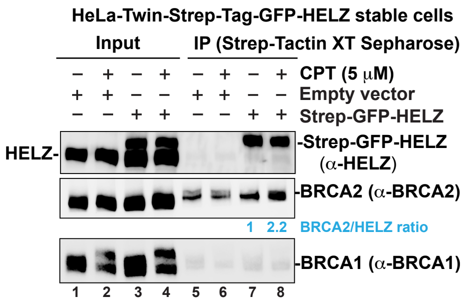

Figure R9. HELZ-BRCA2 interaction increases following CPT treatment. Streptavidin-coated beads were used to pull down GFP-Strep-HELZ in HeLa-HELZ stable cells with CPT (5 uM) for 4h. Anti-BRCA2, HELZ, and BRCA1 antibodies were used for the Western Blot. The ratio of HELZ to BRCA2 from the IP experiment is shown in cyan.

8. Extended Fig. 1f: the asterisk marks endogenous HELZ, but the signal looks too weak to be convincing, and similar bands also appear in lanes 5 and 6.

Response: The asterisk denotes endogenous HELZ in lanes 1-4 (see **Figure R9**). The faint, similarly migrating bands observed in lanes 5-6 and 7-8 are likely nonspecific signals. Because no antibody suitable for HELZ immunoprecipitation is available, we enriched only the ectopically expressed Twin-Strep-GFP-HELZ using anti-Strep beads, which should exclude endogenous HELZ in the IP samples (lanes 5-6) except for nonspecific binding. To minimize confusion, we have now explicitly labeled endogenous HELZ on the left side of the blot in the revised Supplementary fig. 1f.

9. Extended Fig. 1a: Is BirA directly fused to DSS1, or is it a BRCA2–DSS1 chimera? The schematic doesn't match the description in the text.

Response: Thank you for pointing this out. The construct used is BirA directly fused to DSS1. We have clarified the schematic (Supplementary fig.1a of the revised manuscript).

Point by point response to reviewers' comments

We thank the reviewers for their very positive comments on our revision. In response to the suggestion from Reviewer #2 to include PCR-based end-resection data, we have added these results as Supplementary Fig. 10f and 10g in the revised manuscript.

REVIEWERS' COMMENTS

Reviewer #1 (Remarks to the Author):
All my concerns have been adequately addressed in the revised manuscript.

Reviewer #2 (Remarks to the Author):
Dear authors,
We have now checked your revised manuscript and your answers to reviewers comments, and we are very satisfied with your work and the way you addressed all concerns, including important new results, new controls and extended discussion.
We here only have one minor suggestion for you that we think is important for the strength of your article. You did a very nice experiment, shown in Fig R4, to directly address the impact of HELZ depletion on resection with a PCR-based assay. The result is extremely convincing and thus we suggest you to include it in the final manuscript.
Aside this minor suggestion, we are happy with the revision and we recommend the publication of this article.
Best wishes

Reviewer #3 (Remarks to the Author):
The authors have addressed all my remaining concerns, and the revised manuscript is significantly improved in both data quality and clarity. As the conclusions are now fully supported by the results, I recommend accepting the manuscript for publication.

Reviewer #4 (Remarks to the Author):
I co-reviewed this manuscript with one of the reviewers who provided the listed reports. This is part of the Nature Communications initiative to facilitate training in peer review and to provide appropriate recognition for Early Career Researchers who co-review manuscripts.
